# Causal inference regulates audiovisual spatial recalibration via its influence on audiovisual perception

**DOI:** 10.1371/journal.pcbi.1008877

**Published:** 2021-11-15

**Authors:** Fangfang Hong, Stephanie Badde, Michael S. Landy

**Affiliations:** 1 Department of Psychology, New York University, New York City, New York, United States of America; 2 Department of Psychology, Tufts University, Medford, Massachusetts, United States of America; 3 Center for Neural Science, New York University, New York City, New York, United States of America; Durham University, UNITED KINGDOM

## Abstract

To obtain a coherent perception of the world, our senses need to be in alignment. When we encounter misaligned cues from two sensory modalities, the brain must infer which cue is faulty and recalibrate the corresponding sense. We examined whether and how the brain uses cue reliability to identify the miscalibrated sense by measuring the audiovisual ventriloquism aftereffect for stimuli of varying visual reliability. To adjust for modality-specific biases, visual stimulus locations were chosen based on perceived alignment with auditory stimulus locations for each participant. During an audiovisual recalibration phase, participants were presented with bimodal stimuli with a fixed perceptual spatial discrepancy; they localized one modality, cued after stimulus presentation. Unimodal auditory and visual localization was measured before and after the audiovisual recalibration phase. We compared participants’ behavior to the predictions of three models of recalibration: (a) Reliability-based: each modality is recalibrated based on its relative reliability—less reliable cues are recalibrated more; (b) Fixed-ratio: the degree of recalibration for each modality is fixed; (c) Causal-inference: recalibration is directly determined by the discrepancy between a cue and its estimate, which in turn depends on the reliability of both cues, and inference about how likely the two cues derive from a common source. Vision was hardly recalibrated by audition. Auditory recalibration by vision changed idiosyncratically as visual reliability decreased: the extent of auditory recalibration either decreased monotonically, peaked at medium visual reliability, or increased monotonically. The latter two patterns cannot be explained by either the reliability-based or fixed-ratio models. Only the causal-inference model of recalibration captures the idiosyncratic influences of cue reliability on recalibration. We conclude that cue reliability, causal inference, and modality-specific biases guide cross-modal recalibration indirectly by determining the perception of audiovisual stimuli.

## Author summary

Audiovisual recalibration of spatial perception occurs when we receive audiovisual stimuli with a systematic spatial discrepancy. The brain must determine to which extent both modalities should be recalibrated. In this study, we scrutinized the mechanisms the brain employs to do so. To this aim, we conducted a classical audiovisual recalibration experiment in which participants were adapted to spatially discrepant audiovisual stimuli. The visual component of the bimodal stimulus was either less, equally, or more reliable than the auditory component. We measured the amount of recalibration by computing the difference between participants’ unimodal localization responses before and after the audiovisual recalibration. Across participants, the influence of visual reliability on auditory recalibration varied fundamentally. We compared three models of recalibration. Only a causal-inference model of recalibration captured the diverse influences of cue reliability on recalibration found in our study, this model is also able to replicate contradictory results found in previous studies. In this model, recalibration depends on the discrepancy between a sensory measurement and the perceptual estimate for the same sensory modality. Cue reliability, perceptual biases, and the degree to which participants infer that the two cues come from a common source govern audiovisual perception and therefore audiovisual recalibration.

## Introduction

### Cross-modal integration

In our daily lives, we continuously estimate properties of the environment such as the location of a barking dog. Usually multiple sensory cues for each property arrive in the brain, a glimpse of the dog’s wagging tail and the barking both give away its location. However, due to external noise in the environment and internal noise in our sensory systems, two cues hardly ever agree perfectly. To still form a coherent percept, the brain relies on a weighted mixture of the cues. This strategy becomes evident when the cues are in conflict. For example, when a ventriloquist speaks without moving her lips, the auditory signal indicates that the speech originates from the ventriloquist, while the visual signal indicates the “dummy”. Typically, vision dominates the combined spatial estimate of the sound source. In this example, we perceive the “dummy” to be speaking. This phenomenon is called the ventriloquism effect [1–3]. However, when visual reliability, the inverse of the average variability of a cue, is degraded enough to be lower than auditory reliability, the combined spatial estimate is no longer dominated by the visual but instead by the auditory cue, indicating that cue integration depends on their relative reliability [4–6]. This integration strategy maximizes the precision of the estimate, i.e., reduces its variability. In addition to audiovisual spatial perception, reliability-based cue integration has also been found for visual and auditory cues to temporal rate [7–9], visual and haptic size and shape [10–12] as well as numerosity [13], and visual and vestibular cues to heading direction [14–19].

If during a ventriloquist’s show, the speech sounds originate from someone standing behind the stage or if the dummy’s mouth moves out of synch with the speech sounds, the audience is unlikely to experience a ventriloquism effect. In other words, integration breaks down when the two cues are too different to be perceived as coming from a common source. Such a breakdown of integration with spatial and temporal cue conflicts has been found not only in auditory-visual spatial integration [20–25], but also in integration of visual and vestibular heading-direction signals [26, 27] and integration of visual and haptic surface thickness [28]. These findings suggest that the brain infers the causal relationship underlying cues from multiple modalities. A Bayesian observer would not simply decide between integrating the cues or keeping them segregated, but rather combine estimates based on integration and segregation, each weighted by the probability of the underlying causal scenario [29]. This causal-inference model accurately captures the integration of audiovisual spatial signals [29] and has been used to explain cross-modal perception in various contexts [26, 27, 30–40].

### Cross-modal recalibration

Reliability-weighted sensory cue integration maximizes the precision of the final estimate but not its accuracy, the agreement between the cue with the property of interest. Estimates are dominated by the more precise not by the more accurate cue. This preference of precision over accuracy might be rooted in the fact that single sensory cues can contain information about their reliability [41], but information about accuracy is impossible to derive from a cue on its own. Only systematic disparities between two sensory cues indicate a problem with their accuracy and, at the same time, provide a chance to resolve this issue. In the case of mismatched cues, the brain should adapt the interpretation of the cues to reduce that disparity and so, hopefully, maximize accuracy. Indeed, after repeated exposure to spatially discrepant audiovisual stimuli, the shift in auditory spatial perception toward the visual stimulus is still present when the visual signal is no longer available. This persisting shift is called the ventriloquism aftereffect, and has been replicated across different modalities [32, 42–51].

In many spatial-aftereffect studies, vision serves as the “teaching signal” that is used to calibrate auditory or tactile perception. Some studies found a discrepant auditory stimulus presented during adaptation can induce systematic shifts in visual localization, but the aftereffects were not as robust as the recalibration of auditory spatial perception [47, 52, 53]. This dominance of vision seems reasonable given that visual information is usually more accurate and reliable than information from other modalities in terms of localizing objects spatially [54].

#### Reliability-based cross-modal recalibration

Which modality serves as the “teaching signal” in cross-modal recalibration might be determined based on cue reliability. Consistent with this hypothesis, more robust evidence for recalibration of visual perception was found in studies that examined aftereffects for properties for which the visual signal was not as reliable as the other signal [55–60]. Burge and colleagues directly tested the relationship between cue reliability and the amount of recalibration by manipulating the reliability of a visual stimulus for slant to be either smaller, almost equal, or greater than that of a haptic cue to slant [61]. As the visual cue became less reliable, greater recalibration of vision and less recalibration of haptics was observed. The authors concluded, in agreement with others [53, 59], that each sense is recalibrated proportional to its relative reliability.

#### Fixed-ratio cross-modal recalibration

In contradiction to the results just discussed, a recent study investigating the mechanism underlying visual-vestibular recalibration found evidence that the two modalities are recalibrated in a fixed ratio regardless of cue reliability [62]. More specifically, after exposing humans and monkeys to systematically discrepant visual and vestibular heading directions, both cues significantly shifted in the direction required to reduce cue conflict, but the amount of recalibration in either modality did not depend on the measured relative cue reliabilities [62]. A model assuming a fixed ratio between the degree of recalibration of each sense, independent of relative reliability, best captured the data.

#### Causal inference and cross-modal recalibration

Both reliability-based and fixed-ratio models of cross-modal recalibration assume that the brain acts upon the discrepancy between the two sensory cues. However, according to the principles of cross-modal integration, two discrepant cues can lead to very different percepts, depending on each cue’s reliability and inference about whether they have common or separate origins. Cross-modal recalibration might take this perceptual inference into account. Indeed, causal-inference models of cross-modal recalibration have successfully predicted visual-auditory [63] and visual-tactile [32] ventriloquism aftereffects. In these models, recalibration is not based on a mere comparison of two sensory cues, but rather relates the cues to the perceptual estimates and by doing so incorporates causal inference and cue reliability. According to this model, conflicting findings regarding the influence of cue reliability on recalibration might reflect differences in the perceptual estimates rather than diverging underlying mechanisms.

### Preview

In this study, we contrasted all three accounts of cross-modal recalibration by fitting the models to data from an audiovisual ventriloquism-aftereffect study. Across sessions, we manipulated cue reliability, a determinant of reliability-based and causal-inference-driven cross-modal recalibration. Additionally, we controlled for the effect of modality-specific biases on the spatial perception of the two sensory cues by choosing visual stimulus locations that matched perceptually with the locations of the auditory stimuli for each participant.

With decreased visual reliability, many participants showed either increasing or nonlinearly changing auditory recalibration. No clear pattern of visual recalibration was found. These results cannot be explained by either the reliability-based or the fixed-ratio model. The causal-inference model, on the other hand, is able to capture these idiosyncratic effects based on individual differences in cue reliability, modality-specific biases, and an a priori belief about how often visual and auditory cues come from a common source. Thus, the model comparison suggests that cross-modal recalibration is driven by a comparison between sensory cues and perceptual estimates, which in turn are determined using causal inference.

## Results

### Cue reliability

**Fig 1:**
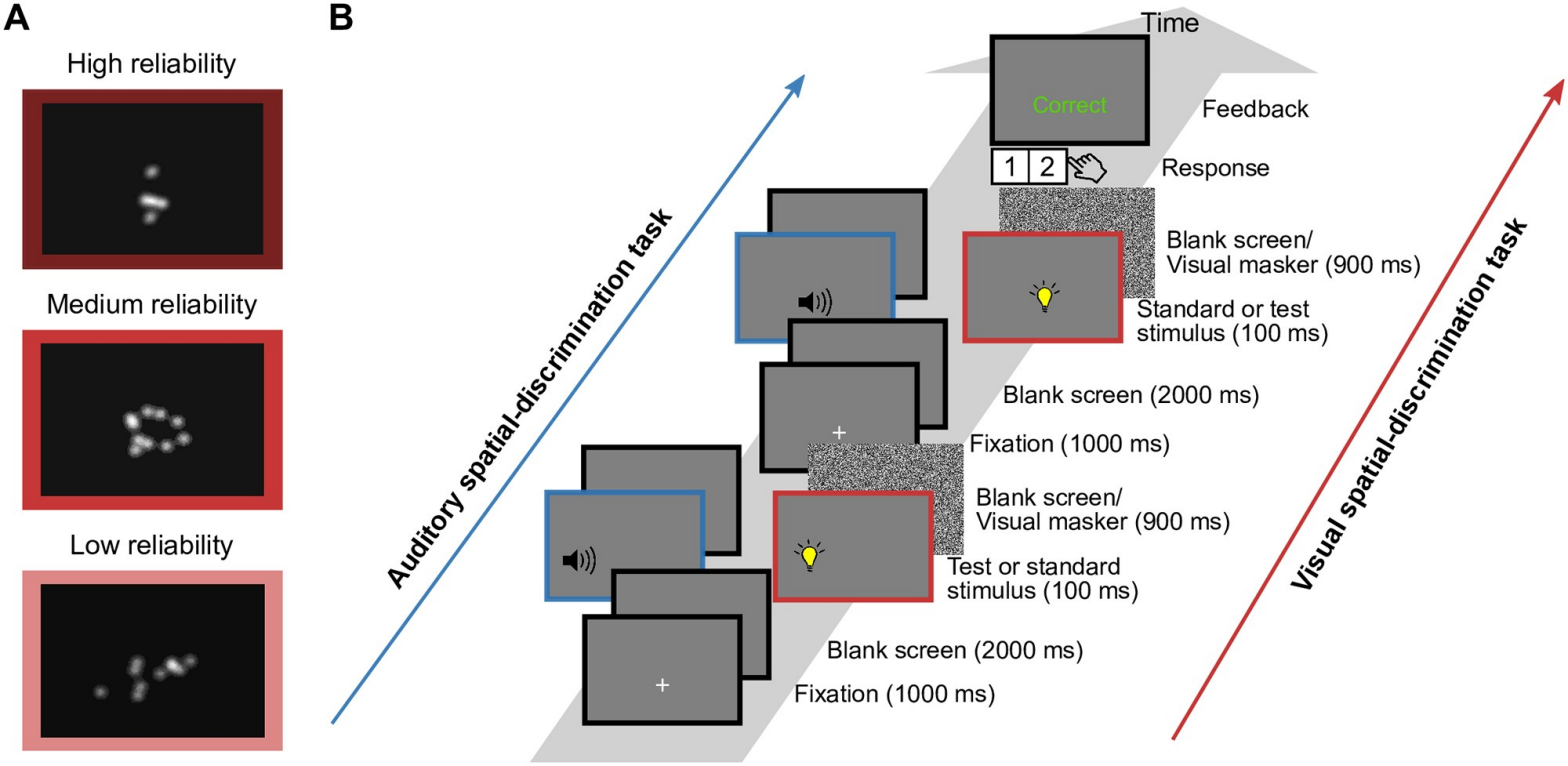
Visual stimuli and experimental procedure for the unimodal spatial-discrimination task. (A) Example visual stimuli (contrast exaggerated). (B) Task timing (blue: auditory stimuli; pink: visual stimuli). Participants were successively presented with a standard stimulus (located straight ahead) and a test stimulus (located to the left or right) in random order. After stimulus presentation, they used a keypad to report which interval contained the stimulus located farther to the right. Feedback was provided.

**Fig 2:**
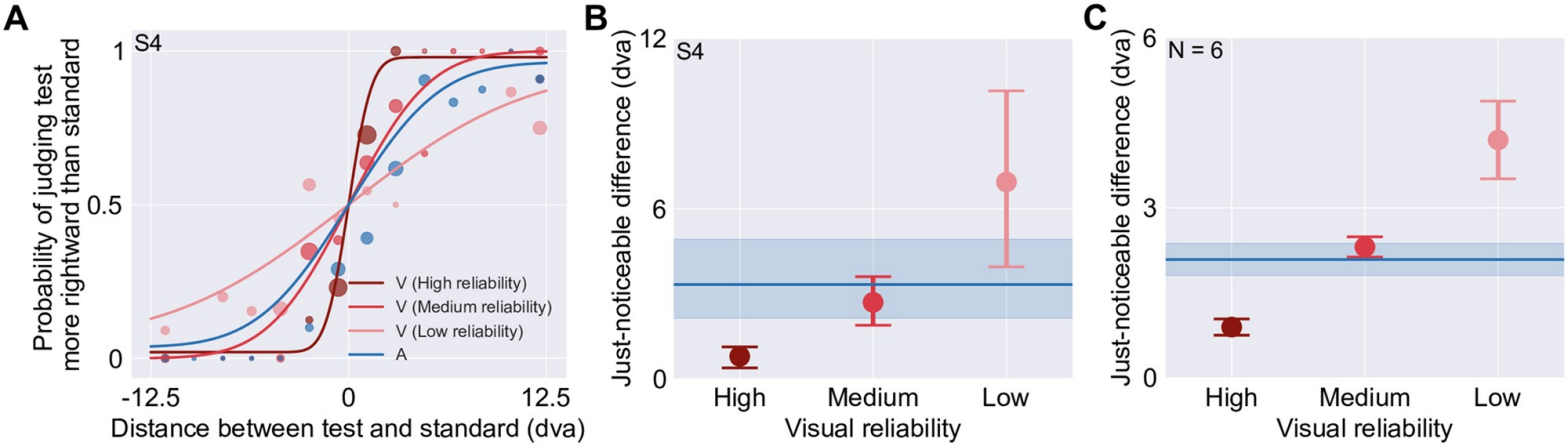
Results for the unimodal spatial-discrimination task. (A) Psychometric functions for representative participant S4. The probability of judging the test stimulus as to the right of the standard stimulus is plotted as a function of the distance between the two stimuli, with negative numbers indicating that the test stimulus was located to the left of the standard stimulus (presented straight ahead). V: Visual. A: Auditory. Filled circles: binned response proportions (bin size = 1.8°). The area of each filled circle is proportional to the number of trials within the bin. Solid curves: psychometric functions fit to the data. (B) Estimated just-noticeable difference (JND) of each visual stimulus type for S4. Solid line: auditory JND. Error bars and blue area: 95% bootstrapped confidence intervals. (C) Group mean JNDs (± SEM).

In the first part of the study, spatial reliability for one auditory stimulus and three visual stimuli was estimated for each participant using a unimodal spatial-discrimination task (Fig 1B; see Materials and methods). Visual reliability had been manipulated by varying the horizontal spread of a random collection of ten Gaussian blobs (Fig 1A). For each stimulus condition, we fitted a cumulative Gaussian distribution to the responses as a function of test stimulus location (Fig 2A, mean adjusted *R*^2^ = 0.953, range = 0.751–0.997). To compare spatial-discrimination performance across stimulus conditions, we computed the just-noticeable difference (JND; Fig 2B and 2C). Statistical analysis of the JNDs (Section S1.1 in S1 Appendix) confirmed that visual stimulus reliabilities were (i) smaller than, (ii) comparable to, and (iii) larger than the auditory reliability.

### Modality-specific spatial biases

**Fig 3:**
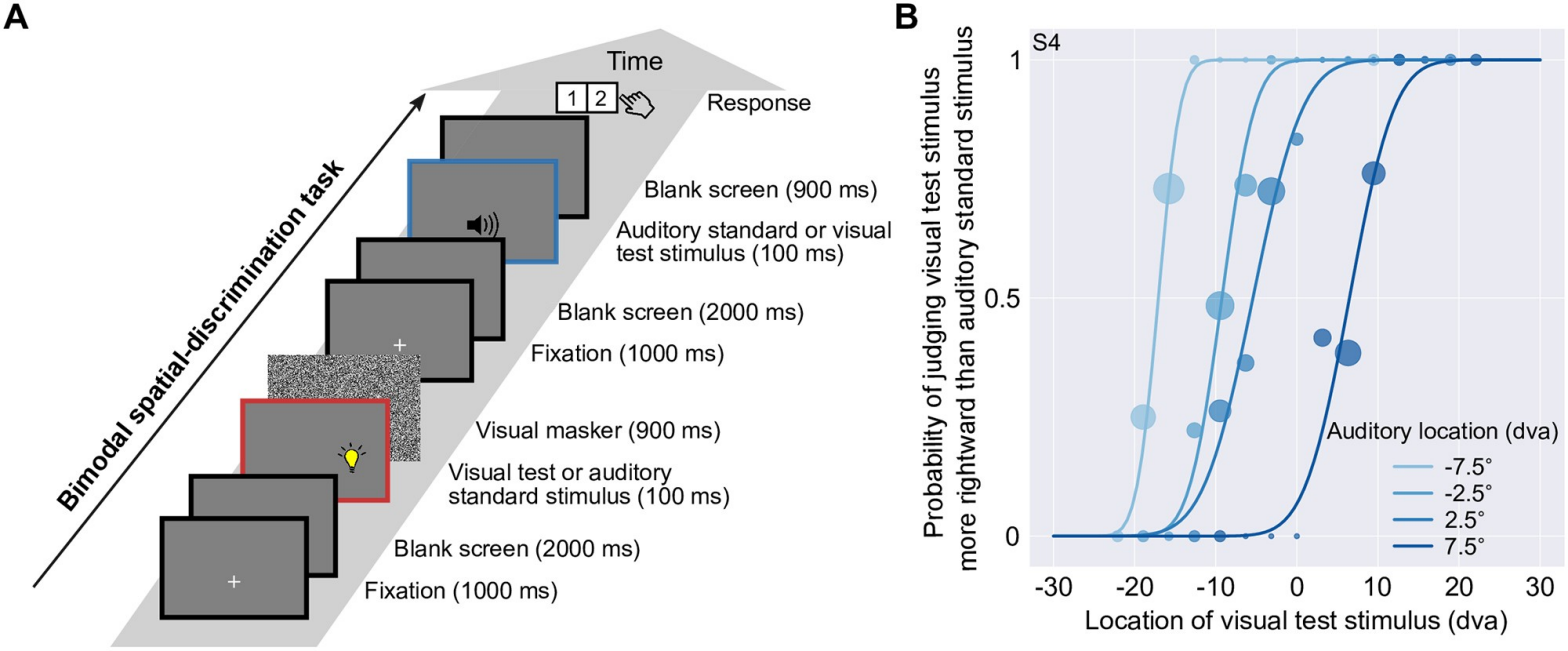
Experimental procedure and results for the bimodal spatial-discrimination task. (A) In each trial, a visual stimulus (high reliability) and an auditory stimulus (four possible auditory locations, ±2.5 and ±7.5° relative to straight-ahead) were presented in random order. Participants reported whether the visual stimulus was to the left or right of the auditory stimulus. Feedback was not provided. (B) Psychometric functions for participant S4. Probability of judging the visual to the right of the auditory stimulus is plotted as a function of visual stimulus location. Filled circles: binned response proportions (bin size = 3°). Curves: psychometric functions fitted separately for the four auditory stimulus locations (shades of blue). The area of each filled circle is proportional to the number of trials in each bin.

**Fig 4:**
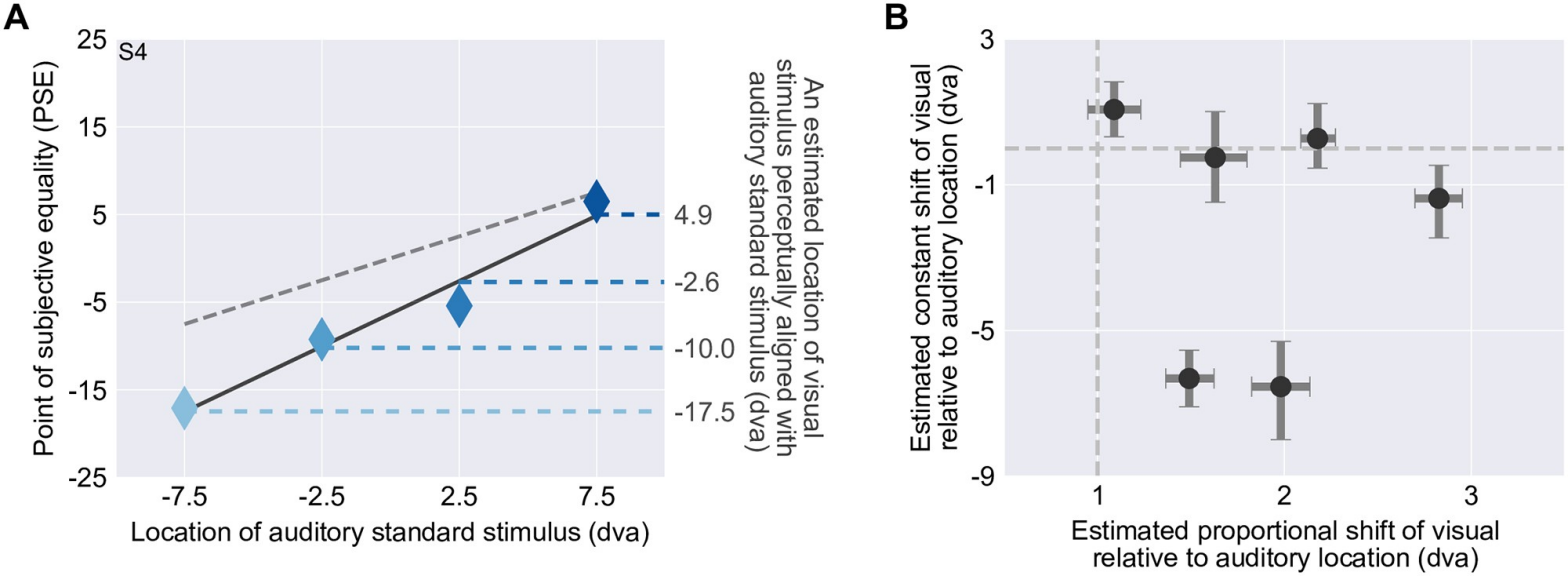
Modality-specific biases in spatial perception. (A) Point of subjective equality (PSE) as a function of the location of the auditory stimulus. Dashed grey line: identity line; solid line: linear regression line; horizontal dashed lines: visual stimulus locations perceived as co-located with the four auditory standard locations based on the regression. (B) Estimated constant, location-independent (*y*-axis; regression intercept) and proportional, location-dependent (*x*-axis; regression slope) shift of visual relative to auditory stimulus location. The proportional and constant shifts equal the slope and intercept of the linear regression line through the PSEs (see panel A). Error bars: 95% bootstrapped confidence intervals. Vertical and horizontal dashed lines correspond to the absence of proportional and constant shifts, respectively.

In the second part of the study, we measured participants’ modality-specific biases in the spatial perception of auditory stimuli relative to a visual stimulus (Fig 1A) using a bimodal spatial-discrimination task (Fig 3A). We did so at four auditory stimulus locations and for visual stimuli with high spatial reliability. Four separate psychometric functions were fitted, one for each auditory stimulus location (Fig 3B, mean adjusted *R*^2^ = 0.909, range = 0.751–0.981). From each psychometric function, we calculated the point of subjective equality (PSE) and then described the four PSEs as a linear function of the underlying auditory location (Fig 4A). The estimated slopes for five out of six participants were significantly larger than 1 (Fig 4B) indicating that the auditory stimuli were perceived as shifted towards the periphery relative to the visual stimuli. Four participants showed significant negative *y*-intercepts. That is, they perceived the auditory stimuli as shifted to the left relative to visual stimuli. From the linear regression line through the PSEs, we extracted the four visual stimulus locations that were perceived to be co-located with the four auditory stimulus locations and used these locations in all subsequent tasks.

### Localization response precision

In the next part of the study, we measured participants’ localization noise unrelated to spatial perception (e.g., noise due to holding a location in memory and errors indicating the intended location). To this aim, participants performed a direct localization task with maximally reliable visual stimuli. At the same time, they were familiarized with our custom-made device used to move a visual cursor to the stimulus location. We assumed that localization errors were unbiased and independent of stimulus location and fitted them with a Gaussian distribution centered at zero to estimate the extent of noise corrupting localization responses (see Section S2 in S1 Appendix for a more complex model and results of a model comparison). Participants’ spatial perception-unrelated localization noise was 1.85° on average (range 1.55–2.29°).

### Recalibration effects

**Fig 5:**
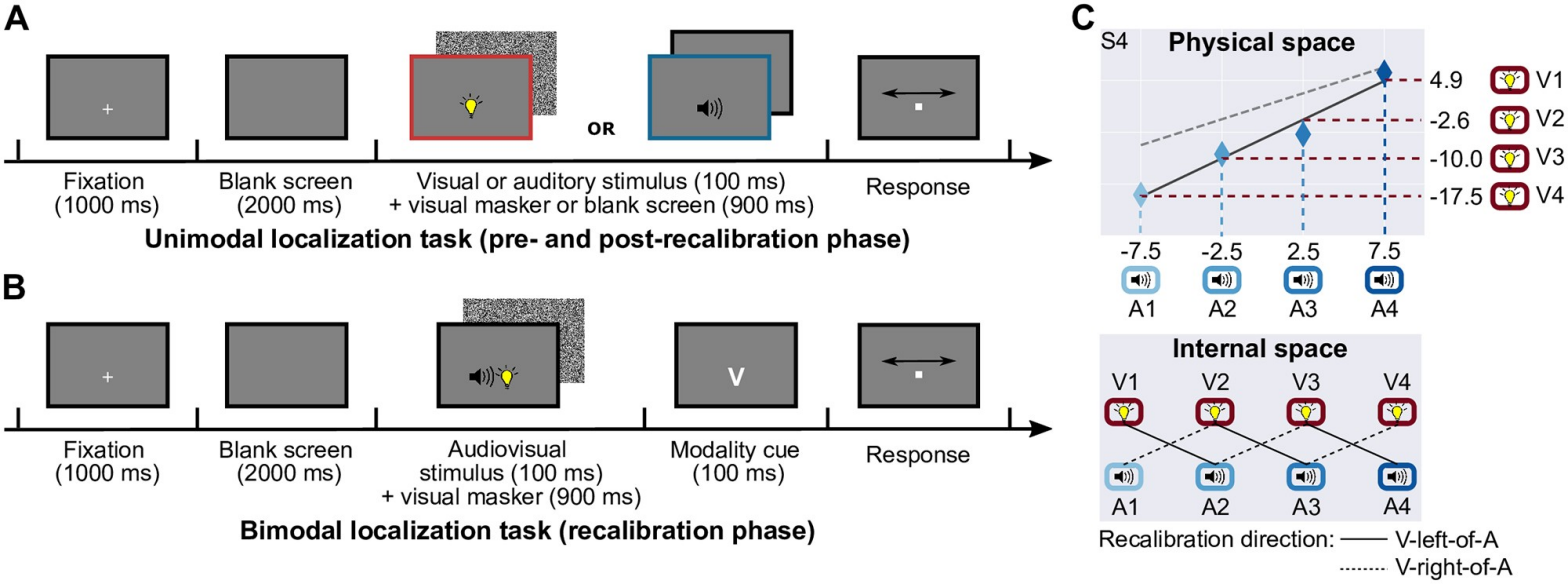
Experimental procedure and conditions during the recalibration experiment. (A) Timeline for unimodal localization tasks (pre- and post-recalibration phase). In each trial, either a visual or an auditory stimulus was presented; participants indicated its location using a visual cursor displayed on the screen. Feedback was not provided. (B) Timeline for the bimodal localization task (recalibration phase). In each trial, participants were presented with a spatially discrepant audiovisual stimulus pair; they were asked to localize one of the modalities, cued after stimulus presentation (V: localize the visual component, A: localize the auditory component). Feedback was not provided. (C) Stimulus locations in physical and perceptual space. Top panel: the physical locations of the four perceptually aligned audiovisual stimulus pairs identified at the beginning of the study for participant S4, stimuli were always presented at one of these locations; bottom row: pairs with a constant perceptual spatial discrepancy were presented during the recalibration phase, solid lines: location pairs presented in the visual-left-of-auditory condition; dashed lines: location pairs presented in the visual-right-of-auditory condition.

In the final part of the study, participants completed six sessions of the audiovisual recalibration experiment (2 recalibration directions × 3 reliability levels). Each session consisted of three phases: (1) pre-recalibration: participants localized unimodally presented visual and auditory stimuli (Fig 5A); (2) recalibration: participants were presented with audiovisual stimulus pairs with a constant spatial discrepancy in perceptual space; they localized one modality cued after stimulus presentation (Fig 5B and 5C); (3) post-recalibration: the unimodal localization task was repeated.

**Fig 6:**
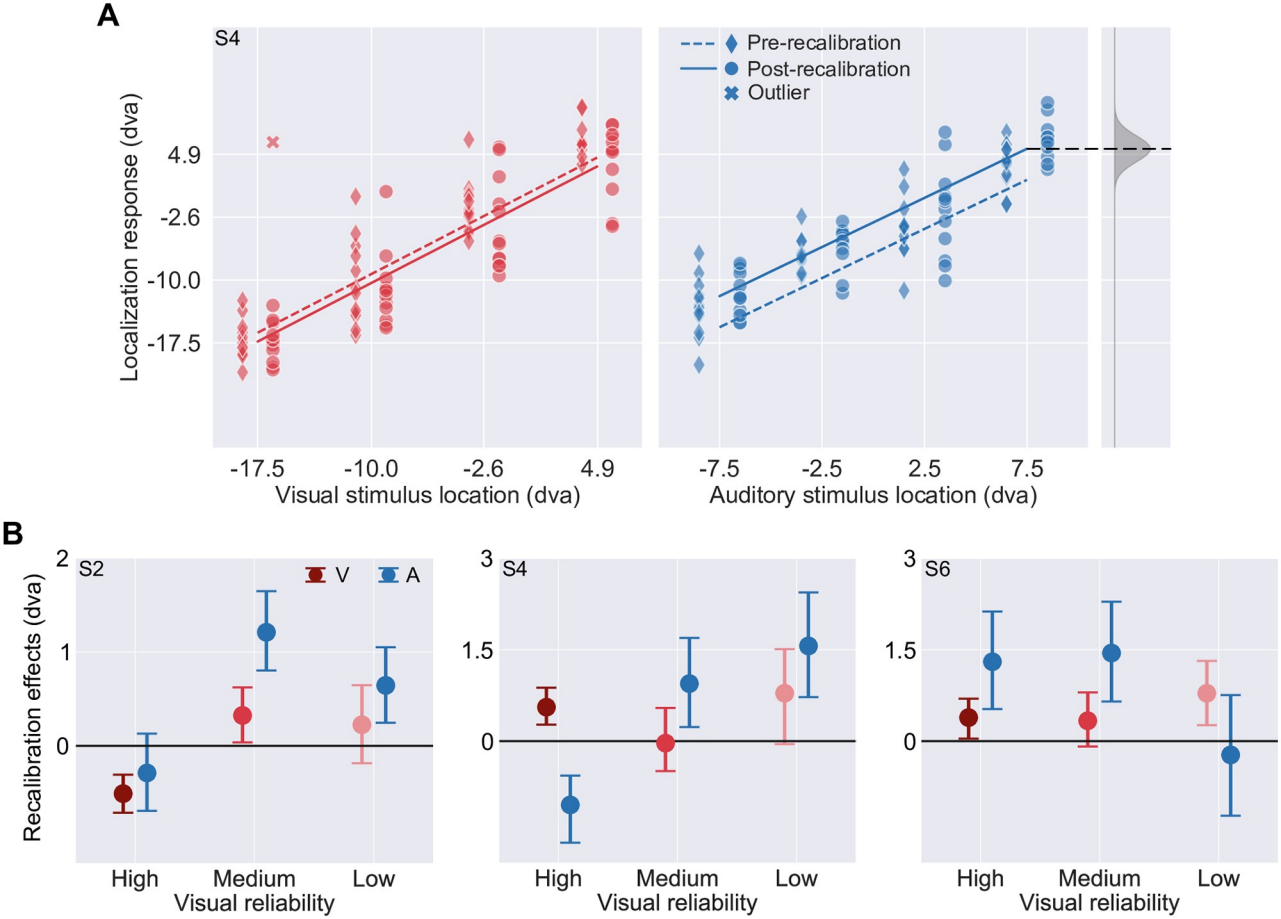
Recalibration effects. (A) Pre- (diamonds, jittered to the left) and post-recalibration (circles, jittered to the right) localization responses as a function of auditory and visual stimulus locations (relative to straight-ahead) measured for participant S4 in the visual-right-of-auditory and low-visual-reliability condition. Localization responses were summarized using linear regression (dashed lines: pre-recalibration; solid lines: post-recalibration). Grey shaded area: estimated probability distribution of spatial perception-unrelated localization noise centered at an example location. (B) Auditory and visual recalibration effects (the difference between the intercepts of the pre-and post-recalibration regression lines in panel A) as a function of visual stimulus reliability for three participants. Error bars: 95% bootstrapped confidence intervals.

To statistically examine recalibration of each modality, we fitted linear regressions separately to pre- and post-recalibration localization responses as a function of stimulus location (Fig 6A). Recalibration effects were computed as the difference between pre- and post-recalibration regression intercepts; shifts compensating for the audiovisual discrepancy present during the recalibration phase were coded as positive. As no significant effects of recalibration direction on the recalibration effect were found in our statistical analysis (Section S1.2 in S1 Appendix), we averaged the recalibration effect across the two recalibration directions (visual to right/left of auditory) for display (Fig 6B). For the majority of participants (three out of six), auditory recalibration was a non-monotonic function of visual stimulus reliability. Two participants showed a monotonic increase in auditory recalibration with decreasing visual stimulus reliability. One participant showed a monotonic decrease in auditory recalibration as visual reliability decreased. Unsurprisingly, our statistical analysis of the recalibration effect revealed no significant main effects or interactions involving visual stimulus reliability (Section S1.2 in S1 Appendix).

### Recalibration models

To understand the mechanisms of cross-modal recalibration, we compared participants’ behavior to three existing models of cross-modal recalibration: (1) a reliability-based, (2) a fixed-ratio, and (3) a causal-inference model (for details see Models of audiovisual recalibration). All three models conceptualize recalibration as constant updating of a modality-specific shift applied to the measurements before the estimate is derived. However, these three models differ in their assumptions about the way in which the amount of recalibration for each modality is determined. As a consequence, these three models make different predictions for the influence of visual reliability on audiovisual recalibration.

#### The reliability-based model

**Fig 7:**
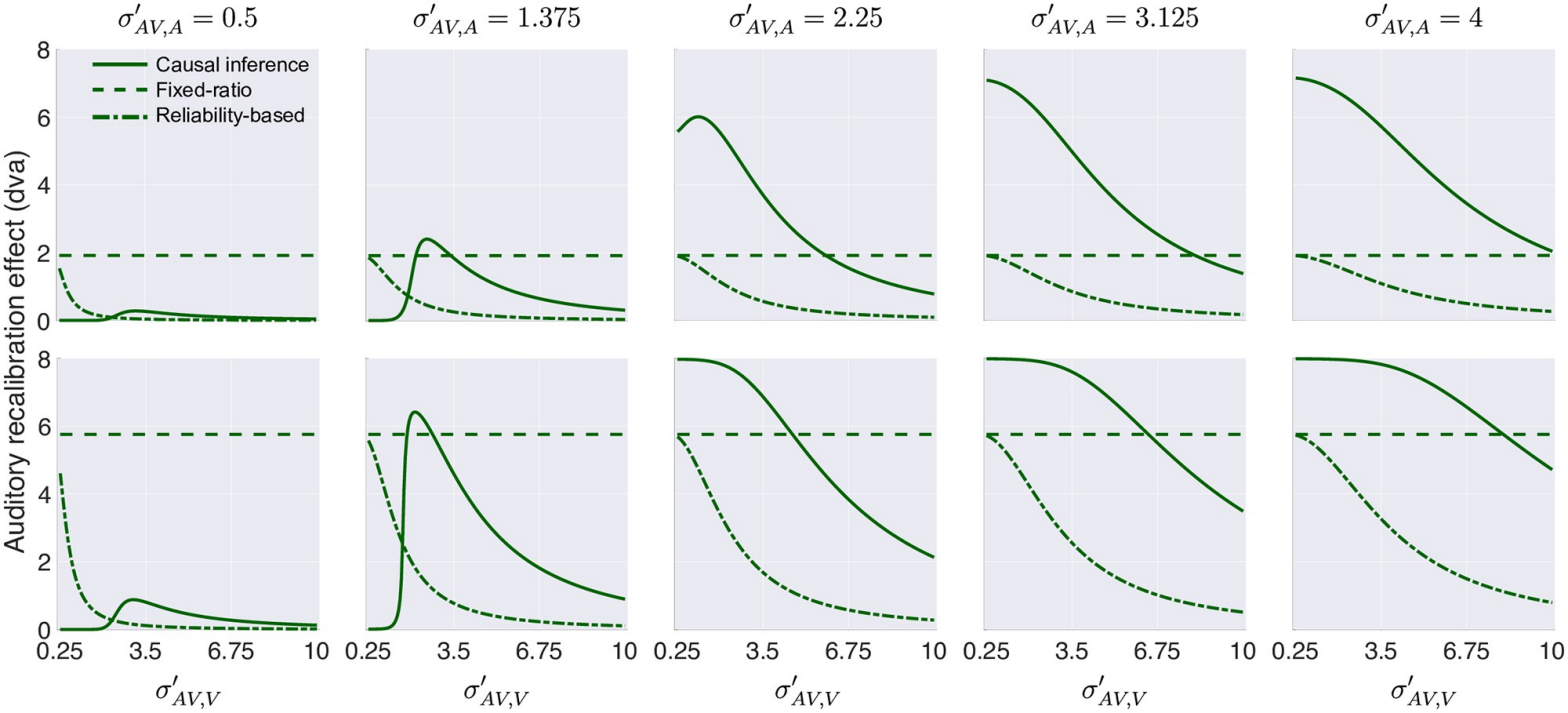
Simulated auditory recalibration effects based on three candidate models of cross-modal recalibration. The effect of visual (horizontal axis) and auditory (panels) stimulus reliability in bimodal trials on the amount of auditory recalibration by vision, based on causal-inference (solid line), fixed-ratio (dashed line), and reliability-based (dot-dashed line) models for two different learning rates (top row: slow; bottom row: fast).

According to this model, the brain determines the amount of recalibration (i.e., the measurement shift) for each modality based on the reliabilities of both measurements. The shift for one modality is updated by a fraction of the difference between the two measurements that is proportional to the other modality’s relative reliability. Across trials, the amount of recalibration depends not only on stimulus reliability, but also on a common learning rate *α* for both modalities. This model predicts increasing visual and decreasing auditory recalibration effects with decreasing visual stimulus reliability (Fig 7, dot-dashed line).

#### The fixed-ratio model

According to this model, the brain determines the amount of recalibration based on the modalities of both sensory measurements. The measurement shift for each sense is updated by a fraction of the difference between the two measurements. This fraction is determined only by modality-specific learning rates. Therefore, this model predicts modality-specific recalibration effects that are not influenced by stimulus reliability (Fig 7, dashed line).

#### The causal-inference model

According to this model, the brain determines the amount of recalibration for each modality based on the difference between a measurement and the corresponding location estimate. The measurement shifts are updated by a fraction of this difference, either implemented as modality-specific learning rates or as a supra-modal learning rate. In this model, cross-modal recalibration depends on stimulus reliability, modality-specific localization biases, and inference about a common cause through their influences on the location estimates. The model can predict various effects of visual reliability on auditory recalibration (Fig 7, solid line).

### Modeling results

#### Model predictions

**Fig 8:**
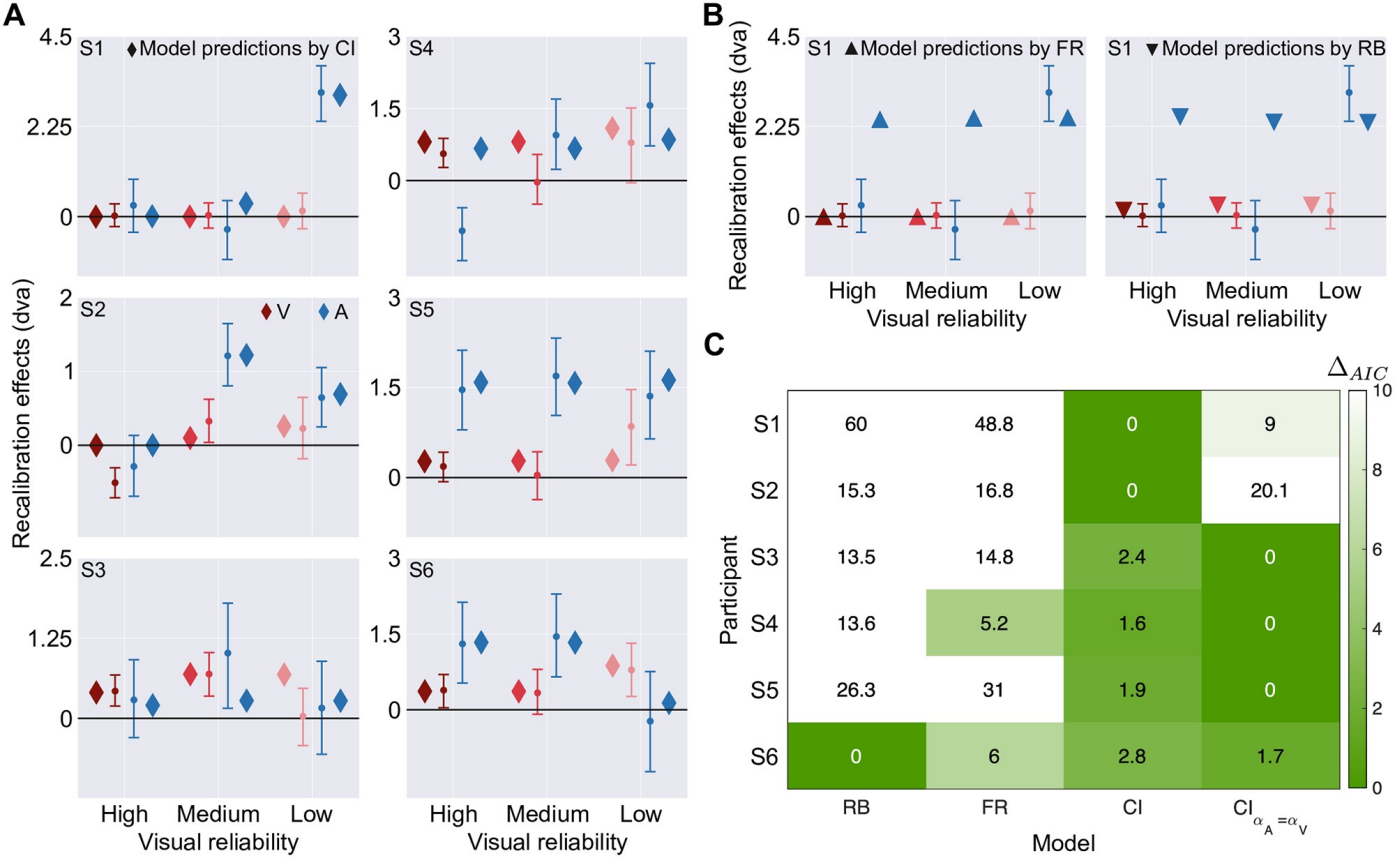
Model predictions and model comparison. (A) Observed (circles) and predicted (diamonds) auditory (blue) and visual (pink) final measurement shifts after the recalibration phase based on 1,000 runs using the best-fitting parameters given the causal-inference model (diamonds) as a function of visual reliability for all participants (panels). Error bars: 95% bootstrapped confidence intervals. (B) Model predictions by the fixed-ratio model (triangles in left panel) and the reliability-based model (inverted triangles in right panel) for participant S1. (C) Model comparison indices, smaller values indicate more evidence (RB = reliability-based, FR = fixed ratio, CI = causal inference, ${\text{CI}}_{\alpha_{A}=\alpha_{V}}$ = causal inference with supramodal learning rate).

The causal-inference model is the only model that can capture the idiosyncratic influence of visual stimulus reliability on auditory recalibration across participants (Fig 8A). Data from 5 out of 6 participants were best fitted by the causal-inference model, either with a supra-modal learning rate (see Section S3 in S1 Appendix for model predictions) or modality-specific ones. For most participants, neither the reliability-based nor the fixed-ratio model can reproduce the diverse influence of visual stimulus reliability on auditory recalibration (Fig 8B; Sections S4-S5 in S1 Appendix). The three models do not differ in terms of predicting visual recalibration and modality-specific biases (Section S6 in S1 Appendix).

#### Model comparison

To compare model performance quantitatively, we computed the Akaike information criterion (AIC) for all three models [64] and then calculated relative model-comparison scores, Δ_AIC_, which relate the AIC value of the best-fitting model (the model with the lowest AIC) to that of each of the other models (a high value of Δ_AIC_ indicates stronger evidence for the best-fitting model; Fig 8C). Δ_AIC_ values comparing both the reliability-based and the fixed-ratio to the causal-inference models were large for the majority of participants, revealing substantial evidence for the causal-inference model of cross-modal recalibration.

## Discussion

In this study, we investigated the mechanism underlying cross-modal recalibration. To this aim, we measured the effects of visual stimulus reliability, a potential determinant of cross-modal recalibration, on audiovisual spatial recalibration. To induce recalibration, we repeatedly exposed participants to audiovisual pairs with a perceptually constant spatial discrepancy. To measure recalibration, we compared unimodal auditory and visual localization responses before and after the exposure. Auditory localization was recalibrated by vision, yet, the influence of visual stimulus reliability on auditory recalibration differed qualitatively across participants. To scrutinize the mechanisms of cross-modal recalibration, we compared participants’ behavior to three models of recalibration: (1) a reliability-based model, which assumes that the amount of recalibration depends on the relative reliability of the cues that are in conflict, (2) a fixed-ratio model, which assumes that the amount of recalibration is fixed, dependent only on the modalities in conflict and independent of cue reliability, and (3) a causal-inference model, which ties recalibration to the percept of a cue. This percept depends on the other cue, causal inference of the two cues coming from a common source as well as cue reliabilities, and modality-specific spatial biases. Only the causal-inference model captured the idiosyncratic influences of cue reliability on cross-modal recalibration.

### Only the causal-inference model captures the diverse influences of visual reliability on auditory recalibration by vision

Our results demonstrated diverse influences of visual stimulus reliability on auditory recalibration. For half of the participants, auditory recalibration was maximal at medium visual stimulus reliability. For some other participants, auditory recalibration increased with decreasing visual stimulus reliability. Neither of these patterns can be replicated by models of recalibration that assume the amount of recalibration relies directly on cue reliability [53, 61]. These models can only predict decreases in recalibration as the stimulus reliability of the other modality decreases, as has been found previously [59, 61]. Thus, the best prediction these models could produce for either monotonically or non-monotonically increasing auditory recalibration effects with decreasing visual reliability was no influence of stimulus reliability (Fig 8B, right panel). The observed influences of cue reliability on recalibration are also at odds with models of recalibration that assume the amount of recalibration relies only on the identity of the two modalities in conflict [62, 65]. These models predict no influence of stimulus reliability on recalibration. Crucially, the causal-inference model of cross-modal recalibration [32, 63] captures all the observed influences of stimulus reliability on cross-modal recalibration and is able to replicate all previous patterns of results [61, 62] based on individual differences in cue reliability, the common-cause prior, and modality-specific spatial biases.

It is remarkable that the causal-inference model of cross-modal recalibration is capable of producing qualitatively different patterns of results for the amount of cross-modal recalibration as stimulus reliability changes [32]. Based on our experience with the model, we next provide an intuition of how individual differences in the sensory reliabilities, biases in spatial perception of both modalities, and the common-cause prior influence cross-modal recalibration according to the causal-inference model.

#### The role of cue reliability for cross-modal recalibration

**Fig 9:**
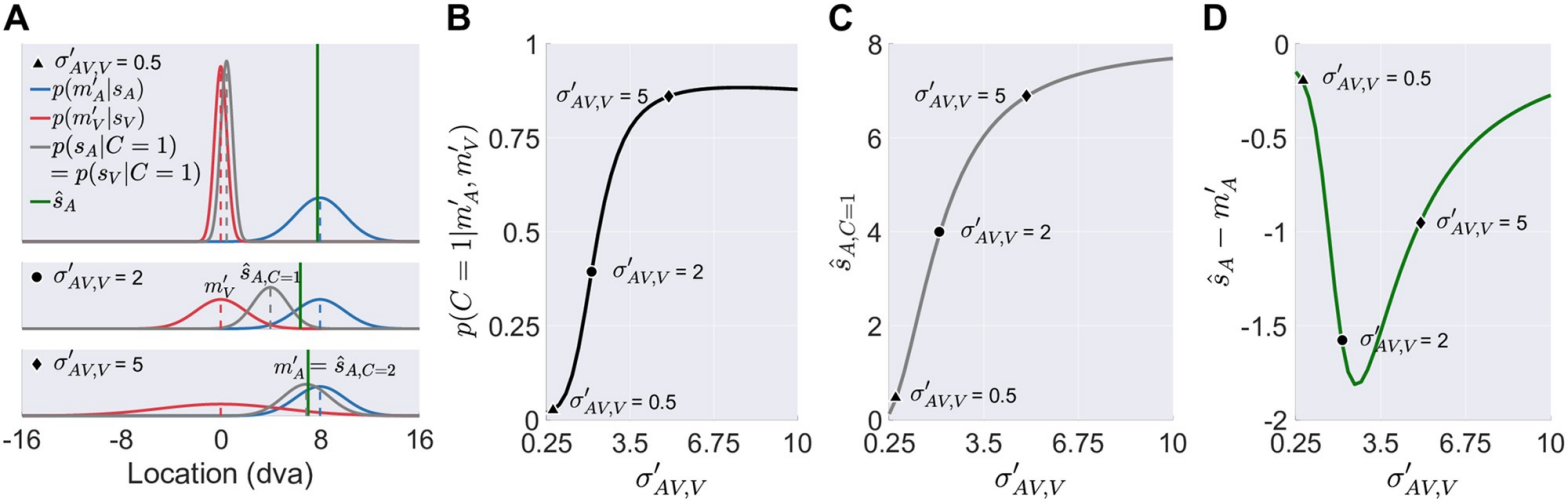
Cue reliability and the amount of recalibration. (A) Non-linear effect of visual stimulus reliability in bimodal trials ($\sigma_{AV,V}^{\prime }$; different panels) on the auditory spatial estimate ${\hat{s}}_{A}$ (green vertical lines). Blue and red dashed vertical lines and curves: auditory and visual measurements ($m_{A}^{\prime }$ and $m_{V}^{\prime }$ in perceptual space) and likelihood functions, respectively. Blue: when separate causes are assumed, the auditory measurement and likelihood equal the auditory location estimate ${\hat{s}}_{A,C=2}$ and the posterior distribution over auditory locations (a flat prior over stimulus location is assumed). Grey dashed vertical lines and curves: audiovisual location estimates ${\hat{s}}_{A,C=1}$ and posterior distributions of audiovisual locations conditioned on a common cause. (B-D) The effect of visual reliability on the posterior probability of a common cause, $p(C=1|m_{A}^{\prime },m_{V}^{\prime })$, the integrated location estimate, i.e., the estimate conditioned on a common audiovisual source ${\hat{s}}_{A,C=1}$, and the distance between auditory measurement and location estimate ${\hat{s}}_{A}-m_{A}^{\prime }$, which directly sets the amount of recalibration.

Cue reliability influences the degree of cross-modal recalibration by influencing the posterior probability that both cues arose from a common cause as well as the integrated location estimate for the common-cause scenario. Both of them determine the final location estimate and in turn the amount of recalibration. When the visual cue is extremely reliable, the posterior probability that two discrepant cues originated from the same source is low (Fig 9B). If the common-cause scenario is unlikely, the auditory location estimate is mostly based on the estimate given separate sources and therefore located close to the auditory cue (Fig 9A, top panel), which leads to small recalibration effects.

However, the amount of auditory recalibration does not increase monotonically with decreasing visual reliability. With decreasing visual reliability, the integrated location estimate for the common-cause scenario is increasingly dominated by the auditory measurement (Fig 9C). The closer the integrated estimate is to the auditory measurement, the closer the final location estimate is to the auditory measurement, and the smaller the auditory recalibration effect will be.

Importantly, the effect of variation in reliability depends on the tested reliability range (Fig 9D). Thus, studies that use different types of stimuli will likely obtain different results regarding the influence of stimulus reliability on cross-modal recalibration. The contradictions that emerged between previous studies [61, 62] can be explained by the causal-inference model of recalibration.

#### The role of common cause prior assumptions for cross-modal recalibration

**Fig 10:**
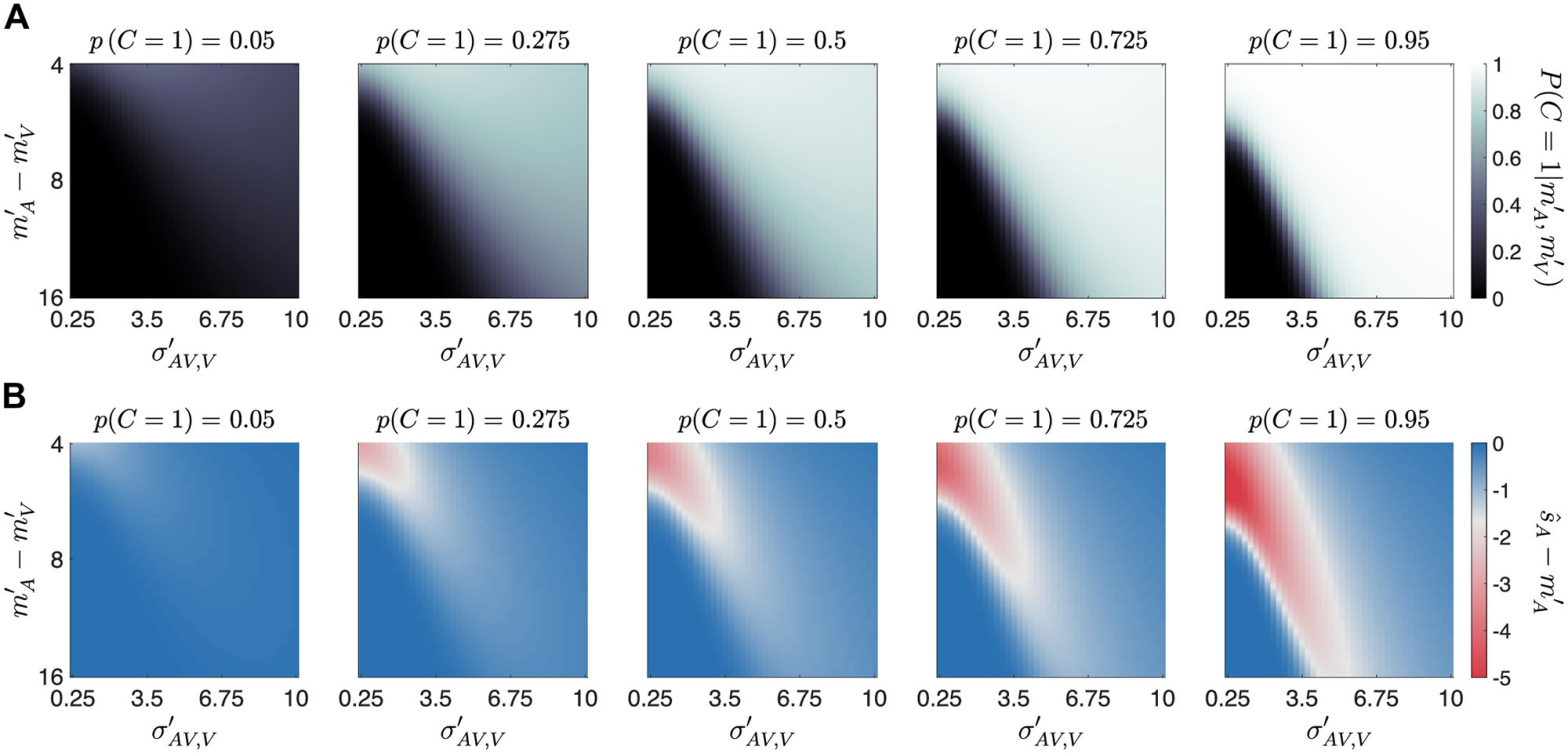
Determinants of recalibration. The joint effects of visual reliability in bimodal trials, $\sigma_{AV,V}^{\prime }$, the distance between auditory and visual measurements in perceptual space, $m_{A}^{\prime }-m_{V}^{\prime }$, and the common-cause prior, *p*(*C* = 1) on the posterior probability of a common cause, $p(C=1|m_{A}^{\prime },m_{V}^{\prime })$ (panel A), and the distance between the final location estimate and the auditory measurement, ${\hat{s}}_{A}-m_{A}^{\prime }$ (panel B), which is proportional to the amount of recalibration.

The common-cause prior impacts the posterior probability of a common cause and hence the amount of recalibration independent of stimulus reliability and discrepancy (Fig 10). As the common-cause prior increases, the posterior probability of a common cause increases, leading the final auditory location estimate to be farther away from the auditory measurement and hence yielding a larger measurement-shift update. We used a post-response cue in the bimodal localization task during the recalibration phase to foster audiovisual recalibration as the common-cause prior is strengthened when participants attend to both modalities [32].

#### Modality-specific spatial biases

Our model differs from previous causal-inference models of cross-modal perception in that we assumed the existence of modality-specific biases but not modality-specific spatial priors over stimulus location [32, 42, 66]. Both can account for the typically observed biases in localization, but modality-specific priors exert a stronger influence when visual reliability is reduced. In contrast, the influence of modality-specific biases does not vary as stimulus reliability changes. Thus, we conducted a control experiment examining whether the tendency to perceive visual stimuli as shifted towards the central fixation increased with decreasing visual reliability (Section S7 in S1 Appendix). The results showed no systematic influence of visual reliability. Thus, to avoid overfitting the model, we omitted modality-specific priors in our models and fitted only modality-specific biases.

The existence of fixed biases in the spatial perception of visual and auditory stimuli seems to be at odds with the concept of cross-modal recalibration. If discrepancies between the senses consistently lead to recalibration, why do these biases persist? Perceptual biases could reflect adjustments for sensory discrepancies during an early sensitive period of development as has recently been shown for cross-modal biases in temporal perception [31]. After the sensitive period, it might become impossible to fully compensate for newly developing differences between the senses. In terms of our model, this would mean that a limitation to the shift-updates is set during early infancy.

**Fig 11:**
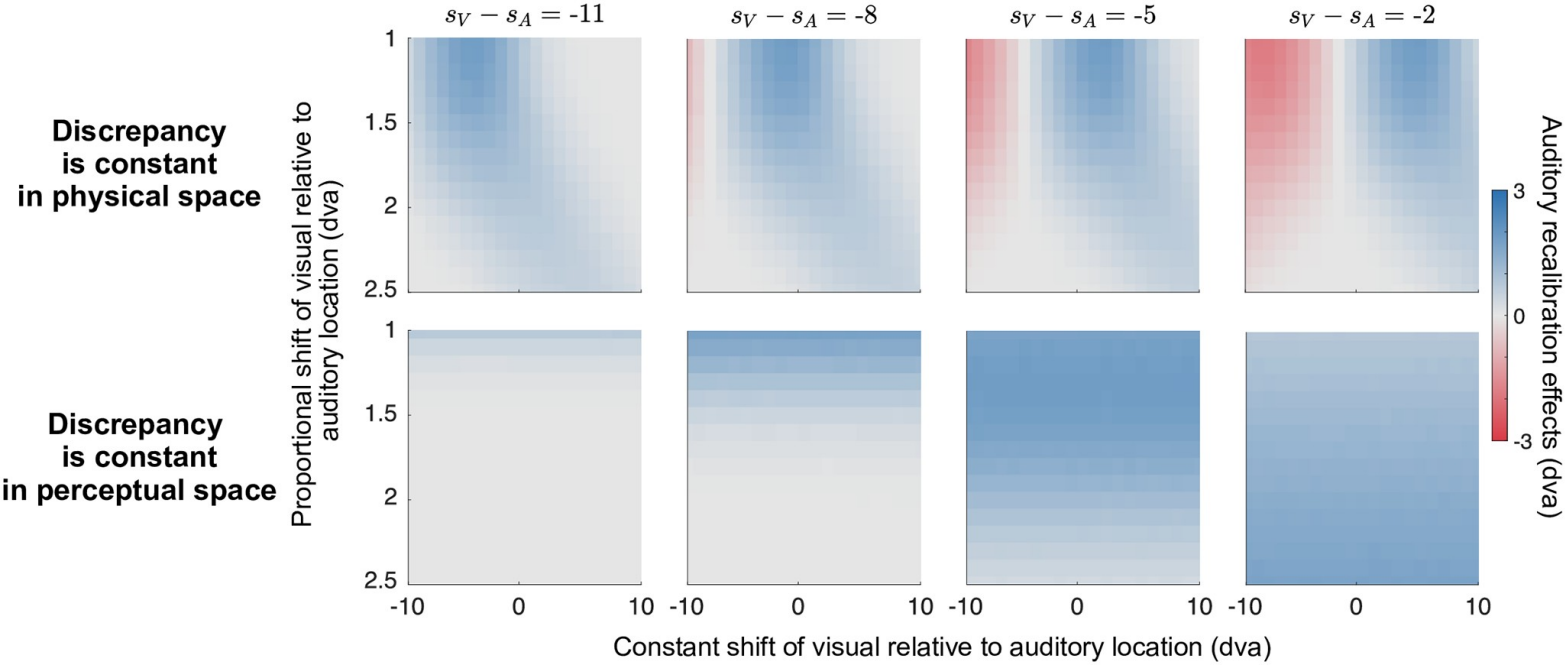
The influence of spatial discrepancy and modality-specific biases on the amount of auditory recalibration. Top row: The auditory recalibration effects (color key) as a function of proportional and constant shift of visual relative to auditory location when spatial discrepancy (panels) is constant in physical space (i.e., *s*_*V*_ = *s*_*A*_ + spatial discrepancy). Bottom row: The auditory recalibration effects when spatial discrepancy is constant in perceptual space (i.e., visual stimulus locations are selected to adjust the perceptual biases in auditory relative to visual spatial perception, *s*_*V*_ = proportional shift × (*s*_*A*_ + spatialdiscrepancy) + constant shift).

Our study differs from previous spatial-recalibration studies in that we adjusted for perceptual biases in auditory relative to visual spatial perception by selecting visual locations perceptually aligned with pre-selected auditory locations. During piloting we presented stimuli with a constant physical spatial audiovisual discrepancy during the recalibration phase, and did not find significant recalibration effects. We attributed this to the modality-specific biases we had observed combined with the small spatial discrepancy used in our study. Indeed, our simulations with the causal-inference model (Fig 11, top panel) show that there are many combinations of proportional and constant biases that lead to minuscule or even negative recalibration effects when these biases are not adjusted for. Additionally, our simulations reveal a complex interaction between these biases and the spatial discrepancy. Recalibration through exposure to a constant and relatively large physical discrepancy, as done in most previous studies [43–45, 47–50, 67], would produce positive recalibration effects on average but not necessarily in all participants given that humans differ in their spatial biases. In contrast, keeping the discrepancy constant in perceptual space makes the recalibration effects less prone to individual differences in modality-specific biases and works better with smaller spatial discrepancies (Fig 11, bottom panel).

### Fitting the causal-inference model

Here, we fitted, for the first time, the causal-inference model of recalibration to observed data. To achieve this, we fitted the outcome of the recalibration process rather than its build-up. The audiovisual recalibration phase itself is characterized by sequential dependence of the measurement shifts across trials and the lack of a closed-form solution for the model. Fitting such models is computationally expensive as the required number of simulations increases exponentially with each recalibration trial due to the sequential nature of the model. There might be a way to address these sequential dependencies using particle filters [68]. However, we concentrated on fitting the outcome of the recalibration process by repeatedly simulating the audiovisual recalibration phase. In this way, we obtained an approximation of the distribution of measurement-shifts given a set of parameters. The good match between the observed and predicted unimodal localization data as well as our checks of the fitting procedure (Section S8 in S1 Appendix) confirm the validity of our approach. One negative consequence of not fitting the recalibration process itself is that parameters reflecting stimulus reliability under bimodal conditions are not directly constrained by the data. We incorporated different reliabilities for unimodal and bimodal presentation conditions because previous studies indicated differences in one [32, 37, 69] or the other [70, 71] direction. Yet, we remain cautious in interpreting the estimated bimodal reliabilities in our study.

We additionally remain cautious with respect to the interpretation of other parameter estimates. We assumed a flat supra-modal prior over stimulus location as we found indications for trade-offs between modality-specific biases and a supra-modal prior over stimulus locations (Section S9.1 in S1 Appendix). As a consequence, the estimated modality-specific biases might have been underestimated and sensory reliabilities might have been overestimated. Additionally, the prior probability of a common cause and the modality-specific learning rate trade off and thus might be misestimated, because an increase in either factor can lead to a greater amount of recalibration (Section S9.2 in S1 Appendix).

Importantly, even though the possibility of biases in our parameter estimates exists, the mechanisms outlined at the beginning of the discussion explain the idiosyncratic influence of visual reliability on auditory recalibration, whereas the other models cannot qualitatively reproduce the observed results. Thus, our conclusion that causal-inference-based percepts regulate cross-modal recalibration stands independent of the parameter estimates.

Future work might involve an experimental design that allows for better estimation of model parameters to enable a determination of the underlying cause of these individual differences and to relate them to behavior in other tasks. For example, more constraints on the experimental parameters could be obtained in a design in which unimodal trials are interspersed with recalibration trials, allowing the time course of recalibration to be measured.

### Conclusion

This study examined the mechanism underlying cross-modal recalibration. To this aim, we measured audiovisual spatial recalibration while varying visual stimulus reliability. Stimulus reliability has been described as one plausible determinant the brain uses to decide which sensory modality should be recalibrated when there is a cue conflict. We found that visual stimulus reliability influenced auditory recalibration in qualitatively different ways across participants. Neither the reliability-based model nor an alternative model that assumes a fixed degree of recalibration for each modality and completely ignores stimulus reliability, could replicate the data. Yet, a causal-inference model was able to capture all the observed diverse influences of reliability on recalibration, including two patterns found in previous studies. In this model, recalibration is not based on a mere comparison of two sensory cues, but rather relates each cue to its corresponding perceptual estimate, and by doing so incorporates causal inference of a common source for a cross-modal stimulus pair as well as cue reliability and modality-specific perceptual biases into cross-modal recalibration.

## Materials and methods

### Ethics statement

Experimental protocols were approved by the Institutional Review Board at New York University (protocol number FY2016–595). All participants gave informed written consent prior to the beginning of the experiment and five of them were compensated $10 per hour for participation.

### Participants

Six participants (three females, aged 22–29 years, mean: 25 years, six right-handed), recruited from New York University and naive to the purpose of the study, participated in the experiment. All stated that they were free of visual, auditory, or motor impairments. The data of one additional participant (female, 21 years, ambidextrous) were excluded from data analysis and model fitting due to conflicting modality-specific spatial biases found in the bimodal spatial-discrimination and unimodal localization tasks (Section S10 in S1 Appendix).

### Apparatus and stimuli

The experiment was conducted in a dark and semi sound-attenuated room. Participants were seated 1 m from an acoustically transparent, white screen (1.36 × 1.02 m, 68 × 52° visual angle). An LCD projector (Hitachi CP-X3010N, 1024 × 768 pixels, 60 Hz) was mounted above and behind participants to project visual stimuli on the screen. The visual stimuli were clusters of 10 randomly placed low-contrast (36.55 cd/m^2^) Gaussian blobs (SD: 3.6°) added to a grey background (29.33 cd/m^2^). Blob locations were drawn from a two-dimensional Gaussian distribution (vertical SD: *σ*_*y*_ = 5.4°, horizontal SD: (1) *σ*_*x*_ = 1.1° for the high-reliability condition, (2) *σ*_*x*_ = 5.4° for the medium-reliability condition, and (3) *σ*_*x*_ = 8.7° for the low-reliability condition). We recorded the centroid of the cluster rather than the center parameter of the two-dimensional Gaussian used for cluster generation as the visual stimulus location. Each visual stimulus was presented for 100 ms (6 frames), and followed by a 900 ms long backward masker (54 frames of randomly black or white checks of 4 × 4 pixels filling the screen) to erase any visual memory of the stimulus.

Behind the screen, a loudspeaker (20 W, 4Ω Full-Range Speaker, Adafruit, New York) was mounted on a sledge attached to a linear rail (1.5 m long, 23 cm above the table, 5 cm behind the screen). The rail was hung from the ceiling using elastic ropes, perpendicular to the line of sight. The position of the sledge on the rail was controlled by a microcomputer (Arduino Mega 2560; Arduino, Somerville, MA, USA). The microcomputer controlled a stepper motor that rotated a threaded rod (OpenBuilds, www.openbuildspartstore.com). This way the speaker was moved to the auditory stimulus location. The auditory stimulus was a 100 ms broadband noise burst (0–20.05 kHz, 60 dB), windowed using the first 100 ms of a sine wave with a period of 200 ms. To control audiovisual synchrony in bimodal trials, we adjusted audiovisual latencies in the presentation software and confirmed their synchrony by recording their relative latencies using a microphone and photo-diode.

We were concerned that participants might infer the position of the speaker from the sounds produced by sledge movements. We tried to foil this strategy by playing a masking sound from an additional speaker behind the center of the screen during each movement of the speaker. The masking sound (55 dB) was a recording of a randomly chosen speaker movement plus white noise. Additionally, the speaker moved from its last position to the target location through a stopover location. The stopover was randomly chosen under the constraint that the total distances the speaker moved were approximately equal across trials. We carried out a control experiment, which indicated that participants could not infer the speaker position based on sounds arising from the speaker movements (Section S11 in S1 Appendix).

Responses were given using a numeric keypad in spatial-discrimination tasks, and a pointing device in localization tasks. The pointing device was custom built using a potentiometer (Uxcell 10KΩ Linear Taper Rotary Potentiometer) with a plastic ruler (5 × 17 cm) securely fixed perpendicular to the shaft of the potentiometer. Participants placed their hands on either side of the ruler and rotated it so that it pointed at the perceived location of the stimulus. A visual cursor (an 8 × 8 pixel white square) was displayed to indicate the selected location. The pointing device was covered by a black box (42 × 30 × 15 cm) so that participants could not see their hands while using the device. A foot pedal was placed on the floor and used to confirm the current position of the pointing device as the response.

Stimulus presentation, speaker movement, and response collection were controlled by a laptop PC running MATLAB R2017b (MathWorks, Natick, MA, USA). Visual stimuli were presented using the Psychophysics Toolbox [72–74].

### Procedure

In the beginning of the study, participants completed a unimodal spatial-discrimination task, once for the auditory stimulus, and once for each of the three visual stimuli (low, medium, and high spatial uncertainty). Participants then completed a bimodal spatial-discrimination task followed by a pointing practice task. The last part of the study consisted of six recalibration sessions, one for each condition (2 recalibration directions × 3 reliability levels). Each session started with a unimodal localization task, followed by an audiovisual recalibration phase in which participants localized one modality of a spatially discrepant audiovisual stimulus, and ended with a repetition of the unimodal localization task.

#### Unimodal spatial-discrimination task

Participants’ spatial-discrimination thresholds were measured using a 2-interval, forced-choice (2IFC) procedure. In each trial, a standard and a test stimulus were presented in random order. Each stimulus was preceded by a fixation cross, presented at the center of the screen for 1,000 ms, followed by a 2,000 ms-long period of blank screen in which the loudspeaker moved to its position. The actual stimulus lasted 100 ms, followed by either a 900 ms-long backward masker (visual stimuli) or a blank screen for 900 ms (auditory stimuli). After the second stimulus period was over, a response probe was displayed and participants indicated by button press which interval contained the stimulus that was farther to the right (Fig 1B). Visual feedback was provided for 500 ms immediately after the response was given. The inter-trial interval was 500 ms.

The standard stimulus was located straight-ahead at the center of the screen; the location of the test stimulus was controlled by four interleaved staircases, two of which had the test stimulus start at 12.5° (to the right of straight-ahead), and the other two at −12.5° (12.5° to the left of straight-ahead). For the two staircases starting at one side, one followed the two-down-one-up rule (with down being defined as moving the test stimulus leftwards) converging to a probability of 71% [75] of perceiving the test stimulus as farther to the right than the standard stimulus; the other staircase followed the one-down-two-up rule converging to a probability of 29%. The initial step size was 1.9°, decreased to 1.0° after the first staircase reversal and to 0.5° after the third reversal. Each staircase consisted of 40 trials, resulting in a total of 160 trials. To improve the estimation of the lapse rate, a test stimulus was presented distant from the center (±12.5°) once every 10 trials. A total of 176 trials was evenly split into 4 blocks.

Participants completed the spatial-discrimination task for each of the three levels of visual stimulus reliability in random order, the auditory stimulus was always tested last. Participants took about an hour to complete one stimulus condition; typically, they spread all four stimulus conditions across two days.

#### Bimodal spatial-discrimination task

Participants’ biases in auditory relative to visual spatial perception were measured using a spatial 2IFC procedure. An auditory and a high-reliability visual stimulus were presented in random order and participants indicated by button press whether the visual stimulus was located to the left or right of the auditory stimulus. The procedure was otherwise identical to that of the unimodal spatial-discrimination task (Fig 3A). No feedback was provided.

The auditory stimulus was presented at one of four locations (±2.5 or ±7.5°). We used the adaptive staircase procedure to effectively sample the visual stimulus space for each participant as the range of meaningful stimulus locations varied considerably across participants due to individual perceptual biases. Specifically, the location of the visual stimulus was controlled by eight interleaved staircases, two for each of the four auditory locations. Of the two staircases per auditory location, one started the test stimulus at 15° relative to the auditory location, and the other one at −15°. Staircases with the visual stimulus starting from the right of the auditory stimulus followed the one-down-two-up rule, converging to a probability of 29% of choosing the visual as to the right of the auditory stimulus. Staircases with the visual stimulus starting from the left of the auditory stimulus followed the two-down-one-up rule, converging to a probability of 71%. Staircase step size was updated as described above. Each staircase consisted of 36 trials. Trials with the visual stimulus being located at ±15° relative to the auditory stimulus were inserted once every nine trials, resulting in a total of 320 trials. The session was divided into six blocks. Usually, participants took about two hours to complete all trials.

#### Pointing practice task

Participants’ localization precision independent of spatial perception was measured using a localization task with visual stimuli of maximal spatial reliability. In each trial, a white square (8 × 8 pixels ≈ 0.6° × 0.6°) was displayed on the screen for 100 ms. The stimulus was followed by a 900 ms-long backward masker and 500 ms of blank screen. Subsequently, the response cursor, a green square of the same size as the white square, appeared on the screen. Participants used the pointing device to move the cursor to the stimulus position, and confirmed their response with the footpedal. Response times were unrestricted. The cursor location was shown during adjustment, but error feedback was not provided. There were eight possible horizontal positions for the stimulus, evenly spaced from -17.5 to 17.5° in steps of 5°. Each stimulus location was visited 30 times in random order, resulting in a total of 240 trials. The inter-trial interval was 500 ms. This experiment took 30 minutes to complete and was typically administered after the bimodal spatial-discrimination task on the same day.

#### Unimodal localization task (pre- and post-recalibration phase)

Participants’ baseline and post-recalibration spatial perception were measured using a unimodal spatial-localization task. In each trial, either an auditory or a visual stimulus was presented, again preceded by a fixation cross and a blank screen. The inter-trial interval was 100 ms, otherwise timing was identical to that of the discrimination tasks. Auditory stimulus locations were the same as in the bimodal spatial-discrimination task; visual stimulus locations were the four locations that were identified as perceptually co-located with those four auditory locations using the bimodal spatial-discrimination task. Participants again responded by moving a visual cursor to the stimulus location (Fig 5A). There was no time limit for the response. The location of the visual cursor was shown during the adjustment, but feedback about the localization error was not provided. Each of the four target locations per modality was tested 12 times, resulting in a total of 96 trials administered in pseudorandom order. These trials were split into four blocks. Usually participants took about 25 min to complete all 96 trials; they did so once at the beginning of the session (pre-recalibration phase) and once again after the recalibration phase (post-recalibration phase).

#### Bimodal localization task (recalibration phase)

During the audiovisual recalibration phase, participants were presented with temporally synchronous but spatially discrepant audiovisual stimuli. We asked them to localize either the auditory or the visual component, with the localization modality cued after stimulus presentation (Fig 5B), a procedure that has been associated with larger recalibration effects than non-spatial tasks [32]. All other trial parameters were identical to the unimodal localization task. Three audiovisual stimulus pairs were chosen such that an auditory stimulus location was paired with the visual location perceived as aligned with the auditory stimulus location to its left in the visual-left-of-auditory condition and the auditory stimulus location to its right in the visual-right-of-auditory condition (Fig 5C). Each of the three audiovisual pairs was repeated 40 times in random order, resulting in a total of 120 trials. These trials were split into four blocks. Usually, participants took about 30 min to complete all 120 trials. A full session (pre-recalibration, recalibration, and post-recalibration phase) took 80 min. Participants completed the six sessions on separate days.

### Data preparation and statistical analysis

#### Unimodal spatial-discrimination task

For the unimodal spatial-discrimination task, the data were coded as the probability of identifying the test stimulus as located farther to the right than the standard stimulus as a function of test stimulus location separately for each stimulus type (Fig 2A). These data were fitted with a cumulative Gaussian distribution centered at 0 and with a lapse rate constrained to be less than or equal to 6% [76]. The JND was calculated as half the distance between the stimulus locations corresponding to probabilities of 0.25 and 0.75 according to the fitted cumulative Gaussian distribution (unscaled by the lapse rate). To measure goodness of fit, we computed adjusted *R*^2^ values based on the binned data (bin size = 1.8°). To derive error bars, we randomly resampled the raw data with replacement 1,000 times, fitted psychometric functions to each resampled dataset, calculated the JND, and took the 2.5 and 97.5 percentiles of the 1,000 JNDs as the bootstrapped confidence interval.

#### Bimodal spatial-discrimination task

Data from the bimodal spatial-discrimination task were coded as the probability of identifying the visual stimulus as located to the right of the auditory stimulus as a function of visual stimulus location. We fitted four cumulative Gaussian distributions to these data, one for each auditory standard stimulus. Again, we included a lapse rate, constrained to be less than or equal to 6% [76]. Adjusted *R*^2^ values were calculated based on binned data (bin size = 3°). The point of subjective equality (PSE) was defined as the visual stimulus location corresponding to a probability of 0.5 according to the unscaled psychometric function. The four PSEs were modeled as a linear function of auditory stimulus location. From this linear regression of the PSEs, we computed the locations of the visual stimulus perceived as co-located with the four auditory locations. In the subsequent unimodal and bimodal spatial-localization tasks, we presented visual stimuli at these locations rather than those directly indicated by the PSEs to reduce effects of random noise during the bimodal spatial-discrimination task. 95% confidence intervals for each parameter were obtained as before.

#### Pointing practice task

Data from the pointing practice task, used to measure localization response precision, were not statistically analyzed but were filtered before the model fitting. For each stimulus location, we *z*-transformed the data by subtracting the mean localization response per stimulus location and then dividing by the standard deviation of all demeaned responses. Localization responses with a *z*-score outside of [−3, 3] were identified as outliers (0–1.67% of trials) and excluded from the model fitting.

#### Unimodal localization task (pre- and post-recalibration phase)

Data from the unimodal localization task were filtered separately for each modality, stimulus location and phase. To compute the means for the z-transformation, auditory and visual localization responses in the pre-recalibration phase were pooled across all six sessions, as the day of testing should not influence localization performance. In contrast, the means of auditory and visual localization responses in the post-recalibration phase were calculated separately for each of the six conditions as each recalibration condition should influence localization differently. Then, we computed the standard deviation of the demeaned auditory localization responses pooled across all six session and both phases. The standard deviation of demeaned visual localization responses was calculated separately for each visual-reliability condition given that stimulus reliability should influence localization precision. Localization responses identified as outliers (*z*-scores outside of [−3, 3]; 0.78–1.82% of trials) were excluded from all further analyses.

For statistical analysis and display, we summarized localization responses as a linear function of stimulus location, separately for each modality and phase. For each modality, localization responses in the pre-recalibration phase were pooled across all six sessions (visual reliability should influence the precision but not the accuracy of the localization responses; Section S7 in S1 Appendix). Responses from the post-recalibration phase were regressed separately for each condition, because each condition should influence localization differently. The seven regression lines per modality were fit with the constraint that all have the same slope. The amount of recalibration of one modality by the other was defined as the distance between the intercepts of the regression lines for pre- and post-recalibration localization responses. It was coded as positive if localization responses in the post-recalibration phase were shifted to compensate for the audiovisual discrepancy in the preceding recalibration phase. For the statistical analysis, we calculated the amount of recalibration for each modality and recalibration condition (2 recalibration directions × 3 reliability levels). To derive confidence intervals, localization responses were resampled, separately for each location, task, session, and modality.

Data from the bimodal spatial-localization task conducted during the audiovisual recalibration phase were not analyzed. Data analysis was done using Python 3.7, R 4.0.2, and MATLAB 2019a.

## Models of audiovisual recalibration

In this section we lay out the definition of recalibration underlying all models reported here and then describe the recalibration process during the audiovisual recalibration phase according to each of the models. Finally, we provide a formalization of each of the tasks used to constrain the model parameters followed by the details of how the models were fit to the data.

### Definition of recalibration

Each stimulus at location *s* in the world leads to a sensory measurement *m*′ in an observer’s brain. This measurement is corrupted by Gaussian-distributed sensory noise. Thus, with repeated presentations of stimuli at location *s*, the sensory measurements correspond to scattered spatial locations $m^{\prime }∼N\left(s^{\prime },{\sigma^{\prime }}^{2}\right)$. The variability of the measurements is determined by the stimulus reliability 1/*σ*′^2^. To allow integration of information from different modalities, measurements are remapped into a common internal reference frame. Hence, the measurement distribution is centered on *s*′, the remapped location of *s*. As part of the remapping process, spatial discrepancies between the senses are accounted for by shifting the measurements by a modality-specific amount Δ. We model recalibration as the process of updating these shifts following each encounter with a cross-modal stimulus pair [32, 63] and probed this updating process by misaligning the physical visual and auditory stimuli to create an artificial sensory discrepancy.

We assumed that observers were calibrated as far as possible at the beginning of each experimental session. Thus, the remapped stimulus locations in internal space were understood as linear functions of the physical stimulus locations *s*_*A*_ and *s*_*V*_, that is, $s_{A}^{\prime }=a_{A}s_{A}+b_{A}$ and $s_{V}^{\prime }=a_{V}s_{V}+b_{V}$. However, given that we can only measure relative biases there is no way to empirically isolate the remapping of one modality. As a consequence and without loss of generality, we set $s_{V}^{\prime }$ to be equal to *s*_*V*_ (i.e., *a*_*V*_ = 1 and *b*_*V*_ = 0).

We use the variables $\Delta_{A_{i}}$ and $\Delta_{V_{i}}$ to exclusively capture the update in measurement shifts after encounters with the spatially discrepant audiovisual stimulus pairs with visual reliability *i* (*i* ∈ {1, 2, 3}) during the audiovisual recalibration phase. In addition, we assumed that $\Delta_{A_{i}}$ and $\Delta_{V_{i}}$ are updated in every trial of the phase, and thus the location-independent shifts at the end of the task can be written as the sum of the initial shifts and the shift updates over 120 trials, that is $s_{A}^{\prime }=a_{A}s_{A}+b_{A}+\Delta_{A_{i}}\left(121\right)$ and $s_{V}^{\prime }=s_{V}+\Delta_{V_{i}}\left(121\right)$. The final shifts accumulated after 120 recalibration trials were assumed be maintained throughout the subsequent post-recalibration task as observers were not exposed to spatially aligned audiovisual pairs after the recalibration phase (Section S12 in S1 Appendix).

We further assumed that stimulus reliability differed between unimodal ($1/{\sigma_{A}^{\prime }}^{2}$) and bimodal ($1/{\sigma_{AV,A}^{\prime }}^{2}$) stimulus presentations. Note that we denote the visual-reliability condition for variables associated with the auditory modality (i.e., with a subscript *A*_*i*_) when the value of that variable can be impacted by visual measurements (e.g., shifts $\Delta_{A_{i}}$ or, below, sensory estimates ${\hat{s}}_{A_{i}}^{\prime }$), but not otherwise (e.g., measurements $m_{A}^{\prime }$ or measurement variances ${\sigma_{A}^{\prime }}^{2}$ and ${\sigma_{AV,A}^{\prime }}^{2}$).

### Models of the recalibration process

#### The reliability-based model of cross-modal recalibration

According to this model, each modality should be recalibrated in the direction of the other modality by an amount that is proportional to the other modality’s relative reliability [53]. In other words, after every trial, the measurement shifts, $\Delta_{A_{i}}$ and $\Delta_{V_{i}}$, are updated in the direction of the discrepancy between the visual and auditory measurements by an amount proportional to the two modalities’ relative reliabilities as follows:

$$\begin{array}{c} \Delta_{A_{i}}\left(t+1\right)=\Delta_{A_{i}}\left(t\right)+\alpha w_{i}(m_{V_{i},l_{V}\left(t\right)}^{\prime }-m_{A,l_{A}\left(t\right)}^{\prime }), \end{array}$$ (1)

where

$$\begin{array}{c} w_{i}=\frac{{\sigma_{AV,V_{i}}^{\prime }}^{-2}}{{\sigma_{AV,V_{i}}^{\prime }}^{-2}+{\sigma_{AV,A}^{\prime }}^{-2}} \end{array}$$ (2)

and analogously

$$\begin{array}{c} \Delta_{V_{i}}\left(t+1\right)=\Delta_{V_{i}}\left(t\right)+\alpha \left(1-w_{i}\right)(m_{A,l_{A}\left(t\right)}^{\prime }-m_{V_{i},l_{V}\left(t\right)}^{\prime }), \end{array}$$ (3)

where *l*_*A*_ and *l*_*V*_ index the auditory and visual locations (*l*_*A*_, *l*_*V*_ ∈ {1, 2, 3, 4}), *α* denotes a supra-modal learning rate, and *t* denotes trial number.

#### The fixed-ratio model of cross-modal recalibration

According to this model, after every trial, $\Delta_{A_{i}}$ and $\Delta_{V_{i}}$ are updated in the direction of the discrepancy between the visual and auditory measurements by a fixed ratio of this discrepancy. The ratio of the update depends solely on the identity of the modality and thus is independent of stimulus reliability [62]. $\Delta_{A_{i}}$ and $\Delta_{V_{i}}$ are updated according to the following equations:

$$\begin{array}{c} \Delta_{A_{i}}\left(t+1\right)=\Delta_{A_{i}}\left(t\right)+\alpha_{A}(m_{V_{i},l_{V}\left(t\right)}^{\prime }-m_{A,l_{A}\left(t\right)}^{\prime }) \end{array}$$ (4)

and

$$\begin{array}{c} \Delta_{V_{i}}\left(t+1\right)=\Delta_{V_{i}}\left(t\right)+\alpha_{V}(m_{A,l_{A}\left(t\right)}^{\prime }-m_{V_{i},l_{V}\left(t\right)}^{\prime }), \end{array}$$ (5)

where *α*_*A*_ and *α*_*V*_ are modality-specific learning rates.

#### The causal-inference model of recalibration

In this model, the shift updates are determined by the discrepancy between a measurement and the corresponding perceptual estimate, ${\hat{s}}_{A_{i},l_{A}\left(t\right)}^{\prime }-m_{A,l_{A}\left(t\right)}^{\prime }$ and ${\hat{s}}_{V_{i},l_{V}\left(t\right)}^{\prime }-m_{V_{i},l_{V}\left(t\right)}^{\prime }$ [63] for each modality. The spatial discrepancy between auditory and visual measurements and the relative reliabilities of both stimuli have indirect influence on the shift updates by means of their influence on the location estimates, ${\hat{s}}_{A_{i},l_{A}\left(t\right)}^{\prime }$ and ${\hat{s}}_{V_{i},l_{V}\left(t\right)}^{\prime }$. Additionally, the location estimates and thus the shift updates are contingent on the degree to which the brain infers a common cause or separate causes for the two measurements [30, 63].

The location estimates are a mixture of two conditional location estimates, one for each causal scenario (common audiovisual source, *C* = 1, or different auditory and visual sources, *C* = 2). In the case of a common source, the location estimate of the audiovisual stimulus pair, ${\hat{s}}_{A_{i},l_{A}\left(t\right),C=1}^{\prime }={\hat{s}}_{V_{i},l_{V}\left(t\right),C=1}^{\prime }$, equals the reliability-weighted average of the measurements $m_{A,l_{A}\left(t\right)}^{\prime }$, $m_{V_{i},l_{V}\left(t\right)}^{\prime }$ and the mean $\mu_{P}^{\prime }$ of an internal, Gaussian-shaped, supra-modal prior across stimulus locations with variance ${\sigma_{P}^{\prime }}^{2}$:

$$\begin{array}{c} {\hat{s}}_{A_{i},l_{A}\left(t\right),C=1}^{\prime }={\hat{s}}_{V_{i},l_{V}\left(t\right),C=1}^{\prime }=\frac{m_{A,l_{A}\left(t\right)}^{\prime }{\sigma_{AV,A}^{\prime }}^{-2}+m_{V_{i},l_{V}\left(t\right)}^{\prime }{\sigma_{AV,V_{i}}^{\prime }}^{-2}+\mu_{P}^{\prime }{\sigma_{P}^{\prime }}^{-2}}{{\sigma_{AV,A}^{\prime }}^{-2}+{\sigma_{AV,V_{i}}^{\prime }}^{-2}+{\sigma_{P}^{\prime }}^{-2}}. \end{array}$$ (6)

In the case of two separate sources, the location estimates of the auditory and the visual stimulus, ${\hat{s}}_{A_{i},l_{A}\left(t\right),C=2}^{\prime }$ and ${\hat{s}}_{V_{i},l_{V}\left(t\right),C=2}^{\prime }$, are equal to the reliability-weighted averages of $m_{A,l_{A}\left(t\right)}^{\prime }$ and $\mu_{P}^{\prime }$ for the auditory estimate, and $m_{V_{i},l_{V}\left(t\right)}^{\prime }$ and $\mu_{P}^{\prime }$ for the visual estimate, respectively:

$$\begin{array}{c} {\hat{s}}_{A_{i},l_{A}\left(t\right),C=2}^{\prime }=\frac{m_{A,l_{A}\left(t\right)}^{\prime }{\sigma_{AV,A}^{\prime }}^{-2}+\mu_{P}^{\prime }{\sigma_{P}^{\prime }}^{-2}}{{\sigma_{AV,A}^{\prime }}^{-2}+{\sigma_{P}^{\prime }}^{-2}} \end{array}$$ (7)

and

$$\begin{array}{c} {\hat{s}}_{V_{i},l_{V}\left(t\right),C=2}^{\prime }=\frac{m_{V_{i},l_{V}\left(t\right)}^{\prime }{\sigma_{AV,V_{i}}^{\prime }}^{-2}+\mu_{P}^{\prime }{\sigma_{P}^{\prime }}^{-2}}{{\sigma_{AV,V_{i}}^{\prime }}^{-2}+{\sigma_{P}^{\prime }}^{-2}}. \end{array}$$ (8)

The final location estimates are derived by model averaging (see alternative decision strategy in Section S3 in S1 Appendix). Specifically, the final location estimate ${\hat{s}}_{A_{i},l_{A}\left(t\right)}^{\prime }$ is the average of the conditional location estimates, ${\hat{s}}_{A_{i},l_{A}\left(t\right),C=1}^{\prime }$ and ${\hat{s}}_{A_{i},l_{A}\left(t\right),C=2}^{\prime }$, with each estimate weighted by the posterior probability of its causal structure:

$$\begin{array}{c} \begin{array}{cc} {\hat{s}}_{A_{i},l_{A}\left(t\right)}^{\prime }= & {\hat{s}}_{A_{i},l_{A}\left(t\right),C=1}^{\prime }P\left(C=1|m_{A,l_{A}\left(t\right)}^{\prime },m_{V_{i},l_{V}\left(t\right)}^{\prime }\right)\\ & +{\hat{s}}_{A_{i},l_{A}\left(t\right),C=2}^{\prime }(1-P(C=1|m_{A,l_{A}\left(t\right)}^{\prime },m_{V_{i},l_{V}\left(t\right)}^{\prime })) \end{array} \end{array}$$ (9)

and analogously for the visual location estimate:

$$\begin{array}{c} \begin{array}{cc} {\hat{s}}_{V_{i},l_{V}\left(t\right)}^{\prime }= & {\hat{s}}_{V_{i},l_{V}\left(t\right),C=1}^{\prime }P\left(C=1|m_{A,l_{A}\left(t\right)}^{\prime },m_{V_{i},l_{V}\left(t\right)}^{\prime }\right)\\ & +{\hat{s}}_{V_{i},l_{V}\left(t\right),C=2}^{\prime }(1-P(C=1|m_{A,l_{A}\left(t\right)}^{\prime },m_{V_{i},l_{V}\left(t\right)}^{\prime })). \end{array} \end{array}$$ (10)

The posterior probability of a common source for the auditory and visual measurements in trial *t*, $P(C=1|m_{A,l_{A}\left(t\right)}^{\prime },m_{V_{i},l_{V}\left(t\right)}^{\prime })$, is proportional to the product of the likelihood of a common source for these measurements in trial *t* and the prior probability of a common source for visual and auditory measurements in general, *P*(*C* = 1):

$$\begin{array}{c} \begin{array}{cc} & P(C=1|m_{A,l_{A}\left(t\right)}^{\prime },m_{V_{i},l_{V}\left(t\right)}^{\prime })=\\ & \frac{P\left(m_{A,l_{A}\left(t\right)}^{\prime },m_{V_{i},l_{V}\left(t\right)}^{\prime }|C=1\right)P\left(C=1\right)}{P\left(m_{A,l_{A}\left(t\right)}^{\prime },m_{V_{i},l_{V}\left(t\right)}^{\prime }|C=1\right)P\left(C=1\right)+P\left(m_{A,l_{A}\left(t\right)}^{\prime },m_{V_{i},l_{V}\left(t\right)}^{\prime }|C=2\right)\left(1-P\left(C=1\right)\right)}. \end{array} \end{array}$$ (11)

The posterior probability of two separate sources, one for the auditory and one for the visual measurement, is $1-P(C=1|m_{A,l_{A}\left(t\right)}^{\prime },m_{V_{i},l_{V}\left(t\right)}^{\prime })$.

The likelihood of a common source of the visual and auditory measurements in trial *t*, $P(m_{A,l_{A}\left(t\right)}^{\prime },m_{V_{i},l_{V}\left(t\right)}^{\prime }|C=1)$, is the product of the likelihood of the internally represented audiovisual stimulus location $s_{AV}^{\prime }$ given the auditory measurement, $m_{A,l_{A}\left(t\right)}^{\prime }$, and the visual measurement $m_{V_{i},l_{V}\left(t\right)}^{\prime }$, and the supra-modal prior, integrated over all possible remapped audiovisual stimulus locations $s_{AV}^{\prime }$ [30]:

$$\begin{array}{c} \begin{array}{cc} & P\left(m_{A,l_{A}\left(t\right)}^{\prime },m_{V_{i},l_{V}\left(t\right)}^{\prime }|C=1\right)=\int P\left(m_{A,l_{A}\left(t\right)}^{\prime }|s_{AV}^{\prime }\right)P\left(m_{V_{i},l_{V}\left(t\right)}^{\prime }|s_{AV}^{\prime }\right)P\left(s_{AV}^{\prime }\right)ds_{AV}^{\prime }\\ & =\frac{1}{2\pi \sqrt{{\sigma_{AV,A}^{\prime }}^{2}{\sigma_{AV,V_{i}}^{\prime }}^{2}+{\sigma_{AV,A}^{\prime }}^{2}{\sigma_{P}^{\prime }}^{2}+{\sigma_{AV,V_{i}}^{\prime }}^{2}{\sigma_{P}^{\prime }}^{2}}}\times \\ & \text{exp}[\;-\;\frac{1}{2}\frac{{\left(m_{A,l_{A}\left(t\right)}^{\prime }\;-\;m_{V_{i},l_{V}\left(t\right)}^{\prime }\right)}^{2}{\sigma_{P}^{\prime }}^{2}\;+\;{\left(m_{A,l_{A}\left(t\right)}^{\prime }\;-\;\mu_{P}^{\prime }\right)}^{2}{\sigma_{AV,V_{i}}^{\prime }}^{2}\;+\;{\left(m_{V_{i},l_{V}\left(t\right)}^{\prime }\;-\;\mu_{P}^{\prime }\right)}^{2}{\sigma_{AV,A}^{\prime }}^{2}}{{\sigma_{AV,A}^{\prime }}^{2}{\sigma_{AV,V_{i}}^{\prime }}^{2}+{\sigma_{AV,A}^{\prime }}^{2}{\sigma_{P}^{\prime }}^{2}+{\sigma_{AV,V_{i}}^{\prime }}^{2}{\sigma_{P}^{\prime }}^{2}}]. \end{array} \end{array}$$ (12)

The likelihood of different sources for the visual and auditory measurements in trial *t*, $P(m_{A,l_{A}\left(t\right)}^{\prime },m_{V_{i},l_{V}\left(t\right)}^{\prime }|C=2)$ is the product of the likelihood of internally represented auditory and visual stimulus locations $s_{A}^{\prime }$ and $s_{V}^{\prime }$ given the auditory measurement, $m_{A,l_{A}\left(t\right)}^{\prime }$, and the visual measurement $m_{V_{i},l_{V}\left(t\right)}^{\prime }$, and the supra-modal prior. Given that the measurements in this causal scenario stem from different sources, the product is integrated over all possible, remapped visual and auditory stimulus locations, $s_{V}^{\prime }$ and $s_{A}^{\prime }$:

$$\begin{array}{c} \begin{array}{cc} P(m_{A,l_{A}\left(t\right)}^{\prime } & ,m_{V_{i},l_{V}\left(t\right)}^{\prime }\left|C=2\right)=\;\int \int \;P\left(m_{A,l_{A}\left(t\right)}^{\prime },m_{V_{i},l_{V}\left(t\right)}^{\prime }|s_{A}^{\prime },s_{V}^{\prime }\right)P\left(s_{A}^{\prime },s_{V}^{\prime }\right)ds_{A}^{\prime }ds_{V}^{\prime }\\ & =(\int P\left(m_{A,l_{A}\left(t\right)}^{\prime }|s_{A}^{\prime }\right)P\left(s_{A}^{\prime }\right)ds_{A}^{\prime })(\int P\left(m_{V_{i},l_{V}\left(t\right)}^{\prime }|s_{V}^{\prime }\right)P\left(s_{V}^{\prime }\right)ds_{V}^{\prime })\\ & =\frac{1}{2\pi \sqrt{\left({\sigma_{AV,A}^{\prime }}^{2}+{\sigma_{P}^{\prime }}^{2}\right)\left({\sigma_{AV,V_{i}}^{\prime }}^{2}+{\sigma_{P}^{\prime }}^{2}\right)}}\times \\ & \text{exp}[-\frac{1}{2}(\frac{{\left(m_{A,l_{A}\left(t\right)}^{\prime }-\mu_{P}^{\prime }\right)}^{2}}{{\sigma_{AV,A}^{\prime }}^{2}+{\sigma_{P}^{\prime }}^{2}}+\frac{{\left(m_{V_{i},l_{V}\left(t\right)}^{\prime }-\mu_{P}^{\prime }\right)}^{2}}{{\sigma_{AV,V_{i}}^{\prime }}^{2}+{\sigma_{P}^{\prime }}^{2}})]. \end{array} \end{array}$$ (13)

The updates of the shifts are scaled by two modality-specific learning rates, *α*_*A*_ and *α*_*V*_ [63]:

$$\begin{array}{c} \Delta_{A_{i}}\left(t+1\right)=\Delta_{A_{i}}\left(t\right)+\alpha_{A}({\hat{s}}_{A_{i},l_{A}\left(t\right)}^{\prime }-m_{A,l_{A}\left(t\right)}^{\prime }) \end{array}$$ (14)

and

$$\begin{array}{c} \Delta_{V_{i}}\left(t+1\right)=\Delta_{V_{i}}\left(t\right)+\alpha_{V}({\hat{s}}_{V_{i},l_{V}\left(t\right)}^{\prime }-m_{V_{i},l_{V}\left(t\right)}^{\prime }). \end{array}$$ (15)

We also tested a version of the model with one supra-modal learning rate (*α* = *α*_*A*_ = *α*_*V*_).

### Formalization of the tasks

#### Unimodal spatial-discrimination task

The unimodal spatial-discrimination task was conducted to estimate the one auditory ${1/\sigma_{A}^{\prime }}^{2}$ and three visual $1/{\sigma_{V_{i}}^{\prime }}^{2}$ stimulus reliabilities under unimodal presentation conditions as well as to constrain the estimates of the variable bias *a*_*A*_ introduced by the remapping process. We begin by describing the auditory version. The standard stimulus was presented straight ahead, at location *s*_*A*,0_, and the test stimulus was presented at one of *N*_*A*_ locations, *s*_*A*,*n*_, determined by an adaptive procedure. For each pair, the probability, *p*_*A*,*n*_, of estimating the test stimulus to be located to the right of the standard stimulus is a function of the physical distance between the two stimuli.

We assume that the observer makes the decision by comparing the internal location estimates ${\hat{s}}_{A,n}^{\prime }$ and ${\hat{s}}_{A,0}^{\prime }$,

$$\begin{array}{c} p_{A,n}=P\left({\hat{s}}_{A,n}^{\prime }>{\hat{s}}_{A,0}^{\prime }\right)=P\left({\hat{s}}_{A,n}^{\prime }-{\hat{s}}_{A,0}^{\prime }>0\right). \end{array}$$ (16)

To further specify *p*_*A*,*n*_, we have to derive the probability distributions of the internal location estimates. Each physical stimulus at location *s*_*A*,*n*_ results in an internal measurement, $m_{A,n}^{\prime }$. The measurement distribution is Gaussian ($m_{A,n}^{\prime }∼N\left(s_{A,n}^{\prime },{\sigma_{A}^{\prime }}^{2}\right)$) and for a given measurement the estimate of the remapped location of the stimulus is the average of the measurement and the mean of the spatial prior, $\mu_{P}^{\prime }$, each weighted by their relative reliabilities (${\hat{s}}_{A,n}^{\prime }=\frac{{\sigma_{A}^{\prime }}^{-2}}{{\sigma_{A}^{\prime }}^{-2}+{\sigma_{P}^{\prime }}^{-2}}m_{A,n}^{\prime }+\frac{{\sigma_{P}^{\prime }}^{-2}}{{\sigma_{A}^{\prime }}^{-2}+{\sigma_{P}^{\prime }}^{-2}}\mu_{P}^{\prime }$). Thus, the probability distribution of the location estimates of a test stimulus is

$$\begin{array}{c} {\hat{s}}_{A,n}^{\prime }∼N(\mu_{{\hat{s}}_{A,n}^{\prime }},\sigma_{{\hat{s}}_{A}^{\prime }}^{2}) \end{array}$$ (17)

where

$$\begin{array}{c} \mu_{{\hat{s}}_{A,n}^{\prime }}=\frac{{\sigma_{A}^{\prime }}^{-2}}{{\sigma_{A}^{\prime }}^{-2}+{\sigma_{P}^{\prime }}^{-2}}s_{A,n}^{\prime }+\frac{{\sigma_{P}^{\prime }}^{-2}}{{\sigma_{A}^{\prime }}^{-2}+{\sigma_{P}^{\prime }}^{-2}}\mu_{P}^{\prime }\;\text{and}\;\sigma_{{\hat{s}}_{A}^{\prime }}^{2}={\left(\frac{{\sigma_{A}^{\prime }}^{-2}}{{\sigma_{A}^{\prime }}^{-2}+{\sigma_{P}^{\prime }}^{-2}}\right)}^{2}{\sigma_{A}^{\prime }}^{2} \end{array}$$ (18)

The probability distribution of the difference between the two location estimates ${\hat{s}}_{A,n}^{\prime }$ and ${\hat{s}}_{A,0}^{\prime }$ is

$$\begin{array}{c} {\hat{s}}_{A,n}^{\prime }-{\hat{s}}_{A,0}^{\prime }∼N\left(\mu_{{\hat{s}}_{A,n}^{\prime }}-\mu_{{\hat{s}}_{A,0}^{\prime }},2\sigma_{{\hat{s}}_{A}^{\prime }}^{2}\right), \end{array}$$ (19)

where $\mu_{{\hat{s}}_{A,0}^{\prime }}=\frac{{\sigma_{A}^{\prime }}^{-2}}{{\sigma_{A}^{\prime }}^{-2}+{\sigma_{P}^{\prime }}^{-2}}s_{A,0}^{\prime }+\frac{{\sigma_{P}^{\prime }}^{-2}}{{\sigma_{A}^{\prime }}^{-2}+{\sigma_{P}^{\prime }}^{-2}}\mu_{P}^{\prime }$. Taken together, the probability of perceiving an auditory test stimulus at location *s*_*A*,*n*_ to the right of an auditory standard stimulus at location *s*_*A*,0_ is

$$\begin{array}{c} \begin{array}{cc} p_{A,n} & =P({\hat{s}}_{A,n}^{\prime }-{\hat{s}}_{A,0}^{\prime }>0)\\ & =1-\Phi (0;\mu_{{\hat{s}}_{A,n}^{\prime }}-\mu_{{\hat{s}}_{A,0}^{\prime }},2\sigma_{{\hat{s}}_{A}^{\prime }}^{2})\\ & =1-\Phi (0;\frac{{\sigma_{A}^{\prime }}^{-2}}{{\sigma_{A}^{\prime }}^{-2}+{\sigma_{P}^{\prime }}^{-2}}\left(s_{A,n}^{\prime }-s_{A,0}^{\prime }\right),2{\left(\frac{{\sigma_{A}^{\prime }}^{-2}}{{\sigma_{A}^{\prime }}^{-2}+{\sigma_{P}^{\prime }}^{-2}}\right)}^{2}{\sigma_{A}^{\prime }}^{2})\\ & =\Phi (s_{A,n}^{\prime }-s_{A,0}^{\prime };0,2{\sigma_{A}^{\prime }}^{2}), \end{array} \end{array}$$ (20)

where Φ(*x*;*μ*, *σ*^2^) is the cumulative Gaussian distribution.

However, as experimenters we only have access to response probabilities as a function of the stimulus locations in physical space. Given that the remapped location of $s_{A}^{\prime }$ is a function of the physical stimulus location *s*_*A*_, we can rewrite Eq 20 as

$$\begin{array}{c} \begin{array}{cc} p_{A,n} & =\Phi (\left(a_{A}s_{A,n}+b_{A}\right)-\left(a_{A}s_{A,0}+b_{A}\right);0,2{\sigma_{A}^{\prime }}^{2})\\ & =\Phi (s_{A,n}-s_{A,0};0,2a_{A}^{-2}{\sigma_{A}^{\prime }}^{2}), \end{array} \end{array}$$ (21)

and analogously,

$$\begin{array}{c} p_{V_{i},n}=\Phi \left(s_{V_{i},n}-s_{V_{i},0};0,2{\sigma_{V_{i}}^{\prime }}^{2}\right). \end{array}$$ (22)

Finally, the model includes occasional response lapses (i.e., random button presses) at rate λ, so that the probability of reporting the test stimulus as located farther to the right than the standard (*r*_*A*,*n*_ = 1) is

$$\begin{array}{c} p_{r_{A,n}=1}=0.5\lambda_{A}+\left(1-\lambda_{A}\right)p_{A,n}\;\text{and}\;p_{r_{V_{i},n}=1}=0.5\lambda_{V_{i}}+\left(1-\lambda_{V_{i}}\right)p_{V_{i},n}. \end{array}$$ (23)

#### Bimodal spatial-discrimination task

The bimodal spatial-discrimination task was conducted to estimate the relative bias of auditory compared to visual spatial perception, i.e., to estimate *a*_*A*_ and *b*_*A*_. Auditory stimuli were presented at four different locations in physical space *s*_*A*,*l*_, where *l* indexes the auditory location. Guided by a staircase procedure, on each trial *t*, an auditory stimulus at location *s*_*A*,*l*_ was paired with a visual stimulus of high spatial reliability (*i* = 1) at one of *N* test locations *s*_*V*,*n*_, where *n* indexes the finer grid of locations of visual stimuli that were presented during the task. For each pair, the model predicts *p*_*l*,*n*_, the probability of judging the visual stimulus at location *s*_*V*,*n*_ as to the right of the auditory stimulus at location *s*_*A*,*l*_.

Analogous to the unimodal spatial-discrimination task, we specify the probability distributions of the internal auditory and visual location estimates as

$$\begin{array}{c} {\hat{s}}_{A,l}^{\prime }∼N\left(\mu_{{\hat{s}}_{A,l}^{\prime }},\sigma_{{\hat{s}}_{A}^{\prime }}^{2}\right)\;\text{and}\;{\hat{s}}_{V_{1},n}^{\prime }∼N\left(\mu_{{\hat{s}}_{V_{1},n}},\sigma_{{\hat{s}}_{V_{1}}}^{2}\right), \end{array}$$ (24)

where

$$\begin{array}{c} \mu_{{\hat{s}}_{A,l}^{\prime }}=\frac{{\sigma_{A}^{\prime }}^{-2}s_{A,l}^{\prime }+{\sigma_{P}^{\prime }}^{-2}\mu_{P}^{\prime }}{{\sigma_{A}^{\prime }}^{-2}+{\sigma_{P}^{\prime }}^{-2}}=\frac{{\sigma_{A}^{\prime }}^{-2}\left(a_{A}s_{A,l}+b_{A}\right)+{\sigma_{P}^{\prime }}^{-2}\mu_{P}^{\prime }}{{\sigma_{A}^{\prime }}^{-2}+\sigma_{P}^{\prime -2}}, \end{array}$$ (25)

$$\begin{array}{c} \mu_{{\hat{s}}_{V_{1},n}^{\prime }}=\frac{{\sigma_{V_{1}}^{\prime }}^{-2}s_{V,n}^{\prime }+{\sigma_{P}^{\prime }}^{-2}\mu_{P}^{\prime }}{{\sigma_{V_{1}}^{\prime }}^{-2}+{\sigma_{P}^{\prime }}^{-2}}=\frac{{\sigma_{V_{1}}^{\prime }}^{-2}s_{V,n}+{\sigma_{P}^{\prime }}^{-2}\mu_{P}^{\prime }}{{\sigma_{V_{1}}^{\prime }}^{-2}+{\sigma_{P}^{\prime }}^{-2}}, \end{array}$$ (26)

$$\begin{array}{c} \sigma_{{\hat{s}}_{A}^{\prime }}^{2}=\frac{{\sigma_{A}^{\prime }}^{-2}}{{\left({\sigma_{A}^{\prime }}^{-2}+{\sigma_{P}^{\prime }}^{-2}\right)}^{2}}\;\text{and}\;\sigma_{{\hat{s}}_{V_{1}}^{\prime }}^{2}=\frac{{\sigma_{V_{1}}^{\prime }}^{-2}}{{\left({\sigma_{V_{1}}^{\prime }}^{-2}+{\sigma_{P}^{\prime }}^{-2}\right)}^{2}}. \end{array}$$ (27)

The probability of the observer perceiving a visual stimulus at physical location *s*_*V*,*n*_ as located to the right of an auditory stimulus at physical location *s*_*A*,*l*_ is thus:

$$\begin{array}{c} p_{l,n}=P\left({\hat{s}}_{V_{1},n}^{\prime }>{\hat{s}}_{A,l}^{\prime }\right)=P\left({\hat{s}}_{V_{1},n}^{\prime }-{\hat{s}}_{A,l}^{\prime }>0\right). \end{array}$$ (28)

The distribution of this difference is:

$$\begin{array}{c} {\hat{s}}_{V_{1},n}^{\prime }-{\hat{s}}_{A,l}^{\prime }∼N\left(\mu_{{\hat{s}}_{V_{1},n}^{\prime }}-\mu_{{\hat{s}}_{A,l}^{\prime }},{\sigma_{{\hat{s}}_{V_{1}}^{\prime }}}^{2}+{\sigma_{{\hat{s}}_{A}^{\prime }}}^{2}\right). \end{array}$$ (29)

The probability of perceiving the visual stimulus to the right of the auditory can then be expressed as

$$\begin{array}{c} p_{l,n}=1-\Phi \left(0;\mu_{{\hat{s}}_{V_{1},n}^{\prime }}-\mu_{{\hat{s}}_{A,l}^{\prime }},\sigma_{{\hat{s}}_{V_{1}}^{\prime }}^{2}+\sigma_{{\hat{s}}_{A}^{\prime }}^{2}\right). \end{array}$$ (30)

As in the unimodal spatial-discrimination task, the model includes occasional lapses at rate λ_*AV*_. Therefore, the probability of reporting a visual stimulus at *s*_*V*,*n*_ as located to the right of an auditory stimulus at location *s*_*A*,*l*_ (*r*_*l*,*n*_ = 1) is equal to

$$\begin{array}{c} p_{r_{l,n}=1}=0.5\lambda_{AV}+\left(1-\lambda_{AV}\right)p_{l,n}. \end{array}$$ (31)

#### Pointing practice task

The pointing practice task was used to estimate localization response variability, $\sigma_{r}^{2}$, due to sources unrelated to the spatial perception of the stimuli. Visual stimuli were presented at eight different locations *s*_*V*,*o*_, where *o* indexes the stimulus location (*o* ∈ {1, 2, …, 8}). Localization responses (i.e., confirmed cursor positions) in each trial were modeled as perturbed by Gaussian-distributed noise and centered on the physical stimulus location:

$$\begin{array}{c} r_{V,o}∼N(s_{V,o},\sigma_{r}^{2}). \end{array}$$ (32)

By doing so, we assume that 1) the location of the visual cursor in physical space maps directly to its location in perceptual space and 2) the stimulus location estimate is unbiased. This is based on our general assumption of identity remapping for visual stimuli as well as on the high spatial reliability of the visual cursor and the visual stimulus, which should safeguard their estimates against the influence of spatial priors. See Section S2 in S1 Appendix for a model that does not have these assumptions.

#### Unimodal localization task—Pre-recalibration phase

The unimodal localization task was conducted before and after the recalibration phase to measure shifts in auditory and visual localization responses as a consequence of exposure to spatially discrepant audiovisual stimuli during the recalibration phase. Stimuli were presented at four locations for each modality, *s*_*A*,*l*_ and *s*_*V*,*l*_.

As for the pointing practice task, we assumed that the probability distributions of the localization responses in physical space, *r*_*A*,*l*_ and $r_{V_{i},l}$, are centered on the location estimates ${\hat{s}}_{A,l}^{\prime }$ and ${\hat{s}}_{V_{i},l}^{\prime }$ in perceptual space, and corrupted by additional unbiased noise. As in the spatial-discrimination tasks (and unlike in the pointing practice task that used a different, maximally reliable stimulus), the stimulus location estimates are assumed to be biased due to the remapping process and the incorporation of the supra-modal spatial prior. It follows that the probability distributions of the localization responses are

$$\begin{array}{c} r_{A,l}∼N(\mu_{{\hat{s}}_{A,l}^{\prime }},\sigma_{{\hat{s}}_{A}^{\prime }}^{2}+\sigma_{r}^{2})\;\text{and}\;r_{V_{i},l}∼N(\mu_{{\hat{s}}_{V_{i},l}^{\prime }},\sigma_{{\hat{s}}_{V_{i}}^{\prime }}^{2}+\sigma_{r}^{2}), \end{array}$$ (33)

where the terms are defined in Eqs 24–27.

#### Unimodal localization task—Post-recalibration phase

In the post-recalibration phase, the remapping from physical to perceptual space had been updated so that additional shifts $\Delta_{A_{i}}$ and $\Delta_{V_{i}}$, accumulated after 120 exposures to discrepant audiovisual stimuli, were incorporated: $s_{A}^{\prime }=a_{A}s_{A}+b_{A}+\Delta_{A_{i}}\left(121\right)$ and $s_{V}^{\prime }=a_{V}s_{V}+b_{V}+\Delta_{V_{i}}\left(121\right)$. This change in the measurement distributions affects the centers of the location estimates’ probability distributions as follows:

$$\begin{array}{c} \begin{array}{cc} \mu_{{\hat{s}}_{A_{i},l}^{\prime }} & =\frac{{\sigma_{A}^{\prime }}^{-2}\left(a_{A}s_{A,l}+b_{A}+\Delta_{A_{i}}\left(121\right)\right)+{\sigma^{\prime }}_{P}^{-2}\mu_{P}^{\prime }}{{\sigma_{A}^{\prime }}^{-2}+{\sigma^{\prime }}_{P}^{-2}}\\ \mu_{{\hat{s}}_{V_{i},l}^{\prime }} & =\frac{{\sigma_{V_{i}}^{\prime }}^{-2}\left(s_{V,l}+\Delta_{V_{i}}\left(121\right)\right)+{\sigma^{\prime }}_{P}^{-2}\mu_{P}^{\prime }}{{\sigma_{V_{i}}^{\prime }}^{-2}+{\sigma^{\prime }}_{P}^{-2}}. \end{array} \end{array}$$ (34)

We assumed that the probability distributions of the localization responses are centered on these updated values, that is, we did not implement the updated remapping for the location estimates of the cursor. In sum, localization responses to unimodally presented visual and auditory stimuli have a Gaussian probability distribution that, after the audiovisual recalibration task, additionally depends on the final shift updates $\Delta_{A_{i}}\left(121\right)$ and $\Delta_{V_{i}}\left(121\right)$.

### Model fitting

All models were fit using a maximum-likelihood procedure. That is, a set of free parameters Θ was chosen to maximize the log likelihood of the data given a model *M*. Our model fitting strategy aimed to reduce the number of free parameters estimated at once. We split the set of free parameters into three subsets Θ_*i*_, *i* = 1, 2, 3, each fit to a subset of the data *X*_*i*_, *i* = 1, 2, 3, and maximized the log-likelihoods of each subset *X*_*i*_ separately,

$$\begin{array}{c} \text{log}\;P\left(X|M,\Theta \right)=\text{log}\;P\left(X_{1}|M,\Theta_{1}\right)+\text{log}\;P\left(X_{2}|M,\Theta_{2}\right)+\text{log}\;P\left(X_{3}|M,\Theta_{3}\right), \end{array}$$ (35)

where *X*_1_, *X*_2_, *X*_3_ ⊂ *X* and Θ_1_, Θ_2_, Θ_3_ ⊂ Θ. The first dataset, *X*_1_, refers to the unimodal spatial-discrimination task, which was used to constrain parameter subset, Θ_1_, that comprised unimodal stimulus reliabilities as well as lapse rates in the different sessions of this task. *X*_2_ refers to the pointing practice task used to estimate parameter subset, Θ_2_, which comprised only the variability in localization responses due to other factors than spatial perception. The third subset comprised data from three tasks, $X_{3}^{1},X_{3}^{2},X_{3}^{3}\subset X_{3}$, the bimodal spatial-discrimination task, and the unimodal spatial-localization task for the pre- and post-recalibration phase, respectively. These three datasets constrained overlapping sets of parameters $\Theta_{3}^{1},\Theta_{3}^{2},\Theta_{3}^{3}\subset \Theta_{3}$. Thus, they were fit jointly. $\Theta_{3}^{1}$ and $\Theta_{3}^{2}$ comprised only localization bias parameters as well as task-specific lapse rates; stimulus reliabilities and response noise parameter estimates were taken from Θ_1_ and Θ_2_. Only $\Theta_{3}^{3}$, the parameter set constrained by participants’ post-recalibration localization responses, included parameters specific to each of the three models of cross-modal recalibration such as the learning rate and common-cause prior.

As outlined before, we did not fit the localization responses from the audiovisual recalibration task, i.e., the build-up of the recalibration effect (Section S13 in S1 Appendix), because the shifts ($\Delta_{A_{i}}\left(t\right)$ and $\Delta_{V_{i}}\left(t\right)$) are serially dependent, that is, the size of the shift in trial *t* depends on the size of the shift in trial *t* − 1. Given that there is no closed-form solution for the causal-inference model, we would have needed to use Monte Carlo simulations to approximate the probability distribution of the location estimates. Yet, the location estimates depend on the serially dependent shifts and consequently the number of necessary samples would have grown exponentially from trial to trial. Thus, it was computationally challenging to estimate the likelihood of the parameters and the model given the data from the audiovisual recalibration task. Instead, we used Monte Carlo simulations to approximate the probability distribution of the shift updates $\Delta_{A_{i}}\left(121\right)$ and $\Delta_{V_{i}}\left(121\right)$ accumulated at the end of the audiovisual recalibration task, i.e., we fitted the final recalibration effect rather than its build-up.

#### Model log-likelihood—Unimodal spatial-discrimination task

In the unimodal spatial-discrimination task (auditory session), participants indicated whether the test stimulus was located to the left, *r*_*A*,*n*(*t*)_ = 0, or to the right of the standard stimulus, *r*_*A*,*n*(*t*)_ = 1. For each such trial, the likelihood of model parameters given the response *r*_*A*,*n*(*t*)_ is

$$\begin{array}{c} P\left(r_{A,n(t)}|M,\Theta_{1}\right)={p_{r_{A,n(t)}=1}}^{r_{A,n(t)}}{\left(1-p_{r_{A,n(t)}=1}\right)}^{1-r_{A,n(t)}}, \end{array}$$ (36)

where $p_{r_{A,n}=1}$ is defined in Eq 23. Thus, the log likelihood given responses across all *T*_1,*A*_ trials is

$$\begin{array}{c} \text{log}\;P\left(X_{1,A}|M,\Theta_{1}\right)=\sum_{t=1}^{T_{1,A}}[r_{A,n(t)}\text{log}\;p_{r_{A,n(t)}=1}+\left(1-r_{A,n(t)}\right)\text{log}\left(1-p_{r_{A,n(t)}=1}\right)]. \end{array}$$ (37)

Analogously,

$$\begin{array}{c} \text{log}\;P\left(X_{1,V_{i}}|M,\Theta_{1}\right)=\sum_{t=1}^{T_{1,V_{i}}}[r_{V_{i},n\left(t\right)}\text{log}p_{r_{V_{i},n\left(t\right)}=1}+\left(1-r_{V_{i},n\left(t\right)}\right)\text{log}\left(1-p_{r_{V_{i},n\left(t\right)}=1}\right)]. \end{array}$$ (38)

The log likelihood across all four sessions is

$$\begin{array}{c} \text{log}\;P\left(X_{1}|M,\Theta_{1}\right)=\text{log}\;P\left(X_{1,A}|M,\Theta_{1}\right)+\sum_{i=1}^{3}\text{log}\;P\left(X_{1,V_{i}}|M,\Theta_{1}\right). \end{array}$$ (39)

The set of free parameters that were constrained by the binary responses in this task is $\Theta_{1}=\left\{\sqrt{2}\sigma_{A}^{\prime }/a_{A},\sigma_{V_{1}}^{\prime },\sigma_{V_{2}}^{\prime },\sigma_{V_{3}}^{\prime },\lambda_{A},\lambda_{V_{1}},\lambda_{V_{2}},\lambda_{V_{3}}\right\}$.

#### Model log-likelihood—Pointing practice task

For each trial, the likelihood of the model parameters given a visual stimulus at location *s*_*V*,*o*(*t*)_ and a subsequent response (cursor setting) *r*_*V*,*o*(*t*)_ is $P\left(r_{V,o(t)}|M,\Theta_{2}\right)=\varphi \left(r_{V,o(t)};s_{V,o(t)},\sigma_{r}^{2}\right)$ where *φ* refers to the Gaussian probability density. The only free parameter that was constrained by this task is Θ_2_ = {*σ*_*r*_}. The maximum-likelihood estimate of *σ*_*r*_ is

$$\begin{array}{c} \sigma_{r}=\sqrt{\frac{\sum_{t=1}^{T_{2}}{\left(s_{V,o(t)}-r_{V,o(t)}\right)}^{2}}{T_{2}}}, \end{array}$$ (40)

where *T*_2_ and the sum do not include outlier trials.

#### Model log-likelihood—Bimodal spatial-discrimination task

In the bimodal spatial-discrimination task, for each trial *t*, participants indicated whether the visual test stimulus at location $s_{V_{1},n(t)}$ was located to the left, *r*_*l*(*t*),*n*(*t*)_ = 0, or to the right, *r*_*l*(*t*),*n*(*t*)_ = 1, of the auditory standard stimulus presented at *s*_*A*,*l*(*t*)_. For each such trial, the likelihood of model parameters given the response *r*_*l*(*t*),*n*(*t*)_ is

$$\begin{array}{c} P\left(r_{l(t),n(t)}|M,\Theta_{3}^{1}\right)={p_{r_{l(t),n(t)}=1}}^{r_{l(t),n(t)}}{\left(1-p_{r_{l(t),n(t)}=1}\right)}^{1-r_{l(t),n(t)}}, \end{array}$$ (41)

where $p_{r_{l,n}=1}$ is defined in Eq 31. Thus, the log likelihood given the responses across all $T_{3}^{1}$ trials is

$$\begin{array}{c} \text{log}\;P\left(X_{3}^{1}|M,\Theta_{3}^{1}\right)=\sum_{t=1}^{T_{3}^{1}}[r_{l(t),n(t)}\text{log}p_{r_{l(t),n(t)}=1}+\left(1-r_{l(t),n(t)}\right)\text{log}\left(1-p_{r_{l(t),n(t)}=1}\right)]. \end{array}$$ (42)

$p_{r_{l,n}=1}$ is a function of *p*_*l*(*t*),*n*(*t*)_, which in turn depends on the bias parameters *a*_*A*_ and *b*_*A*_, the parameters of the supra-modal prior over locations $\mu_{P}^{\prime }$ and $\sigma_{P}^{\prime }$, as well as the measurement variances $\sigma_{A}^{\prime }$ and $\sigma_{V_{i}}^{\prime }$ (see Eqs 24–27). Fitting both the bias parameters and the supra-modal prior at once was impossible as they effectively traded off. Thus, we implemented a non-informative supra-modal prior over stimulus locations by setting $\sigma_{P}^{\prime }$ to 100 and $\mu_{P}^{\prime }$ to 0. $a\sigma_{A}^{\prime }$, $\sigma_{V_{1}}^{\prime }$, $\sigma_{V_{2}}^{\prime }$, and $\sigma_{V_{3}}^{\prime }$ were estimated based on the forced-choice responses from the unimodal spatial-discrimination task. The final set of free parameters that were constrained by the binary responses in this task was $\Theta_{3}^{1}=\left\{a_{A},b_{A},\lambda_{VA}\right\}$. The bias parameters, *a*_*A*_ and *b*_*A*_, were jointly estimated using the data from this task as well as pre- and post-recalibration responses from the unimodal localization task.

#### Model log-likelihood—Unimodal localization task—Pre-recalibration phase

In this task, each localization results in cursor location settings *r*_*A*,*i*,*j*,*l*(*t*)_ and $r_{V_{i},j,l(t)}$ on trial *t* of session (*i*, *j*) where *i* indicates the visual-reliability condition and *j* the recalibration direction in the subsequent recalibration phase. The localization responses from this task were modeled as Gaussian-distributed. From these distributions, we can compute the likelihood of a model *M* and the parameter set $\Theta_{3}^{2}$ as the Gaussian probability density function in Eq 33 evaluated at the observed localization responses *r*_*A*,*i*,*j*,*l*(*t*)_ and $r_{V_{i},j,l(t)}$:

$$\begin{array}{c} \begin{array}{c} P\left(r_{A,i,j,l(t)}|M,\Theta_{3}^{2}\right)={\left(2\pi (\sigma_{{\hat{s}}_{A}^{\prime }}^{2}+\sigma_{r}^{2})\right)}^{-\frac{1}{2}}\;\text{exp}[-\frac{{\left(r_{A,i,j,l(t)}-\mu_{{\hat{s}}_{A,i,j,l(t)}^{\prime }}\right)}^{2}}{2(\sigma_{{\hat{s}}_{A}^{\prime }}^{2}+\sigma_{r}^{2})}]\\ P\left(r_{V_{i},j,l\left(t\right)}|M,\Theta_{3}^{2}\right)={\left(2\pi (\sigma_{{\hat{s}}_{V_{i}}^{\prime }}^{2}+\sigma_{r}^{2})\right)}^{-\frac{1}{2}}\;\text{exp}[-\frac{{\left(r_{V_{i},j,l\left(t\right)}-\mu_{{\hat{s}}_{V_{i},j,l\left(t\right)}^{\prime }}\right)}^{2}}{2(\sigma_{{\hat{s}}_{V_{i}}^{\prime }}^{2}+\sigma_{r}^{2})}]. \end{array} \end{array}$$ (43)

The log likelihood is the sum of the log likelihoods across the trials of all six sessions:

$$\begin{array}{c} \begin{array}{cc} & \text{log}\;P(X_{3}^{2}|M,\Theta_{3}^{2})\\ & \;=\sum_{i=1}^{3}\sum_{j=1}^{2}[\sum_{t_{A}=1}^{T_{3,A,i,j}^{2}}\text{log}\;P\left(r_{A,i,j,l(t_{A})}|M,\Theta_{3}^{2}\right)+\sum_{t_{V_{i}}=1}^{T_{3,V_{i},j}^{2}}\text{log}\;P\left(r_{V_{i},j,l\left(t_{V_{i}}\right)}|M,\Theta_{3}^{2}\right)]\\ & \;=\sum_{i=1}^{3}\sum_{j=1}^{2}[-\frac{T_{3,A,i,j}^{2}}{2}\text{log}\left(2\pi \left(\sigma_{{\hat{s}}_{A}^{\prime }}^{2}+\sigma_{r}^{2}\right)\right)-\frac{T_{3,V_{i},j}^{2}}{2}\text{log}\left(2\pi \left(\sigma_{{\hat{s}}_{V_{i}}^{\prime }}^{2}+\sigma_{r}^{2}\right)\right)\\ & \;-\frac{1}{2(\sigma_{{\hat{s}}_{A}^{\prime }}^{2}+\sigma_{r}^{2})}\sum_{t_{A}=1}^{T_{3,A,i,j}^{2}}{\left(r_{A,i,j,l(t_{A})}-\mu_{{\hat{s}}_{A,i,j,l(t_{A})}}\right)}^{2}\\ & \;-\frac{1}{2(\sigma_{{\hat{s}}_{V_{i}}^{\prime }}^{2}+\sigma_{r}^{2})}\sum_{t_{V_{i}}=1}^{T_{3,V_{i},j}^{2}}{\left(r_{V_{i},j,l\left(t_{V_{i}}\right)}-\mu_{{\hat{s}}_{V_{i},j,l\left(t_{V_{i}}\right)}}\right)}^{2}]. \end{array} \end{array}$$ (44)

The log-likelihood depends on $\mu_{{\hat{s}}_{A,i,j,l(t_{A})}}$ and $\mu_{{\hat{s}}_{V_{i},j,l\left(t_{V_{i}}\right)}}$, which in turn depend on the bias parameters *a*_*A*_ and *b*_*A*_, the parameters of the supra-modal prior $\mu_{P}^{\prime }$ and $\sigma_{P}^{\prime }$, as well as the measurement variances $\sigma_{A}^{\prime }$ and $\sigma_{V_{i}}^{\prime }$ (see Eq 34), and the response noise *σ*_*r*_. We chose a flat prior over stimulus locations, the (scaled) measurement variances ($\sqrt{2}\sigma_{A}^{\prime }/a_{A}$ and $\sigma_{V_{i}}^{\prime }$) were estimated based on the unimodal spatial-discrimination task, and *σ*_*r*_ was estimated based on the pointing practice task. Consequently, the actual set of parameters constrained by localization responses from the pre-recalibration task was $\Theta_{3}^{2}=\left\{a_{A},b_{A}\right\}$. Here, the values of the *T* variables and the sums do not include outlier trials.

#### Model log-likelihood—Unimodal localization task—Post-recalibration phase

Localization responses in the post-recalibration phase additionally depend on the updates for the visual and auditory shifts accumulated after 120 trials during the recalibration phase, $\Delta_{A_{i}}\left(121\right)$ and $\Delta_{V_{i}}\left(121\right)$ (Eq 34). Since these accumulated shift updates are not accessible to the experimenter, we marginalized over these shift updates to calculate the log-likelihood. For each of the six experimental sessions (*i*, *j*), the log likelihood of a model *M* and its parameter set $\Theta_{3}^{3}$ is the integral over $\Delta_{A_{i}}$ and $\Delta_{V_{i}}$ of the likelihood of the final shift updates given the observed data $X_{3,i,j}^{3}$, the model *M*, and the parameter set $\Theta_{3}^{3}$, $P(X_{3,i,j}^{3}|\Delta_{A_{i},j},\Delta_{V_{i},j},M,\Theta_{3}^{3})$, multiplied by the joint probability of the auditory and visual shift updates, $P(\Delta_{A_{i},j},\Delta_{V_{i},j}|M,\Theta_{3}^{3})$, summed across all six sessions

$$\begin{array}{c} \begin{array}{cc} & \text{log}\;P(X_{3}^{3}|M,\Theta_{3}^{3})=\\ & \sum_{i=1}^{3}\sum_{j=1}^{2}\text{log}\;(\;\int \int \;P\left(X_{3,i,j}^{3}|\Delta_{A_{i},j},\Delta_{V_{i},j},M,\Theta_{3}^{3}\right)P\left(\Delta_{A_{i},j},\Delta_{V_{i},j}|M,\Theta_{3}^{3}\right)d\Delta_{A_{i},j}d\Delta_{V_{i},j}). \end{array} \end{array}$$ (45)

We will describe in the following sections how the joint probability $P(\Delta_{A_{i},j},\Delta_{V_{i},j}|M,\Theta_{3}^{3})$ and the log-likelihood $\text{log}\;P(X_{3}^{3}|M,\Theta_{3}^{3})$ were derived for each of the three models of cross-modal recalibration.

**Reliability-based model of cross-modal recalibration**. In this model, auditory and visual shift updates have a constant ratio of $\Delta_{A_{i}}\left(t\right)/\Delta_{V_{i}}\left(t\right)=-{\sigma_{AV,A}^{\prime }}^{2}/{\sigma_{AV,V_{i}}^{\prime }}^{2}$, the ratio of the measurement noise variances. Therefore, $\Delta_{A_{i}}\left(t\right)$ can be rewritten as $\left(-{\sigma_{AV,A}^{\prime }}^{2}/{\sigma_{AV,V_{i}}^{\prime }}^{2}\right)\Delta_{V_{i}}\left(t\right)$, and we can express the likelihood given a single auditory localization response $r_{A_{i},j,l(t)}$ as

$$\begin{array}{c} P\left(r_{A_{i},j,l\left(t\right)}|\Delta_{V_{i},j},M_{RB},\Theta_{3,RB}^{3}\right)={\left(2\pi (\sigma_{{\hat{s}}_{A_{i}}^{\prime }}^{2}+\sigma_{r}^{2})\right)}^{-\frac{1}{2}}\text{exp}[-\frac{{\left(r_{A_{i},j,l\left(t\right)}-\mu_{{\hat{s}}_{A_{i},j,l\left(t\right)}^{\prime }}\right)}^{2}}{2(\sigma_{{\hat{s}}_{A_{i}}^{\prime }}^{2}+\sigma_{r}^{2})}], \end{array}$$ (46)

where

$$\begin{array}{c} \mu_{{\hat{s}}_{A_{i},l\left(t\right)}^{\prime }}=\frac{{\sigma_{AV,A}^{\prime }}^{-2}\left(a_{A}s_{A,l(t)}+b_{A}-\frac{{\sigma_{AV,A}^{\prime }}^{2}}{{\sigma_{AV,V_{i}}^{\prime }}^{2}}\Delta_{V_{i},j}\left(121\right)\right)+{\sigma_{P}^{\prime }}^{-2}\mu_{P}^{\prime }}{{\sigma_{AV,A}^{\prime }}^{-2}+{\sigma_{P}^{\prime }}^{-2}}. \end{array}$$ (47)

The visual response likelihoods and means are defined analogously (Eq 34). Thus, the joint likelihood $P(X_{3,i,j}^{3}|\Delta_{A_{i},j},\Delta_{V_{i},j},M_{RB},\Theta_{3,RB}^{3})$ can be written as $P(X_{3,i,j}^{3}|\Delta_{V_{i},j},M_{RB},$ $\Theta_{3,RB}^{3})$. Given that the likelihood depends only on $\Delta_{V_{i},j}$, we only need to integrate over $\Delta_{V_{i},j}$ and the log likelihood simplifies to

$$\begin{array}{c} \begin{array}{cc} & \text{log}\;P(X_{3}^{3}|M_{RB},\Theta_{3,RB}^{3})=\\ & \sum_{i=1}^{3}\sum_{j=1}^{2}\text{log}\;(\int P\left(X_{3,i,j}^{3}|\Delta_{V_{i},j},M_{RB},\Theta_{3,RB}^{3}\right)P\left(\Delta_{V_{i},j}|M_{RB},\Theta_{3,RB}^{3}\right)d\Delta_{V_{i},j}). \end{array} \end{array}$$ (48)

The shift updates $\Delta_{V_{i},j}$ are stochastic because the visual and auditory measurements in each trial of the audiovisual recalibration task are stochastic. We cannot derive their probability distribution $P(\Delta_{V_{i},j}|M_{RB},\Theta_{3,RB}^{3})$ in closed form. Instead, we used Monte Carlo simulation to approximate this probability distribution. Given the reliability-based model, for each candidate set of parameters $\Theta_{3,RB}^{3}$, visual-reliability condition *i*, and recalibration direction *j*, we simulated 120 recalibration trials analogous to the audiovisual recalibration task. We repeated this simulation 1,000 times, resulting in a sample of 1,000 shift updates ($\Delta_{V_{i}}$) and checked whether the distribution of the 1,000 samples was well fit by a Gaussian with mean and standard deviation equal to the corresponding empirical parameters of the sampled distribution. To do so, we binned the simulated shift updates into 100 bins of equal size and computed the correlation between the observed and predicted number of samples per bin. The resulting value of *R*^2^ was greater than 0.925 in all cases (Section S8 in S1 Appendix). The approximated probability distribution of the shift updates is denoted as $\tilde{P}\left(\Delta_{V_{i},j}|M_{RB},\Theta_{3,RB}^{3}\right)$.

We approximated the integral in Eq 48 by numerical integration over a region discretized into 100 bins. To ensure that we include enough of the tails of the probability distribution of the shift updates, we set the integration region to be three times larger than the range of the $\Delta_{V_{i}}$ samples, and centered the integration region on that range. Thus, the lower bound, *lb*, is defined as *lb* = Δ_*min*_ − (Δ_*max*_ − Δ_*min*_) and the upper bound is *ub* = Δ_*max*_ + (Δ_*max*_ − Δ_*min*_). The numerical integration region was derived separately for each session. The log likelihood is:

$$\begin{array}{c} \begin{array}{cc} & \text{log}\;P(X_{3}^{3}|M_{RB},\Theta_{3,RB}^{3})\\ & =\sum_{i=1}^{3}\sum_{j=1}^{2}\text{log}(\int P\left(X_{3,i,j}^{3}|\Delta_{V_{i},j},M_{RB},\Theta_{3,RB}^{3}\right)P\left(\Delta_{V_{i},j}|M_{RB},\Theta_{3,RB}^{3}\right)d\Delta_{V_{i},j})\\ & \approx \sum_{i=1}^{3}\sum_{j=1}^{2}\text{log}(\int_{lb_{V_{i},j}}^{ub_{V_{i},j}}P\left(X_{3,i,j}^{3}|\Delta_{V_{i},j},M_{RB},\Theta_{3,RB}^{3}\right)\tilde{P}\left(\Delta_{V_{i},j}|M_{RB},\Theta_{3,RB}^{3}\right)d\Delta_{V_{i},j})\\ & \approx \sum_{i=1}^{3}\sum_{j=1}^{2}\text{log}(\frac{ub_{V_{i},j}-lb_{V_{i},j}}{100}\sum_{k_{V}=1}^{100}P\left(X_{3,i,j}^{3}|\Delta_{V_{i},j}\left(k_{V}\right),M_{RB},\Theta_{3,RB}^{3}\right)\times \\ & \;\;\tilde{P}\left(\Delta_{V_{i},j}\left(k_{V}\right)|M_{RB},\Theta_{3,RB}^{3}\right)), \end{array} \end{array}$$ (49)

where

$$\begin{array}{c} \begin{array}{cc} & P(X_{3,i,j}^{3}|\Delta_{V_{i},j}\left(k_{V}\right),M_{RB},\Theta_{3,RB}^{3})\\ & =\;\;\prod_{t_{A}=1}^{T_{3,A,i,j}^{3}}\;\;P\left(r_{A_{i},j,l\left(t_{A}\right)}|\Delta_{V_{i},j}\left(k_{V}\right),M_{RB},\Theta_{3,RB}^{3}\right)\;\;\prod_{t_{V_{i}}=1}^{T_{3,V_{i},j}^{3}}\;\;P\left(r_{V_{i},j,l\left(t_{V_{i}}\right)}|\Delta_{V_{i},j}\left(k_{V}\right),M_{RB},\Theta_{3,RB}^{3}\right)\\ & ={\left(2\pi (\sigma_{{\hat{s}}_{A_{i}}}^{2}+\sigma_{r}^{2})\right)}^{-\frac{T_{3,A,i,j}^{3}}{2}}\text{exp}[-{\left(2(\sigma_{{\hat{s}}_{A_{i}}}^{2}+\sigma_{r}^{2})\right)}^{-1}\sum_{t_{A}=1}^{T_{3,A,i,j}^{3}}{\left(r_{A_{i},j,l\left(t_{A}\right)}-\mu_{{\hat{s}}_{A_{i},l\left(t_{A}\right)}^{\prime }}\right)}^{2}]\times \\ & \;{\left(2\pi (\sigma_{{\hat{s}}_{V_{i}}}^{2}+\sigma_{r}^{2})\right)}^{-\frac{T_{3,V_{i},j}^{3}}{2}}\text{exp}[-{\left(2(\sigma_{{\hat{s}}_{V_{i}}}^{2}+\sigma_{r}^{2})\right)}^{-1}\sum_{t_{V_{i}}=1}^{T_{3,V_{i},j}^{3}}{\left(r_{V_{i},j,l\left(t_{V_{i}}\right)}-\mu_{{\hat{s}}_{V_{i},l\left(t_{V_{i}}\right)}^{\prime }}\right)}^{2}], \end{array} \end{array}$$ (50)

with $\mu_{{\hat{s}}_{A,l(t)}^{\prime }}$ defined in Eq 47 and $\mu_{{\hat{s}}_{V_{i},l\left(t\right)}^{\prime }}$ defined in Eq 34. $\mu_{{\hat{s}}_{A_{i},l\left(t\right)}^{\prime }}$ and $\mu_{{\hat{s}}_{V_{i},l\left(t\right)}^{\prime }}$ depend on the bias parameters *a*_*A*_ and *b*_*A*_, as well as on $\Delta_{V_{i},j}$, which depends on the measurement variances $\sigma_{AV,A}^{\prime }$ and $\sigma_{AV,V_{i}}^{\prime }$ given bimodal presentation and the common learning rate *α*. Note that $\sigma_{AV,A}^{\prime }$ and $\sigma_{AV,V_{i}}^{\prime }$ are not directly constrained by data from bimodal trials (because these trials were not included in the model fitting), but estimated based on their influence on the shift updates. Specifically, $\sigma_{AV,A}^{\prime }$ and $\sigma_{AV,V_{i}}^{\prime }$ affect the spread of the measurements, and as a consequence they influence the width of the predicted probability distribution of measurement-shift updates, which in turn affect the log likelihood of the model. The set of free parameters for this model is $\Theta_{3,RB}^{3}=\left\{a_{A},b_{A},\sigma_{AV,A}^{\prime },\sigma_{AV,V_{1}}^{\prime },\sigma_{AV,V_{2}}^{\prime },\sigma_{AV,V_{3}}^{\prime },\alpha \right\}$. $\sigma_{AV,V_{i}}^{\prime }$ was constrained to be a non-decreasing function of visual-reliability condition *i*, and $\sigma_{AV,A}^{\prime }$ and $\sigma_{AV,V_{i}}^{\prime }$ were constrained to be no greater than five times the average values of $\sigma_{A}^{\prime }$ and $\sigma_{V_{i}}^{\prime }$ across participants (Section S14 in S1 Appendix). The values of the *T* variables and the sums do not include outlier trials.

**Log-likelihood—fixed-ratio model of cross-modal recalibration**. In this model, auditory and visual shift updates have a fixed ratio of $\Delta_{A_{i}}\left(t\right)/\Delta_{V_{i}}\left(t\right)=-\alpha_{A}/\alpha_{V}$ (Section S15 in S1 Appendix). Thus, we can express the likelihood for the fixed-ratio model and parameter set $\Theta_{3,FR}^{3}$ given an auditory localization response $r_{A_{i},j,l(t)}$ in a similar form to Eq 47:

$$\begin{array}{c} \mu_{{\hat{s}}_{A_{i},l\left(t\right)}^{\prime }}=\frac{{\sigma_{AV,A}^{\prime }}^{-2}\left(a_{A}s_{A,l(t)}+b_{A}-\frac{\alpha_{A}}{\alpha_{V}}\Delta_{V_{i},j}\left(121\right)\right)+{\sigma_{P}^{\prime }}^{-2}\mu_{P}^{\prime }}{{\sigma_{AV,A}^{\prime }}^{-2}+\sigma_{P}^{-2}}. \end{array}$$ (51)

The approximation $\tilde{P}\left(\Delta_{V_{i},j}|M_{FR},\Theta_{3,FR}^{3}\right)$ was generated in the same way as for the reliability-based model. The set of free parameters for this model is $\Theta_{3,FR}^{3}=\{a_{A},b_{A},\sigma_{AV,A}^{\prime },\sigma_{AV,V_{1}}^{\prime },\sigma_{AV,V_{2}}^{\prime },\sigma_{AV,V_{3}}^{\prime },\alpha_{A},\alpha_{V}\}$. Note that even though the shift updates in the fixed-ratio model do not depend on the stimulus reliabilities, the log-likelihood does due to the influence of stimulus reliability on the estimates in the localization task (Eq 51) and due to the influence of the spread of the simulated measurements on the spread of the estimated distribution of $\tilde{P}\left(\Delta_{V_{i},j}|M_{FR},\Theta_{3,FR}^{3}\right)$. As in the reliability-based model, $\sigma_{AV,V_{i}}^{\prime }$ was constrained to be a non-decreasing function of visual-reliability condition *i*, and $\sigma_{AV,A}^{\prime }$ and $\sigma_{AV,V_{i}}^{\prime }$ were constrained to be no greater than five times the average values of $\sigma_{A}^{\prime }$ and $\sigma_{V_{i}}^{\prime }$ across participants.

**Log-likelihood—causal-inference model of cross-modal recalibration**. For this model, the joint likelihood $P(X_{3,i,j}^{3}|\Delta_{A_{i},j},\Delta_{V_{i},j},M_{CI},\Theta_{3,CI}^{3})$ was truly two-dimensional. Thus, we approximated the joint probability of the auditory and visual shift updates, $P(\Delta_{A_{i},j},\Delta_{V_{i},j}|M,\Theta_{3,CI}^{3})$ by drawing 1000 samples of shift-update pairs and compared the set of sample pairs to a 2-d Gaussian with the sample mean and covariance as parameters. We again tested whether the two-dimensional Gaussian distribution provided a good fit to the simulated density (defined as *R*^2^ > 0.925). If the Gaussian fit was poor, we used a kernel density estimate (Gaussian kernel smoother with *σ* chosen automatically) of the distribution based on the 2-d density of the samples [77, 78]. Overall, the simulated auditory and visual shift updates were very well fit by a bivariate Gaussian, and we rarely used a kernel density estimate (Section S8 in S1 Appendix). We additionally used simulations to verify that our estimates of the partial model log-likelihood ($\text{log}\;P(X_{3}^{3}|M_{CI},\Theta_{3,CI}^{3})$) had reasonably small bias (Section S8 in S1 Appendix).

For the causal-inference model, we approximate the log likelihood by numerical integration over a 2-dimensional region of Δ_*A*_, Δ_*V*_ space discretized into 100x100 bins. The upper and lower bounds were determined for both dimensions in the same way as before. The log likelihood is:

$$\begin{array}{cc} & \text{log}\;P(X_{3}^{3}|M_{CI},\Theta_{3,CI}^{3})\\ & \;=\sum_{i=1}^{3}\sum_{j=1}^{2}\text{log}(\;\int \int \;P\left(X_{3,i,j}^{3}|\Delta_{A_{i},j},\Delta_{V_{i},j},M_{CI},\Theta_{3,CI}^{3}\right)\times \\ & \;P\left(\Delta_{A_{i},j},\Delta_{V_{i},j}|M_{CI},\Theta_{3,CI}^{3}\right)d\Delta_{A_{i},j}d\Delta_{V_{i},j})\\ & \;\approx \sum_{i=1}^{3}\sum_{j=1}^{2}\text{log}[\int_{lb_{V_{i},j}}^{ub_{V_{i},j}}\int_{lb_{A_{i},j}}^{ub_{A_{i},j}}P\left(X_{3,i,j}^{3}|\Delta_{A_{i},j},\Delta_{V_{i},j},M_{CI},\Theta_{3,CI}^{3}\right)\times \\ & \;\tilde{P}\left(\Delta_{A_{i},j},\Delta_{V_{i},j}|M_{CI},\Theta_{3,CI}^{3}\right)d\Delta_{A_{i},j}d\Delta_{V_{i},j}]\\ & \;\approx \sum_{i=1}^{3}\sum_{j=1}^{2}\text{log}[\frac{ub_{V_{i},j}-lb_{V_{i},j}}{100}\frac{ub_{A_{i},j}-lb_{A_{i},j}}{100}\times \\ & \;\sum_{k_{V}=1}^{100}\sum_{k_{A}=1}^{100}P\left(X_{3,i,j}^{3}|\Delta_{A_{i},j}\left(k_{A}\right),\Delta_{V_{i},j}\left(k_{V}\right),M_{CI},\Theta_{3,CI}^{3}\right)\times \\ & \;P(\Delta_{A_{i},j}\left(k_{A}\right),\Delta_{V_{i},j}\left(k_{V}\right)|M_{CI},\Theta_{3,CI}^{3})], \end{array}$$ (52)

where $P(X_{3,i,j}^{3}|\Delta_{A_{i},j}\left(k_{A}\right),\Delta_{V_{i},j}\left(k_{V}\right),M_{CI},\Theta_{3,CI}^{3})$ is defined analogously to the reliability-based model (see Eq 50) with the exception that $\mu_{{\hat{s}}_{A,l(t)}^{\prime }}$ and $\mu_{{\hat{s}}_{V_{i},l\left(t\right)}^{\prime }}$ are defined in Eq 34. The set of free parameters used to fit the causal-inference model to the localization responses in the post-recalibration task is $\Theta_{3,CI}^{3}=\left\{a_{A},b_{A},\sigma_{AV,A}^{\prime },\sigma_{AV,V_{1}}^{\prime },\sigma_{AV,V_{2}}^{\prime },\sigma_{AV,V_{3}}^{\prime },\alpha_{A},\alpha_{V},p_{C=1}\right\}$ or $\Theta_{3,CI_{\alpha_{V}=\alpha_{A}}}^{3}=\left\{a_{A},b_{A},\sigma_{AV,A}^{\prime },\sigma_{AV,V_{1}}^{\prime },\sigma_{AV,V_{2}}^{\prime },\sigma_{AV,V_{3}}^{\prime },\alpha ,p_{C=1}\right\}$.

#### Parameter estimation

For each model, we approximated the set of parameters Θ_1_ and Θ_2_ that maximized the likelihood using the MATLAB function fmincon and Python SciPy.optimize [79], and approximated Θ_3_ using the BADS toolbox [80]. To deal with the possibility that the returned parameter values might correspond to a local minimum, we ran BADS multiple times with different starting points, randomly chosen from a *D*-dimensional grid, where *D* is the number of free parameters in Θ_3_ (see Table 1 for a summary of the free parameters for each model) and with three evenly spaced values chosen for each dimension. The final parameter estimates were those with the maximum likelihood across all runs of the fitting procedure.

**Table 1:** Summary of model parameters in Θ_3_.

Θ	Meaning	RB	FR	CI	${\text{CI}}_{\alpha_{\text{V}}=\alpha_{\text{A}}}$
$\sigma_{AV,A}^{\prime }$	Measurement noise variance for the auditory stimulus in bimodal trials	✓	✓	✓	✓
$\sigma_{AV,V_{i}}^{\prime }$	Measurement noise variance for the visual stimulus in bimodal trials	✓	✓	✓	✓
$\mu_{P}^{\prime }$	The mean of the supra- modal prior distribution	-	-	-	-
$\sigma_{P}^{\prime }$	The standard deviation of the supra- modal prior	-	-	-	-
*a*_*A*_, *b*_*A*_	The slope and the intercept of the linear function that captures biases in auditory measurements	✓	✓	✓	✓
*P*(*C* = 1)	Prior probability of a common cause	-	-	✓	✓
*α*_*A*_, *α*_*V*_	Modality-specific learning rate	-	✓	✓	-
*α*	Common learning rate	✓	-	-	✓
λ_*AV*_	Lapse rate for the bimodal spatial-discrimination task	✓	✓	✓	✓
	**Number of total parameters**:	8	9	10	9

#### Model comparison

To compare model performance quantitatively, we computed the Akaike information criterion (AIC) for all four models [64] and calculated relative model-comparison scores, Δ_AIC_, which relate the AIC value of the best-fit model to that of each of the other models (a higher Δ_AIC_ value indicates stronger evidence for the best-fit model). Models with 0 < Δ_AIC_ < 2 are weakly supported; models with 4 < Δ_AIC_ < 7 have considerably less support; models with Δ_AIC_ > 10 have essentially no support [81].

## Supporting information

S1 Appendix Appendix. Supplemental control experiments, analyses and figures.
(PDF)
