# Supplementary material for: Causal inference regulates audiovisual spatial recalibration via its influence on audiovisual perception
Source: PLoS Comput Biol. 2021 Nov 15;17(11):e1008877. doi: 10.1371/journal.pcbi.1008877 (PMC8629398; doi:10.1371/journal.pcbi.1008877)
Supplement: S1 Appendix — Supplemental control experiments, analyses and figures. (PDF) [file pcbi.1008877.s001.pdf]

S1 Appendix

S1. Statistical Analysis 2

S2. Modeling of the Localization Noise That is Unrelated to Spatial Perception as Measured in the Pointing Practice Task 4

S3. Model Predictions by the Causal-inference Model with a Supra-Modal Learning Rate and Modality-Specific Learning Rates 6

S4. Model Predictions by the Reliability-Based Model 8

S5. Model Predictions by the Fixed-Ratio Model 9

S6. Model Predictions on the Biases in Auditory Spatial Perception Relative to Visual Spatial Perception 10

S7. Biases in Visual Spatial Localization 11

S8. Testing the Biases of the Log Likelihood of Models and Parameters as a Result of Fitting a Univariate/Bivariate Gaussian or Applying a Kernel Smoothing Function to the Visual and Auditory Shifts 14

S9. Trade-Offs Between Free Parameters 20

S10. Excluding Outlier Participants 22

S11. Testing the Effectiveness of Playing the Masking Sound and Moving the Speaker to a Stopover Position in Eliminating Location Cues 24

S12. Examination of the recalibration gain over the course of the post-recalibration task 26

S13. Qualitative predictions for audiovisual localization responses during the audiovisual recalibration phase 27

S14. Issues Regarding the Estimation of the Reliability Under Bimodal Stimulus Presentation Given the Fixed-Ratio and the Reliability-Based Model 28

S15. Proportionality of Auditory and Visual Measurement-Shifts in the Reliability-Based and the Fixed-Ratio Models 30

Reference 31

S1. Statistical Analysis

S1.1. Cue Reliability

A one-way repeated-measures ANOVA on the visual JNDs indicated a significant main effect of visual reliability ($F(2,10) = 15.30$, $p < 0.001$, $\eta_G^2 = 0.637$). Including the excluded participant does not change the significance of the main effect ($F(2,12) = 20.78$, $p < 0.001$, $\eta_G^2 = 0.666$). Two-tailed, paired *t*-tests comparing the JNDs of each of those three visual conditions with that of the auditory condition showed that the JND of the visual stimulus was significantly smaller than that of the auditory stimulus for the high-reliability condition ($t(5) = -3.19$, $p = 0.024$, $d = -1.98$), not significantly different for the medium-reliability condition ($t(5) = 0.67$, $p = 0.533$, $d = 0.35$), and significantly larger for the low-reliability condition ($t(5) = 3.97$, $p = 0.011$, $d = 1.50$). Including the excluded participant does not change the results (high-reliability condition: $t(6) = -3.89$, $p = 0.008$, $d = -2.24$; medium-reliability condition: $t(6) = 0.10$, $p = 0.921$, $d = 0.05$; low-reliability condition: $t(6) = 4.58$, $p = 0.004$, $d = 1.60$). Two-tailed, paired *t*-tests comparing across visual JNDs showed that the JND of the visual stimulus in the high-reliability condition is significantly smaller than that in the medium-reliability condition ($t(5) = -7.57$, $p < 0.001$, $d = -3.27$) and than that in the low-reliability condition ($t(5) = -4.17$, $p = 0.009$, $d = -2.49$), and the JND of the visual stimulus in the medium-reliability condition is significantly smaller than that in the low-reliability condition ($t(5) = -2.94$, $p = 0.032$, $d = -1.41$). Including the excluded participant does not change the results (high- vs. medium-reliability condition: $t(6) = -6.26$, $p < 0.001$, $d = -2.71$; high- vs. low-reliability condition: $t(6) = -4.95$, $p = 0.003$, $d = -2.74$; medium- vs. low-reliability condition: $t(6) = -3.62$, $p = 0.011$, $d = -1.62$).

S1.2. Recalibration

We examined whether the amount of visual recalibration by audition changed as a function of adaptation direction and visual reliability by conducting a repeated-measures two-way ANOVA on visual recalibration gain (the outlier participant was not included in this statistical analysis). The amount of visual recalibration did not differ significantly between the

visual-left-of-auditory and the visual-right-of-auditory conditions ($F(1{,}5) < 1$, $\eta_G^2 = 0.001$) and between visual-reliability conditions ($F(2{,}10) < 1$, $\eta_G^2 = 0.047$). Also, there was no significant interaction between the two factors ($F(2{,}10) < 1$, $\eta_G^2 = 0.004$). The results were similar for auditory recalibration by vision: no significant main effect of adaptation direction ($F(1{,}5) = 2.944$, $p = 0.147$, $\eta_G^2 = 0.091$), no significant main effect of visual reliability ($F(2{,}10) < 1$, $\eta_G^2 = 0.039$), and no significant interaction between these two factors ($F(2{,}10) < 1$, $\eta_G^2 = 0.021$).

S2. Modeling of the Localization Noise That is Unrelated to Spatial Perception as Measured in the Pointing Practice Task

All participants completed a block of pointing practice (see procedure in **Fig. S1A**) before the first session of the recalibration experiment to get familiarized with using the pointing device and the foot pedal. With all the localization responses from this task, we were able to measure the setting noise plus participants' memory/motor noise, denoted as σ_r.

For each participant, we first identified outlier localization responses for each stimulus location s_l ($s_l \in \{-17.5°, -12.5°, \ldots, 17.5°\}$ and $l = 1,2,...,8$) by z-transforming the responses, and excluded those with z-score above 3 or below -3 from further analysis. We considered the following two models for the localization responses: (1) **M0**: the localization responses were unbiased and participants' memory/motor noise plus the setting noise was constant across those stimulus locations, and (2) **M1**: the localization responses were biased and both the bias and the noise are linear functions of stimulus eccentricity. More specifically, we assumed that localization responses at each location l are Gaussian, distributed as $\mathcal{N}(\mu_l, \sigma_l)$. In **M0**, μ_l equals the physical location of the visual stimulus, i.e., $\mu_l = s_l$, and σ_l is constant across locations, i.e., $\sigma_l = \sigma_r$ for $l = 1,2,...,8$. In contrast, for **M1** the mean of the Gaussian distribution is a linear function of stimulus location, i.e., $\mu_l = b_\mu + c_\mu s_l$, where b_μ represents a constant bias term and c_μ represents a proportional bias term. The variance of the Gaussian distribution is a linear function of the absolute distance of the stimulus from the central fixation $|s_l|$, i.e., $\sigma_l^2 = \sigma_r^2 + c_{\sigma^2}|s_l|$.

To compare model performance, we performed leave-one-out cross-validation (LOOCV) for each model. More specifically, we fit it to a training set, R_{-r_i}, which included all the non-outlier localization responses except $r_i \in R$, where i indexes the trial number ($i = 1,2,...,N$). Each model was fit by a maximum-likelihood procedure, i.e., maximizing $\log p(R_{-r_i} | \Theta, M)$. We denoted the best-fit model parameters as $\hat{\Theta}$. Then, we computed the likelihood of $\hat{\Theta}$ given the test set, i.e., the left-out localization response, r_i. We repeated this procedure N times until every single non-outlier localization response was treated as a test set. This resulted in a distribution of $p(r_i | \hat{\Theta}, M)$ for each model. Finally, to compare model performance, we

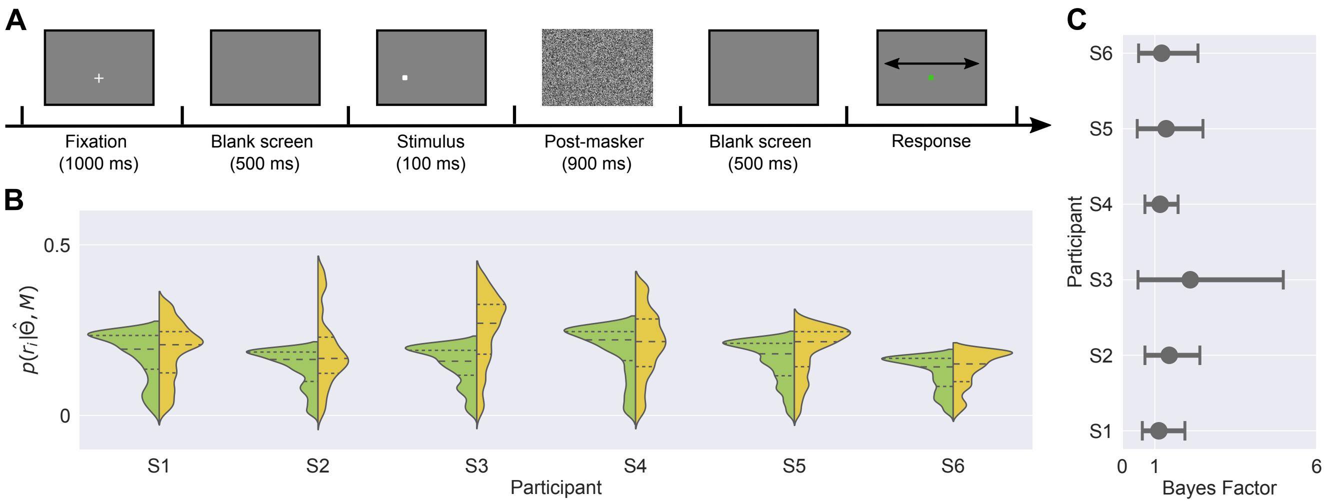

Figure S1. Procedure and results of the pointing practice task. (**A**) Timeline. Participants were presented with a white target square (8 x 8 pixels) at one of the stimulus locations: ±2.5°, ±7.5°, ±12.5° or ±17.5°. The target stimulus was followed by a post-masker and a brief blank screen. Participants adjusted the pointing device to localize the target. Visual feedback for the location of the pointing device was constantly provided, as shown by the green square. (**B**) The kernel density estimate of the distribution of the likelihoods of best-fit parameters for each model (green: **M0**, yellow: **M1**) given left-out localization responses r_i. Dashed lines: first quartile, median and the third quartile. (**C**) The 5th and 95th percentiles of the Bayes factors. Dots: mean.

calculated a Bayes factor for the likelihood given each test set, i.e., the ratio of $p(r_i|\hat{\Theta}, M)$ between **M1** and **M0** ($p(r_i|\hat{\Theta}_{M_1}, M_1)/p(r_i|\hat{\Theta}_{M_0}, M_0) \gg 1$ provides evidence for **M1**, and $0 < p(r_i|\hat{\Theta}_{M_1}, M_1)/p(r_i|\hat{\Theta}_{M_0}, M_0) \ll 1$ provides evidence for **M0**).

Results. Figure S1B illustrates the kernel density estimates, plotted using the Seaborn python library [1], of the distributions of the likelihoods of best-fit model and parameters given the left-out localization responses, $p(r_i|\hat{\Theta}, M)$, for **M0** and **M1**. We observe substantial overlap between these two distributions for all six participants, suggesting that **M1** does not outperform **M0** significantly. **Figure S1C** shows that the 5th percentile of the Bayes factor does not exceed 1 for any participant, further confirming that **M1** does not outperform **M0**. Therefore, we calculated the setting noise plus participants' memory/setting noise σ_r based on **M0** for each participant (M = $1.85°$, range = $1.55 - 2.29°$) and used these values in the main model-fitting section.

S3. Model Predictions by the Causal-inference Model with a Supra-Modal Learning Rate and Modality-Specific Learning Rates

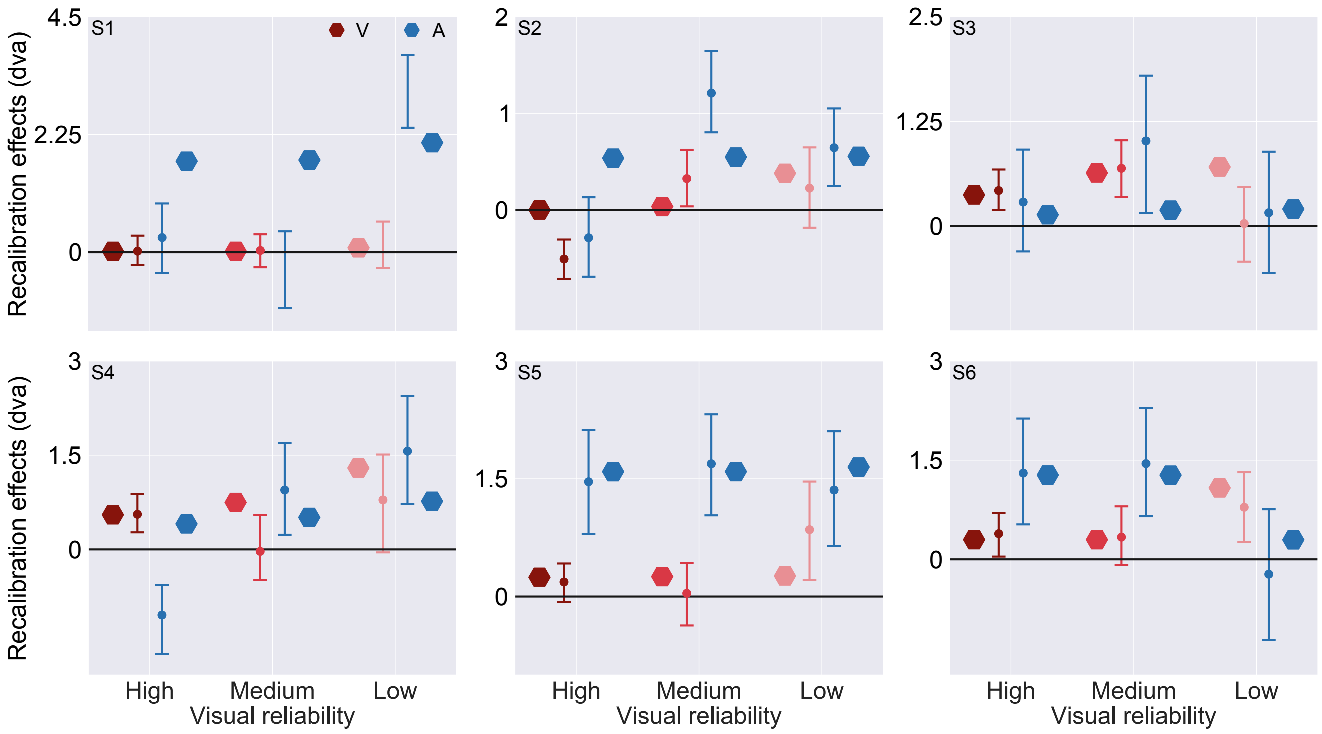

Figure S2. Model predictions (hexagons) by the causal-inference model that assumes model averaging and a supra-modal learning rate for the visual (red) and auditory (blue) recalibration effects as a function of visual reliability.

	a	b	$\sigma'_{AV,A}$	σ'_{AV,V_1}	σ'_{AV,V_2}	σ'_{AV,V_3}	α	$p_{C=1}$	λ_{AV}	nLL
S1	1.82	-1.45	1.20	0.12	0.12	0.24	0.0794	0.71	0.06	3204.3
S2	1.03	-0.21	1.06	0.01	0.27	0.88	0.0020	1.00	0.06	2957.0
S3	1.91	-1.30	0.89	1.48	1.63	1.66	0.0088	0.90	0.06	3093.5
S4	1.20	-6.44	0.81	0.94	0.98	1.05	0.0948	0.12	0.06	3187.6
S5	1.85	-6.10	1.36	0.53	0.54	0.54	0.0330	0.99	0.06	3234.6
S6	2.75	-0.61	10.39	5.00	5.00	20.00	0.0062	0.09	0.06	3556.9

Table S1. Best-fit parameters and the overall negative log likelihood of the causal-inference model that assumes model averaging and a supra-modal learning rate.

	a	b	$\sigma'_{AV,A}$	σ'_{AV,V_1}	σ'_{AV,V_2}	σ'_{AV,V_3}	α_A	α_V	$p_{C=1}$	λ_{AV}	nLL
S1	1.83	-1.51	0.88	0.01	0.76	1.70	0.0987	1.25E-08	0.74	0.06	3198.7
S2	1.03	-0.21	0.44	0.06	1.21	2.60	0.0496	5.17E-04	0.98	0.06	2946.0
S3	1.90	-1.31	0.79	1.82	2.05	2.05	0.0153	0.0057	0.78	0.06	3093.7
S4	1.22	-6.39	0.52	0.95	0.95	0.98	0.0761	0.0275	0.90	0.06	3187.4
S5	1.85	-6.10	1.36	0.53	0.54	0.54	0.0325	0.0359	0.99	0.06	3234.5
S6	2.75	-0.59	4.93	4.09	4.09	20.00	0.0015	5.78E-04	1.00	0.06	3556.5

Table S2. Best-fit parameters and the overall negative log likelihood of the causal-inference model that assumes model averaging and modality-specific learning rates.

	a	b	$\sigma'_{AV,A}$	σ'_{AV,V_1}	σ'_{AV,V_2}	σ'_{AV,V_3}	α_A	α_V	$p_{C=1}$	λ_{AV}	nLL
S1	1.83	-1.49	0.69	0.18	0.78	1.53	0.0979	5.11E-08	0.97	0.06	3199.1
S2	1.03	-0.21	0.48	0.07	1.21	2.57	0.0416	6.62E-04	0.97	0.06	2946.0
S3	1.91	-1.29	0.69	1.52	1.69	1.69	0.0098	0.0051	0.98	0.06	3093.6
S4	1.21	-6.37	0.95	0.76	0.79	0.86	0.0312	0.0383	0.63	0.06	3185.8
S5	1.85	-5.99	1.52	1.69	1.69	6.53	0.0642	0.0039	0.28	0.06	3234.0
S6	2.75	-0.56	5.07	5	5	19.78	0.0378	0.0100	0.034	0.06	3556.0

Table S3. Best-fit parameters and the overall negative log likelihood of the causal-inference model that assumes probability matching and modality-specific learning rates.

S4. Model Predictions by the Reliability-Based Model

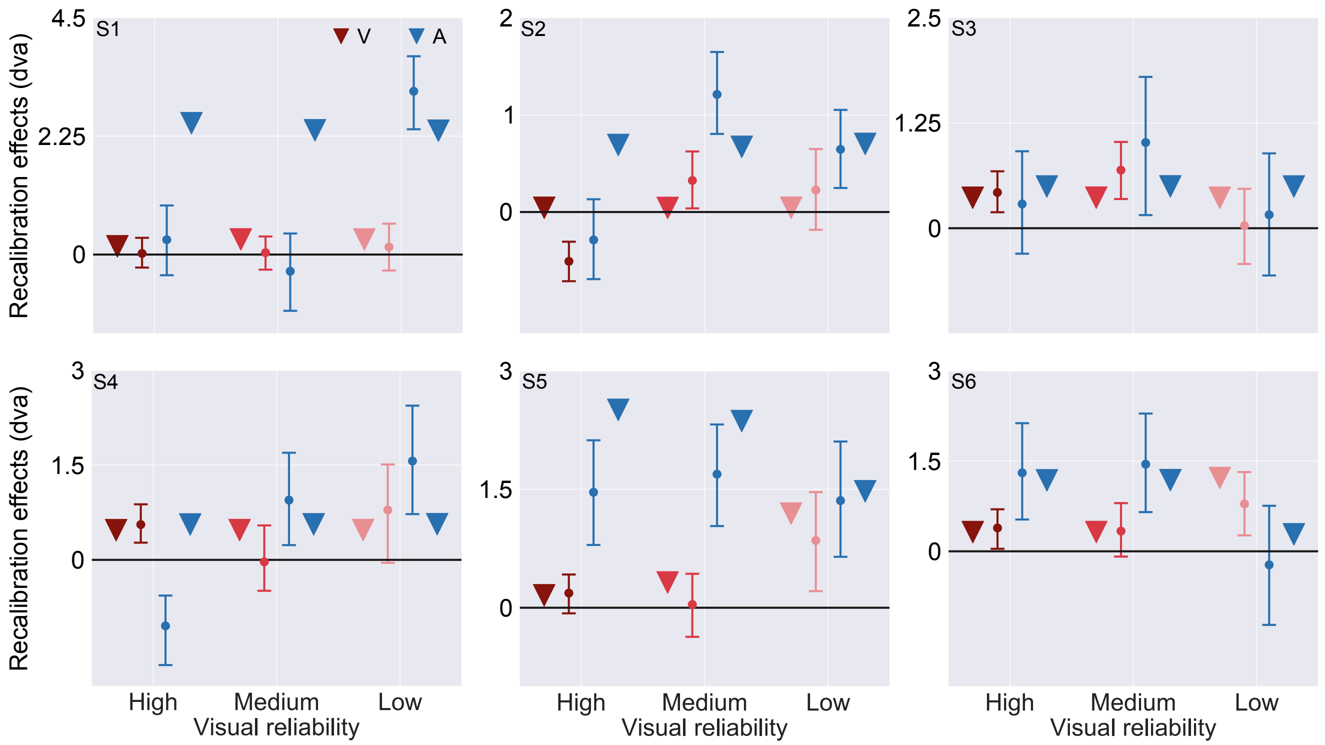

Figure S3. Model predictions (triangles) by the reliability-based model for the visual (red) and auditory (blue) recalibration effects as a function of visual reliability.

	a	b	$\sigma_{AV,A}$	σ_{AV,V_1}	σ_{AV,V_2}	σ_{AV,V_3}	α	λ_{AV}	nLL
S1	1.82	-1.91	20	5	6.97	6.97	0.0030	0.06	3230.8
S2	1.03	-0.34	20	5	5	5	0.0013	0.06	2955.6
S3	1.91	-1.33	5.86	5	5	5	7.3184E-04	0.06	3101.3
S4	1.21	-6.39	5.46	5	5	5	0.0014	0.06	3195.4
S5	1.86	-5.90	20	5	7.35	18	0.0027	0.06	3248.8
S6	2.75	-0.58	9.63	5	5	20	9.4972E-04	0.06	3557.1

Table S4. Best-fit parameters and the overall negative log likelihood of the reliability-based model.

S5. Model Predictions by the Fixed-Ratio Model

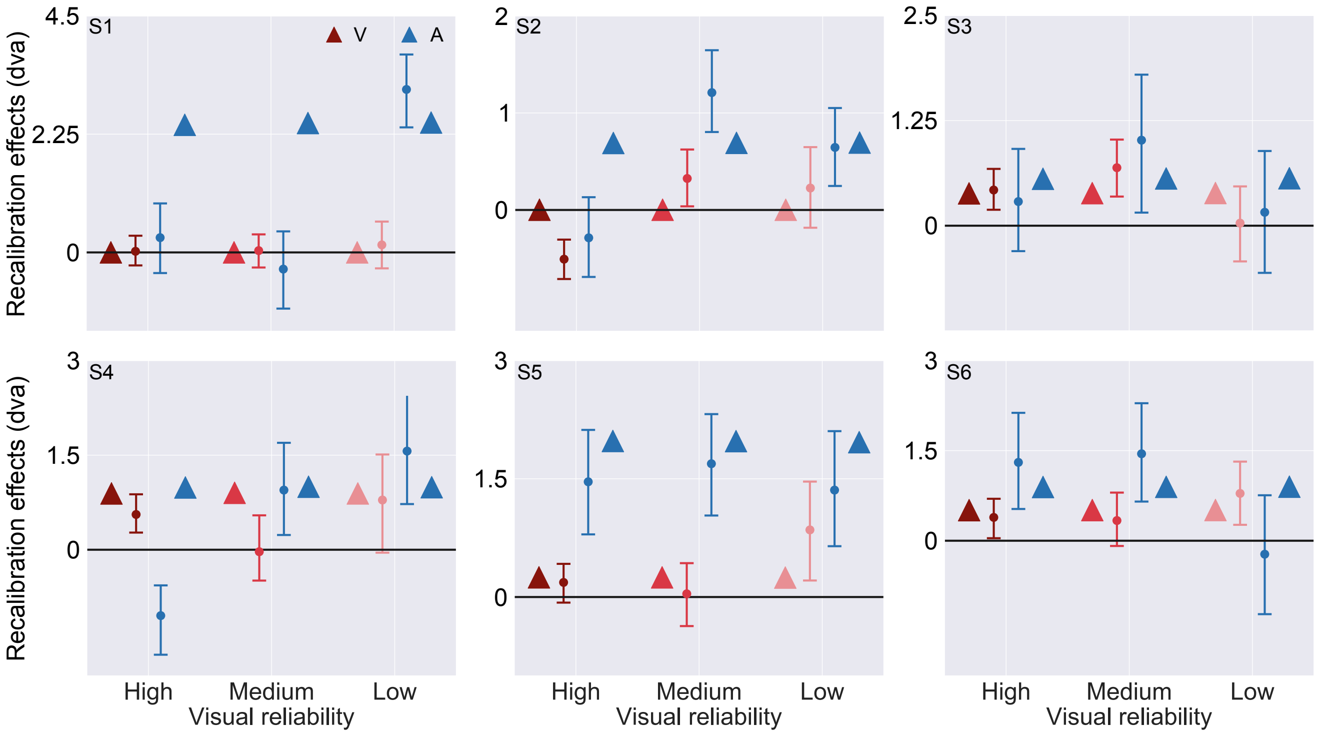

Figure S4. Model predictions (triangles) by the fixed-ratio model for the visual (red) and auditory (blue) recalibration gains as a function of visual reliability.

	a	b	$\sigma_{AV,A}$	σ_{AV,V_1}	σ_{AV,V_2}	σ_{AV,V_3}	α_A	α_V	λ_{AV}	nLL
S1	1.82	-1.82	20	4.81	12	20	0.0028	1.1936E-06	0.06	3224.1
S2	1.03	-0.33	20	5	12	17.94	0.0012	3.8607E-06	0.06	2955.4
S3	1.90	-1.31	20	4.70	10.44	19.98	4.8395E-04	3.3287E-04	0.06	3100.9
S4	1.21	-6.33	20	4.98	10.19	20	0.0014	0.0013	0.06	3190.2
S5	1.86	-5.79	20	5	5.64	8.59	0.0019	2.4515E-04	0.06	3250.1
S6	2.75	-0.56	20	3.09	11.73	19.98	5.6525E-04	3.2094E-04	0.06	3559.1

Table S5. Best-fit parameters and the overall negative log likelihood of the fixed-ratio model.

S6. Model Predictions on the Biases in Auditory Spatial Perception Relative to Visual Spatial Perception

In this section, we examined whether the reliability-based, the fixed-ratio, and the causal-inference models of cross-modal recalibration can capture participants' auditory perceptual biases relative to visual spatial perception. We did so by comparing the proportional (a_A) and constant (b_A) shifts of visual relative to auditory location estimated from those three models with those estimated from the bimodal spatial-discrimination task, which correspond to the slope and the intercept of the linear regression of PSEs on auditory locations (**Fig. 4A**), and with the proportional and constant biases estimated from the pre-recalibration phase, which correspond to the slope and the intercept of the linear regression on auditory localizations (**Fig. 6A**). As expected, model estimates of the proportional and constant biases are very similar across the three models because they predict different behaviors only in the post-recalibration phase (**Fig. S5**). More importantly, the model estimates are close to those estimated from the two tasks, indicating that all three models can capture participants' auditory perceptual biases relative to visual spatial perception (**Fig. S5**).

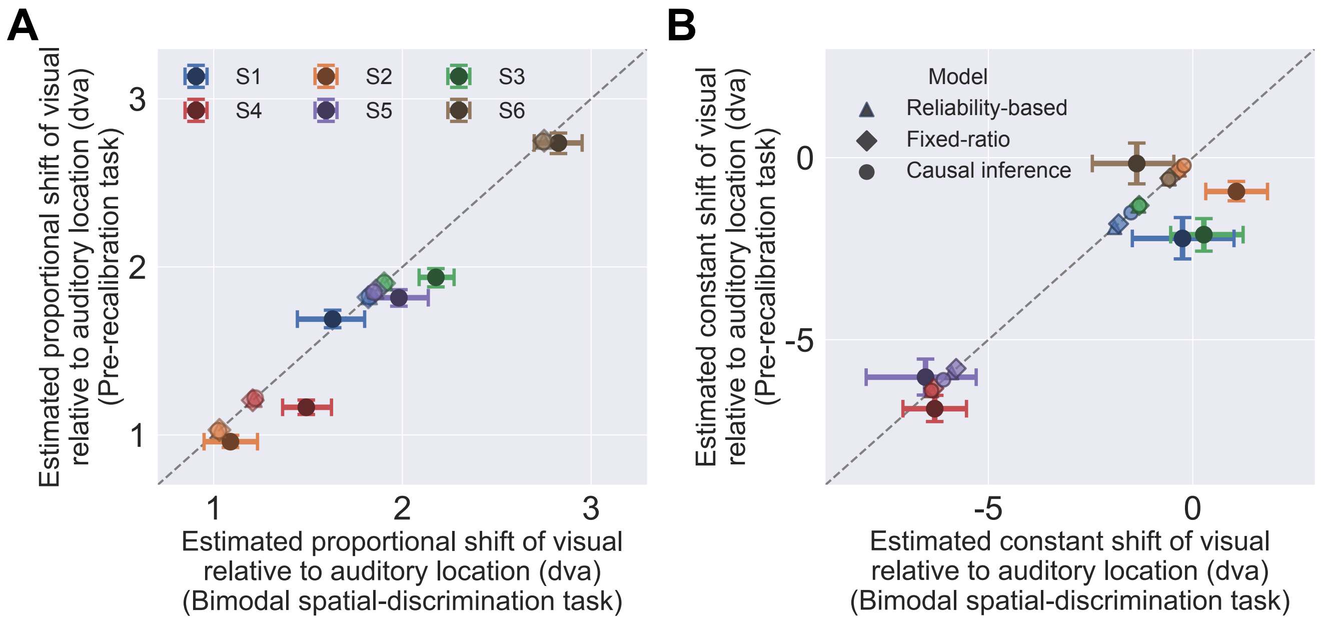

Figure S5. The proportional shift (**A**) and the constant shift (**B**) estimated from the linear regression of the auditory localization responses pooled from the pre-recalibration phase (vertical axis) and of PSEs from the bimodal spatial-discrimination task (horizontal axis) on four auditory locations for all participants. Error bars: 95% bootstrapped confidence intervals. Triangles, diamonds, circles: model predictions by the reliability-based, the fixed-ratio, and the causal-inference model, respectively.

S7. Biases in Visual Spatial Localization

The goal of this control experiment was to measure the biases in visual spatial localization and examine whether the biases were caused by a fixed bias in sensory measurements or a prior distribution over visual location.

Participants. Four participants (3 females, aged 21-29, all right-handed, all of them were naive to the purposes of this control experiment) participated exclusively in this experiment. Two were recruited from outside of New York University. None reported any visual, auditory or motor impairment. Experimental protocols were approved by the Institutional Review Board at New York University. All participants gave informed consent. One volunteered for participation and the other three were compensated $10 per hour.

Procedure. The procedure was very similar to the pre-recalibration task. In each trial, participants were presented with either a visual or an auditory stimulus at one of the six target locations (±2.5, ±7.5° or ±12.5°) for 100 ms. The visual stimulus was followed a 900 ms visual post-masker. After stimulus presentation, participants were asked to use the pointing device to localize the stimulus and use the foot pedal to register their response. Unlike the pre-recalibration task, in this control experiment, participants were not provided with visual feedback for the location of the pointing device during adjustment. Each target location was repeated twelve times for each modality within a session, resulting in a total of 144 trials. The reliability of the visual stimulus also had three levels (high reliability: $\sigma_x = 2.2^{\circ}$, medium reliability: $\sigma_x = 5.4^{\circ}$, low reliability: $\sigma_x = 8.7^{\circ}$), and each of the three levels was tested in two separate sessions.

Analysis. The visual localization responses were first filtered before being analyzed. For each level of visual reliability, we first pooled the data from the two sessions with the same visual reliability. Then, we calculated the demeaned localization responses for each visual stimulus location and computed the standard deviation of all the demeaned localization responses across locations, assuming that the standard deviation is the same across locations but different across reliability conditions. Finally, we computed a *z*-score for each localization response and identified outliers (*z*-score < -3 or *z* > 3). After excluding the outliers,

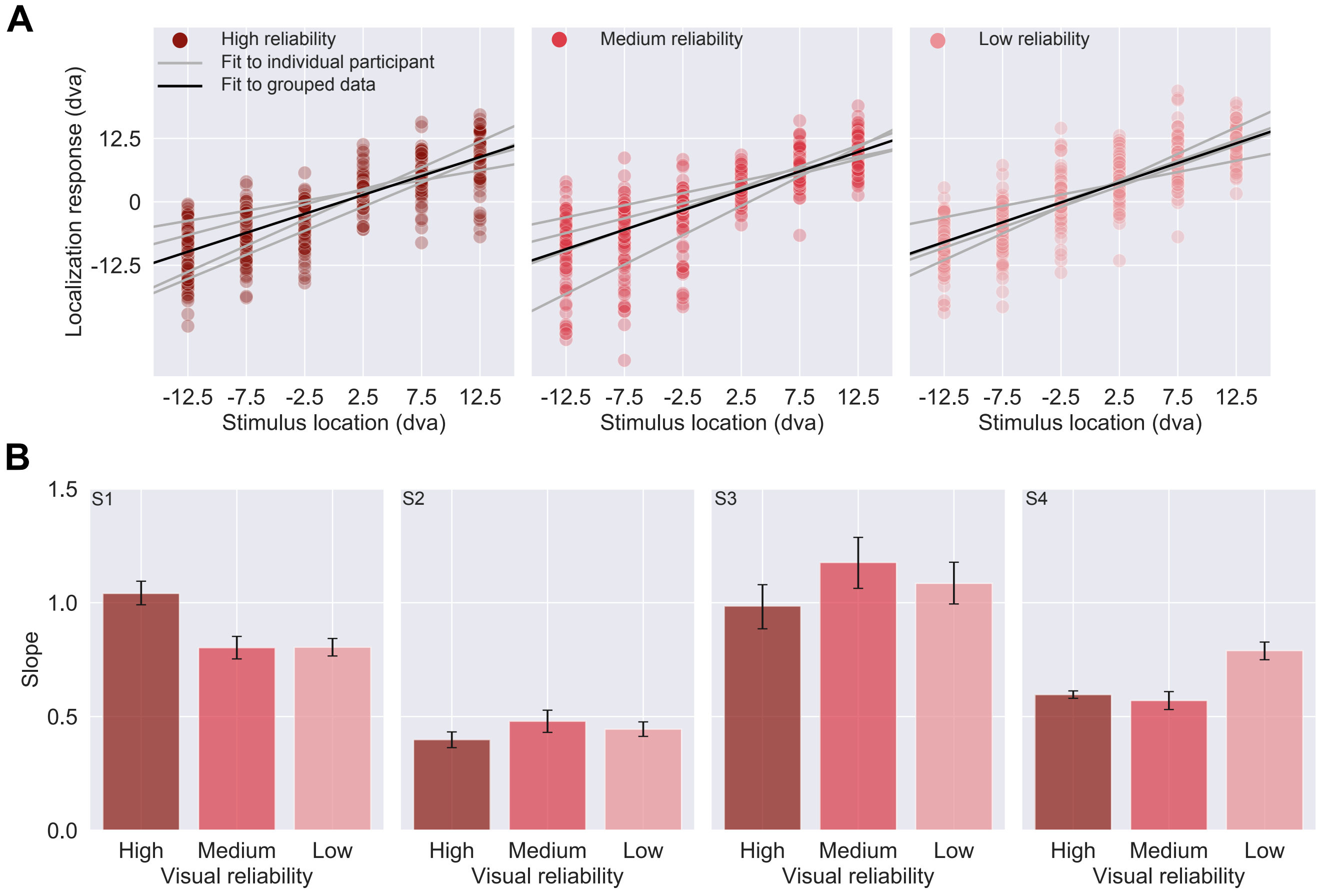

Figure S6. Results of the unimodal visual spatial-estimation tasks. (**A**) Localization responses for the high (left panel, dark pink), medium (middle panel, pink), and low visual reliability condition (right panel, light pink). Dots: individual localization responses; grey solid lines: linear regression fit to the localization responses of each participant; black solid lines: linear regression fit to the pooled localization responses across all participants. (**B**) The slope of the linear regression fits. Error bars: 95% bootstrapped confidence intervals.

we fitted a linear regression (**Fig. S6A**). To obtain error bars on the slope of the linear regression, we resampled with replacement, creating 1,000 bootstrapped datasets (the samples for each location matched the number of non-outliers responses), re-fitted a linear regression to each resampled dataset, and took the 2.5th and 97.5th percentiles of the 1,000 slopes as the 95% confidence interval. This procedure was repeated for all three visual-reliability conditions (**Fig. S6B**).

Results. We observed central biases in visual spatial perception in three out of four participants, which is in line with previous studies [2-4]. If the central biases were caused by a central prior, then we would expect to see decreasing slopes with decreasing visual reliability. In other words, as the visual stimulus becomes less reliable, an ideal Bayesian observer would give more weight to the central prior. Consequently, the final visual location estimates

would be more biased towards the central fixation, which in turn makes the slope of the linear regression shallower. However, we do not observe such a pattern in the data. For most participants, the slope does not decrease monotonically as visual reliability decreases, which left us with the alternative explanation: the biases observed in the localization responses were more likely to be dominated by a fixed bias in sensory measurements. If that's the case, then the final visual location estimate at a given location would be biased towards the central fixation by the same amount at all levels of visual reliability, and hence the slope of the linear regression would not become shallower as visual reliability decreases. In general, our results are better captured by this explanation than by the effects of a central visual prior.

S8. Testing the Biases of the Log Likelihood of Models and Parameters as a Result of Fitting a Univariate/Bivariate Gaussian or Applying a Kernel Smoothing Function to the Visual and Auditory Shifts

Fitting models to the localization responses from the post-recalibration task requires an estimate of the distribution of the visual and auditory measurement-shifts. Unfortunately, it is impossible to derive a closed-form expression for the joint probability of the visual and auditory measurement-shifts, $P(\Delta_{A,i,j}, \Delta_{V_i,j} | M, \Theta_3^3)$, because $\Delta_{A,i,j}$ and $\Delta_{V_i,j}$ are stochastic. Therefore, the only sensible option we had was to approximate $P(\Delta_{A,i,j}, \Delta_{V_i,j} | M, \Theta_3^3)$ while minimizing the bias in the resulting model-likelihood estimates. For the reliability-based and the fixed-ratio models, since $\Delta_{A,i,j}$ is proportional to $\Delta_{V_i,j}$, we approximated $P(\Delta_{A,i,j}, \Delta_{V_i,j} | M, \Theta_3^3)$ by fitting a univariate Gaussian distribution to $\Delta_{V_i,j}$; for the causal-inference model a bivariate Gaussian was needed. We checked whether the Gaussian provided an adequate fit to the 1,000 sample (Δ_A, Δ_V) pairs by computing the correlation between the proportion of pairs in each bin of a histogram of the samples with the predicted probability from the fit Gaussian, accepting the Gaussian fit if the value of R^2 is greater than 0.925. For the fixed-ratio and reliability-based models, this criterion was always met, so the Gaussian model was used. For the causal-inference model, in cases where this criterion was not met (M = 39.4% of cases, SD = 20.8% across participants), we used a kernel density estimate [5, 6]. In the following two subsections, we show that the univariate Gaussian is a close approximation to $P(\Delta_{V_i,j} | M, \Theta_3^3)$ for the reliability-based and the fixed-ratio models, and the bivariate Gaussian is a close approximation to $P(\Delta_{A,i,j}, \Delta_{V_i,j} | M, \Theta_3^3)$ in most cases for the causal-inference model. We also ran simulations to show that approximating the distribution in this way leads to a nearly unbiased estimate of the log likelihood of the model and parameters given the data.

S8.1. The Reliability-Based and the Fixed-Ratio Models

We first confirmed that, in most (if not all) cases, simulated auditory and visual shifts by these two models can be fit extremely well by a univariate Gaussian. For each participant, we

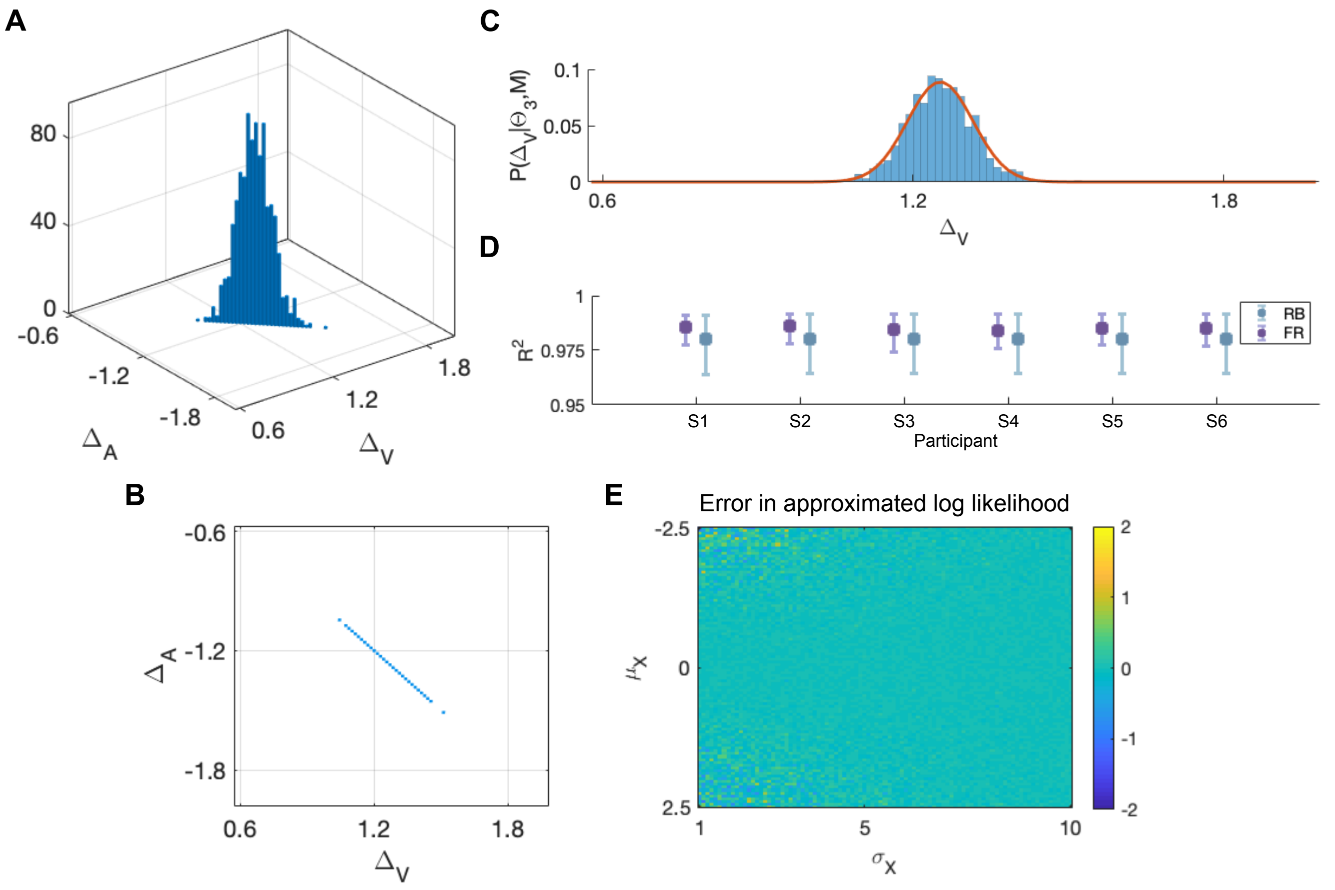

Figure S7. Examination of the biases caused by approximating $P(\Delta_{V_i,j}|M,\Theta_3^3)$ by fitting a univariate Gaussian. (**A**) The bivariate histogram of 1,000 pairs of simulated visual and auditory shifts in a visual-left-of-auditory session given the FR model for a random combination of model parameters. (**B**) A top view of the bivariate histogram. Horizontal axis: visual shift; vertical axis: auditory shift. (**C**) The univariate histogram of 1,000 visual shifts. Red solid line: the best-fit Gaussian distribution. R^2 was used to indicate how strong the correlation was between the observed data (histogram) and the predicted probabilities (red solid line). (**D**) R^2 calculated from over 10,000 iterations given the reliability-based (purple) and the fixed-ratio (blue) models for all six subjects (horizontal axis). (**E**) The difference between the recovered and the true log likelihood as a function of the standard deviation σ_X (horizontal axis) and the mean μ_X (vertical axis).

ran the BADS model-fitting software [7] 10 times; each run had a different starting point. This resulted in over 10,000 iterations ($\leq$ 10,000 tested combinations of parameters). For each iteration, we simulated 1,000 pairs of $\Delta_{A,i,j}$ and $\Delta_{V_i,j}$ in the ith visual-reliability condition and j^{th} recalibration-direction condition. **Figure S7A** shows the histogram of 1,000 pairs of $\Delta_{A,i,j}$ and $\Delta_{V_i,j}$ in one example session given the fixed-ratio model for a random combination of parameters Θ_3^3. The upper and lower bounds of the histogram are chosen so that the binning range is three times as large as the range of the data for both axes, i.e., $lb = \Delta_{min} - (\Delta_{max} - \Delta_{min})$ and $ub = \Delta_{max} + (\Delta_{max} - \Delta_{min})$. Note that data only exist along the diagonal (**Fig. S7B**), which allowed us to fit a univariate distribution to the $\Delta_{V_i,j}$ samples.

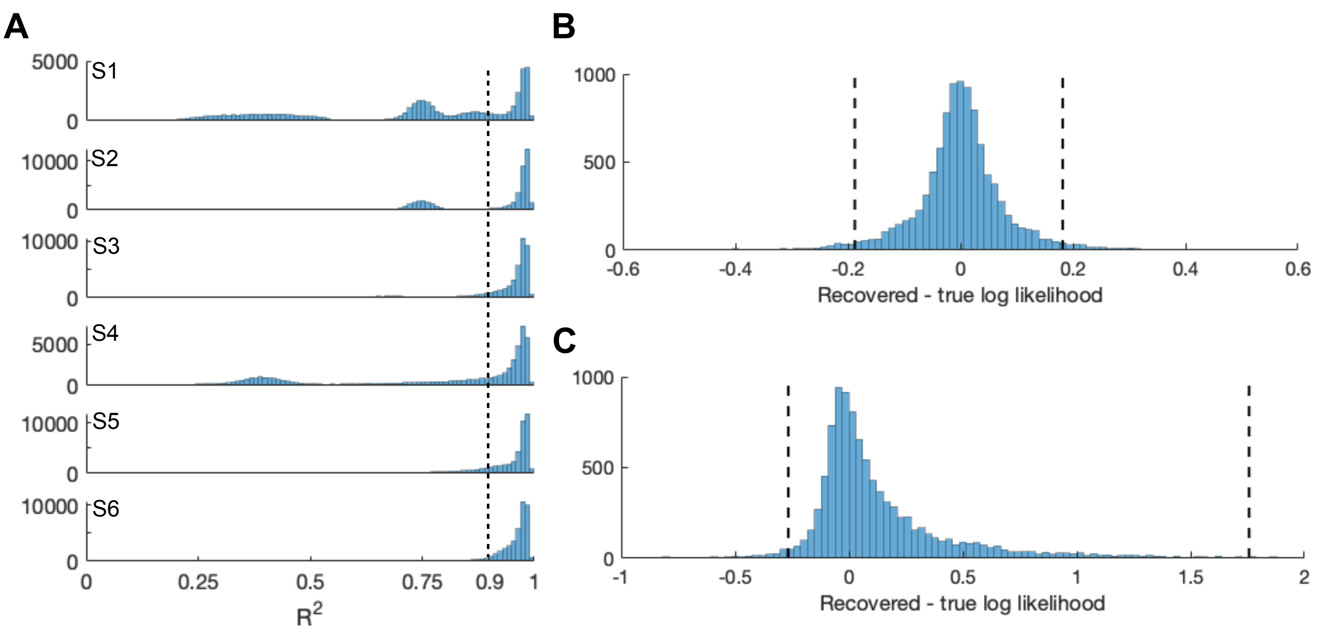

Figure S8. Examination of the biases caused by approximating $P(\Delta_{A,i,j}, \Delta_{V_i,j} | M, \Theta_3^3)$ by fitting a bivariate Gaussian distribution or applying a kernel smoothing function. (**A**) R^2 calculated from over 40,000 iterations given the causal-inference model for all six subjects. Dashed line: the R^2-threshold we chose for determining when to fit a bivariate Gaussian. (**B**) The histogram of the difference between the log likelihood recovered by fitting a bivariate Gaussian and the true value. Dashed lines: 2.5% and 97.5% percentiles. (**C**) The histogram of the difference between the log likelihood recovered by applying a kernel smoothing function and the true value. Dashed lines: 2.5% and 97.5% percentiles.

The 1,000 samples were then discretized into 100 bins. **Figure S7C** shows the data projected onto the ΔV axis as well as the best-fit univariate Gaussian distribution. We used R^2 as an indicator of how well a Gaussian distribution fits the data. R^2 was calculated by squaring the correlation coefficient between the proportion of the simulated data in each bin and the predicted probabilities for those bins from the best-fit Gaussian distribution. **Figure S7D** shows the 2.5th and 97.5th percentiles of R^2 for all six participants given the reliability-based and the fixed-ratio models. R^2 is strictly greater than 0.925 for all participants and for both models. Therefore, we conclude that a univariate Gaussian is a close approximation to $P(\Delta_{V_i,j} | M, \Theta_3^3)$.

Next, we assumed that the true shape of $P(\Delta_{V_i,j} | M, \Theta_3^3)$ was indeed a univariate Gaussian, and tested whether fitting a Gaussian to the $\Delta_{V_i,j}$ sampled from $P(\Delta_{V_i,j} | M, \Theta_3^3)$ would lead to biased estimates of the log likelihood of the parameters given the data. Simulations were run without assuming any specific model, so we drop the notation M as well as i and j that index each condition, so that the probability distribution of the shifts

$P(\Delta_{V_{i,j}}|M,\Theta_3^3)$ becomes $P(\Delta_V|\mu_{\Delta_V},\sigma_{\Delta_V})$ and the likelihood of the data $P(X|M,\Theta_3^3)$ is replaced by $P(X|\mu_{\Delta_V},\sigma_{\Delta_V})$. Without loss of generality, we fixed the mean and the standard deviation of the probability of visual shifts ($\mu_{\Delta_V}=0°$ and $\sigma_{\Delta_V}=0.5°$) and simulated trials for a stimulus at the center ($s_V=0°$). We determine how much the likelihood of the data estimated using our sampling procedure differs from the likelihood computed using the assumed Gaussian distribution of shifts $P(\Delta_V|\mu_{\Delta_V},\sigma_{\Delta_V})$. For any "true" value of shift Δ_V and motor/response noise σ_X, we generated 96 localization responses x_n, analogous to our experimental procedure, by sampling from a Gaussian distribution $x_n \sim \mathcal{N}(\mu_X,\sigma_X)$, where $\mu_X = s_V + \Delta_V$. We calculated the likelihoods for a grid of "true" shift values Δ_V (from $-2.5°$ to $2.5°$ in steps of $0.05°$) and response noise values σ_X (from 1 to $10°$ in steps of $0.1°$). We calculated the true log likelihood $\log P(X|\mu_{\Delta_V},\sigma_{\Delta_V})$ by numerical integration:

$$\log P(X|\mu_{\Delta_V},\sigma_{\Delta_V}) = \log\left(\int P(X|\Delta_V,\mu_{\Delta_V},\sigma_{\Delta_V})P(\Delta_V|\mu_{\Delta_V},\sigma_{\Delta_V})d\Delta_V\right)$$

$$\approx \log\left(\frac{ub_V - lb_V}{100}\sum_{k_V=1}^{100}\left[\prod_{n=1}^{96} p(x_n|\Delta_V(k_V),\mu_{\Delta_V},\sigma_{\Delta_V})\right]p(\Delta_V(k_V)|\mu_{\Delta_V},\sigma_{\Delta_V})\right).$$

Note that Δ_A is omitted in $p(x_n|\Delta_V(k_V),\mu_{\Delta_V},\sigma_{\Delta_V})$ because Δ_A is proportional to Δ_V (see **Supplement: S14**).

To approximate $P(\Delta_V|\mu_{\Delta_V},\sigma_{\Delta_V})$, we randomly generated 1,000 samples of Δ_V drawn from $P(\Delta_V|\mu\Delta_V,\sigma_{\Delta_V})$, analogous to the process of model fitting, and then fitted a univariate Gaussian to those samples, denoted as $\tilde{P}(\Delta_V|\mu_{\Delta_V},\sigma_{\Delta_V})$. The recovered log likelihood was calculated using Eq. S1 with $P(\Delta_V|\mu_{\Delta_V},\sigma_{\Delta_V})$ replaced by $\tilde{P}(\Delta_V|\mu_{\Delta_V},\sigma_{\Delta_V})$. **Figure S7E** shows the difference between the recovered log likelihood and the true one as a function of μ_X and σ_X. The difference is close to zero for all combinations of μ_X and σ_X. Therefore, we conclude that approximating the probability distribution of the visual and the auditory shifts by fitting a univariate Gaussian leads to almost unbiased estimates of log likelihood of the parameters given the data.

S8.2. The Causal-Inference Model

As in the previous subsection, for each participant we ran the BADS model-fitting software [7] 10 times (over 40,000 iterations), each of which had a different starting point. In each iteration, we fitted a bivariate Gaussian to the simulated 1,000 pairs of $\Delta_{A,i,j}$ and $\Delta_{V_i,j}$. We binned the 1000 pairs into a 2-d histogram of 100×100 bins. We calculated the value of R^2 for a correlation between the predicted probabilities of each bin and the proportion of simulated pairs in that bin. We investigated, of all iterations, in how many of them the auditory and visual shifts can be well captured by a bivariate Gaussian. **Figure S8A** shows the histograms of R^2 for all six participants. The fact that R^2 is greater than 0.925 in more than half cases (M = 60.6%, SD = 20.8%) suggests that a bivariate Gaussian provides a decent approximation to $P(\Delta_{V_i,j}, \Delta_{A,i,j} \mid M, \Theta_3^3)$.

Next, we assumed that the true shape of $P(\Delta_{A,i,j}, \Delta_{V_i,j} \mid M, \Theta_3^3)$ is a bivariate Gaussian, and tested whether fitting a bivariate Gaussian would lead to biased estimates of the log likelihood of the parameters given the data. Again, simulations were run without assuming any model, so we drop the notation M as well as i and j. The parameters we varied were the mean visual shifts, the mean auditory shifts, and the covariance of the joint probability distribution, denoted as μ_{Δ_A}, μ_{Δ_V} and $\Sigma_{\Delta_A,\Delta_V}$. We tested 10,000 combinations of these parameters by randomly drawing μ_{Δ_A} from a uniform distribution $U(-4,4)$, μ_{Δ_V} from a uniform distribution $U(-1,1)$, and $\Sigma_{\Delta_A,\Delta_V}$ from an inverse Wishart distribution. Then, we generated 48 localization responses x_n for each modality from a bivariate Gaussian distribution $x_n \sim \mathcal{N}(\mu_X, \Sigma_X)$, where $\mu_X = [s_V + \Delta_V, s_A + \Delta_A]$ and Σ_X was fixed at $\begin{bmatrix} 2 & 0 \\ 0 & 5 \end{bmatrix}$. Again without loss of generality, we assumed that there was only one pair of auditory and visual stimulus locations, $s_V = -2.5°$ and $s_A = 2.5°$, and that participants did not recalibrate either vision or audition during the audiovisual recalibration task, i.e., $\Delta_V = \Delta_A = 0°$. As in the previous subsection, to recover the log likelihood, we randomly generated 1,000 samples and fitted a bivariate Gaussian to those samples. Then we calculated the true log likelihood by numerical integration:

$$\log P(X \mid \mu_{\Delta_A}, \mu_{\Delta_V}, \Sigma_{\Delta_A,\Delta_V}) = \log \left[\iint P(X \mid \Delta_A, \Delta_V, \mu_{\Delta_A}, \mu_{\Delta_V}, \Sigma_{\Delta_A,\Delta_V}) P(\Delta_A, \Delta_V \mid \mu_{\Delta_A}, \mu_{\Delta_V}, \Sigma_{\Delta_A,\Delta_V}) d\Delta_A d\Delta_V \right]$$

$$\approx \log \left[\frac{ub_V - lb_V}{nb} \frac{ub_A - lb_A}{nb} \sum_{k_V=1}^{nb} \sum_{k_A=1}^{nb} p(X \mid \Delta_A(k_A), \Delta_V(k_V), \mu_{\Delta_A}, \mu_{\Delta_V}, \Sigma_{\Delta_A,\Delta_V}) \right.$$

$$\left. p(\Delta_A(k_A), \Delta_V(k_V) \mid \mu_{\Delta_A}, \mu_{\Delta_V}, \Sigma_{\Delta_A,\Delta_V}) \right].$$

Finally, we compared how different the recovered values from sampling were from the true log likelihood.

Figure S8B shows the distribution of the difference between the true log likelihood and the one recovered by fitting a bivariate Gaussian to 1,000 pairs of Δ_A and Δ_V. The difference lies within a very small range. Therefore, we conclude that approximating auditory and visual shifts by fitting a bivariate Gaussian leads to nearly unbiased estimates of log likelihood of parameters given data. Besides fitting a bivariate Gaussian, we also tested how biased the estimate of log likelihood would be if we fit the auditory and visual shifts using a kernel density estimate using a Gaussian kernel [5, 6]. The results of this analysis are shown in **Figure S8C**. The difference also lies within a reasonably small range. However, we cannot draw the conclusion that the use of a kernel density estimate always returns an unbiased estimate of log likelihood, because we do not have knowledge of the true function for $P(\Delta_A, \Delta_V \mid \mu_{\Delta_A}, \mu_{\Delta_V}, \Sigma_{\Delta_A,\Delta_V})$, especially when a bivariate Gaussian is obviously not an appropriate fit.

Since a bivariate Gaussian sometimes fails to provide a good approximation to the joint probability of auditory and visual shifts, we had to set an arbitrary R^2 threshold to determine when to fit a bivariate Gaussian and when to use a kernel density estimate. We chose 0.925, which is relatively conservative, as the R^2 threshold.

S9. Trade-Offs Between Free Parameters

S9.1. Trade-Off Between the Constant Bias in Auditory Spatial Perception and the Center of the Prior Distribution over Stimulus Location

In this section, we show that there is a trade-off between the constant bias b_A and the mean of a Gaussian-shape spatial prior μ'_P. Based on Eq. 17, $\mu_{\hat{s}'_A} = c_A s'_A + f_A = c_A(a_A s_A + b_A) + f_A$. By moving terms around, we have $b_A = (\mu_{\hat{s}'_A} - f_A)/c_A - a_A s_A = (\mu_{\hat{s}'_A} - \mu'_P \sigma_P^{'-2}/(\sigma_A^{'-2} + \sigma_P^{'-2}))/c_A - a_A s_A = -k_1 \mu'_P + k_0$. Thus, it is obvious that b_A and μ'_P trade off with each other; when one is increased, decreasing the other can generate the same value of $\mu_{\hat{s}'_A}$.

S9.2. Trade-Off Between the Common-Cause Prior and the Modality-Specific Learning Rate for Audition

In the causal-inference model of recalibration, we assumed that sensory systems undergo recalibration by shifting the measurements in the direction of the final location estimates $\hat{s}$, which combine the integrated location estimate $\hat{s}_{C=1}$ and the segregated location estimate $\hat{s}_{C=2}$, each weighted by the posterior probability of the corresponding causal structure $P(C=1 \,|\, m'_A, m'_V)$ and $P(C=2 \,|\, m'_A, m'_V)$. The posterior probability of a common cause $P(C=1 \,|\, m'_A, m'_V)$ is proportional to *a priori* knowledge about how often an auditory and a visual stimulus come from a common source, $p_{C=1}$. Therefore, the amount of recalibration is positively correlated with the common-cause prior. In other words, a high $p_{C=1}$ leads to a final location estimate $\hat{s}$ close to $\hat{s}_{C=1}$, and hence a greater amount of recalibration. In addition to $p_{C=1}$, the amount of recalibration is also positively correlated with a faster learning rate α [8]. Therefore, we expected that there would be a trade-off between these two free parameters to a certain degree.

To show quantitatively that those two free parameters trade off, we fixed the learning rate for recalibrating auditory cues α_A at one of four levels (0.05, 0.075, 0.1 and 0.125) and fitted the causal-inference model of recalibration to data X_3, which includes the responses

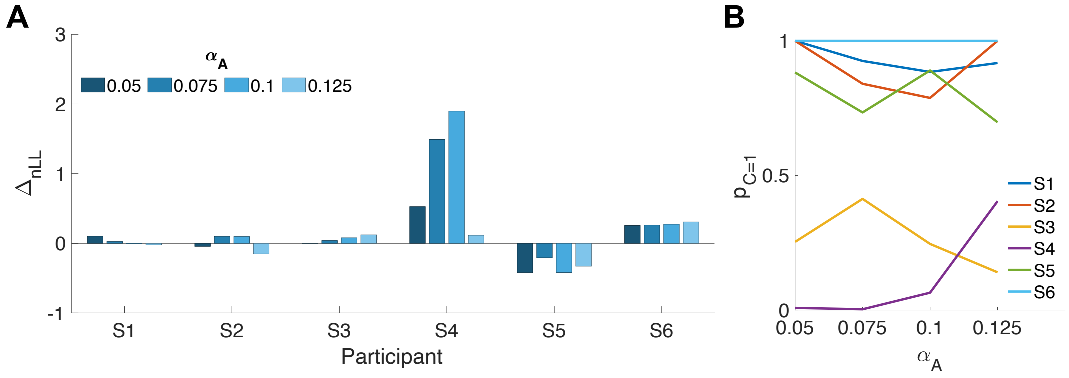

Figure S9. The trade-off between the learning rate and the *a priori* knowledge about two cues coming from a common source. (**A**) The difference between the negative log likelihood of the causal-inference model with the learning rate being fixed to either 0.05, 0.075, 0.1, or 0.125, and the negative log likelihood with the learning rate being a free parameter. (**B**) The best-fit prior probability of two cues coming from a common source $p_{C=1}$ as a function of the learning rate α_A.

from the bimodal spatial-discrimination task and the localization responses from the pre- and post-recalibration tasks.

We first checked whether the negative log likelihood of model $M_{\text{CI},\overline{\alpha}_A}$ (with fixed α_A) was significantly greater than the negative log likelihood of model M_{CI} (with α_A being a free parameter). Since $M_{\text{CI},\overline{\alpha}_A}$ is a nested model of M_{CI}, $-\log P(X_3 \,|\, M_{\text{CI},\overline{\alpha}_A}, \Theta_{3,\overline{\alpha}_A})$ has to be strictly greater than $-\log P(X_3 \,|\, M_{\text{CI}}, \Theta_3)$. A significant difference indicates that $p_{C=1}$ and α_A are both essential to capture the observed data. The results showed that the difference of the negative log likelihood between these two models is negligible for all participants (**Fig. S9A**), which supports our conjecture that α_A and $p_{C=1}$ trade off with each other. Note that there are some negative values because the “best-fit" parameters BADS return can sometimes correspond to local minima of the negative log likelihood. However, the model estimate $p_{C=1}$ does not decrease monotonically with increasing α_A (**Fig. S9B**), suggesting that there exists additional trade-offs with other parameters.

Indeed, the amount of recalibration depends not only on the learning rate and the prior probability of a common source, but also on cue reliability (see **Discussion**). Given that there is a non-monotonic relationship between cue reliability and the likelihood of the two cues coming from a common source, it is difficult to prove quantitatively that cue reliability trades off with the learning rate or the *a priori* knowledge about two cues coming from a common cause.

S10. Excluding Outlier Participants

We conducted a bimodal spatial-discrimination task with the aim of finding the visual locations that were perceived as co-located with four auditory locations (±2.5 and ±7.5°). Those four visual locations were then used in the subsequent recalibration sessions. The proportional and constant bias of visual relative to auditory spatial perception can be estimated by fitting a linear regression to the PSEs measured in the bimodal spatial-discrimination task or to the auditory localization responses pooled across all six pre-recalibration tasks. Both ways of estimating the proportional and constant bias should return similar results if the relative bias remained consistent throughout the entire study. In this section, we identify outlier participants by comparing the slope, which reflects the proportional bias, and the intercept, which reflects the constant bias, between those two regression lines. Participants who showed significantly different slope or intercept calculated one way compared to the other were identified as outliers and excluded from further analysis and model fitting.

Figure S10 shows the comparison between those two linear regressions for all seven participants. In the first subplot, data points represent the estimated proportional bias (the slope of the linear regression) of auditory localization responses pooled from all six pre-recalibration tasks (M = 1.612°, SD = 0.591) versus that of PSE's from the bimodal-spatial discrimination task (M = 1.795°, SD = 0.541). The mean absolute value of the difference between the two slopes is small (M = 0.200°, SD = 0.115). In the second subplot, data points represent the estimated constant bias (the intercept of the linear regression) from the baseline phase (M = -2.809°, SD = 2.409) versus from the bimodal spatial-discrimination task (M = -2.800°, SD = 3.223). The mean absolute value of the difference between the two intercepts is not close to zero due to the existence of outlier(s) (M = 1.971°, SD = 1.459). We excluded participants who showed a difference greater than 3 SDs or smaller than -3 SDs. One participant (marked in red) meets this criterion. With this outlier excluded, the mean absolute value of difference between slopes is reduced (M = 0.168°, SD = 0.091) and between intercepts as well (M = 1.442°, SD = 0.732).

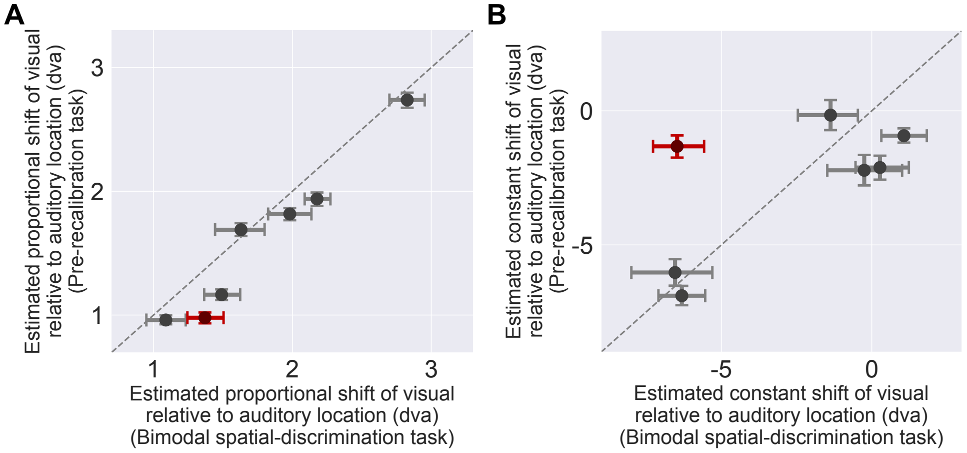

Figure S10. The proportional shift (**A**) and the constant shift (**B**) estimated from the linear regression of the auditory localization responses pooled from the pre-recalibration tasks (vertical axis) and of PSEs from the bimodal spatial-discrimination task (horizontal axis) on four auditory locations for all participants. The red dot corresponds to the data of the identified outlier. Error bars: 95% bootstrapped confidence intervals. Dashed line: identity line.

S11. Testing the Effectiveness of Playing the Masking Sound and Moving the Speaker to a Stopover Position in Eliminating Location Cues

The movements of the speaker placed behind the screen were audible. We were concerned that participants might infer the position of the speaker from that sound. To foil the use of this cue, we played a masking sound during speaker movement and also moved the speaker to a stopover location before moving it to the target location. The purpose of this control experiment was to ensure that these two manipulations were effective in eliminating the location cue participants might use to infer the position of the speaker.

Participants. Three participants, recruited from New York University, participated exclusively in this control experiment (all females, aged 25-36 with mean age equal to 28, all right-handed, one of them was naive to the purposes of the experiment). None reported any visual, auditory or motor impairment. Experimental protocols were approved by the Institutional Review Board at New York University. All participants gave informed consent prior to the beginning of the experiment and volunteered to participate.

Procedure. The accuracy of judging the speaker's position was measured using a two-alternative forced-choice (2AFC) procedure. In each trial, the fixation was displayed at the center of the screen and the masking sound was playing during speaker movement. Every time the speaker moved, it first moved to a stopover location before it moved to the target location. After the speaker reached the target location, participants reported by keypress whether the speaker was to the left or right of the central fixation. Feedback was provided. The test locations were evenly spaced from -12.5 to 12.5° in steps of 5°. Each test location was repeated 40 times, resulting in a total of 240 trials. Participants took about an hour to complete the experiment.

Analysis. We first calculated the standard deviation of the number of correct responses for each location of the speaker using the formula $SD = \sqrt{np(1-p)}$, where p is the observed percent correct and n is the number of total trials for each location ($n = 40$) (**Fig. S11**). To determine whether participants showed significant above-chance performance, we performed a binomial test for each speaker's location. The null hypothesis is that the number

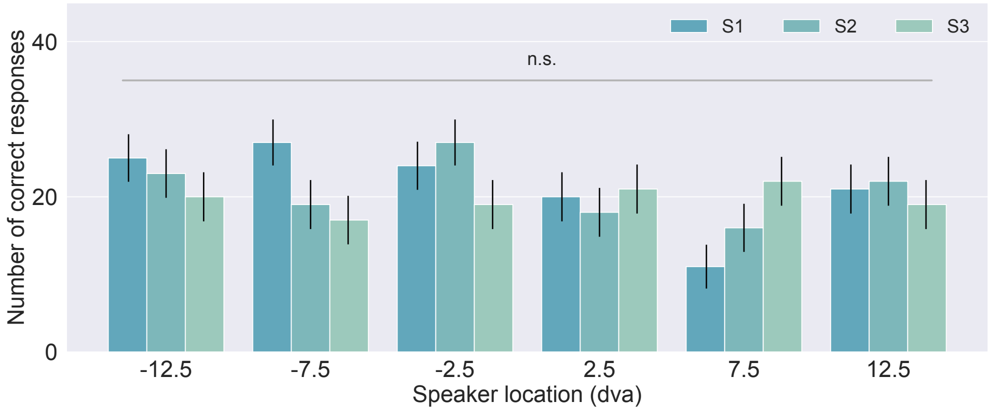

Figure S11. Results of the control experiment. Horizontal axis: the speaker location; vertical axis: the number of correct responses (out of a total of 40 trials) at a given speaker location. Color represents different participants. Error bars: ± SD.

of correct responses is not significantly better than that obtained by guessing, i.e., $H_0 : \pi \leq \pi_0$ where $\pi_0 = 0.5$. Then, we calculated the cumulative probability $P(X \geq k)$ using the following formula:

$$P(X \geq k) = \sum_{k=c}^{k=n} \frac{n!}{k!(n-k)!} {\pi_0}^k (1 - \pi_0)^{n-k},$$

where c is the number of correct responses at a given speaker location. Finally, we compared the cumulative probability with the Bonferroni-corrected *p*-value.

Results. The performance was not significantly greater than chance at any stimulus location for any of the participants (*p* > 0.05 with Bonferroni correction). In other words, there is no evidence that participants were able to infer the location of the speaker based on the noise generated during speaker movement. Therefore, we conclude that playing the masking sound and moving the speaker to an intermediate location were effective in eliminating the potential location cue during speaker movement.

S12. Examination of the recalibration gain over the course of the post-recalibration task

In this section, we examined whether it is reasonable to assume that measurement-shifts, accumulated during the audiovisual recalibration task, were fully maintained throughout the subsequent post-recalibration task. To this aim, we split the localization responses from the post-recalibration phase into two halves based on time scale, and then computed the recalibration gain for each half of the data, each modality, session and participant. More specifically, we first pooled the localization responses from the pre-recalibration phase of the six sessions, then fitted a linear regression to the pooled localization responses. Next, for each stimulus location, we computed the distance between each individual post-recalibration response and the value corresponding to the stimulus location on the linear regression fit to the pre-recalibration responses. Finally, we calculated the recalibration gain by taking an average of the distance across trials and stimulus locations. We didn't observe a consistent decrease of the recalibration effect in the second half of the post-recalibration phase (**Fig. S3**). Given that there is no indication for the recalibration effect to dissipate over time, our assumption about measurement-shifts being fully maintained throughout the post-recalibration task is valid.

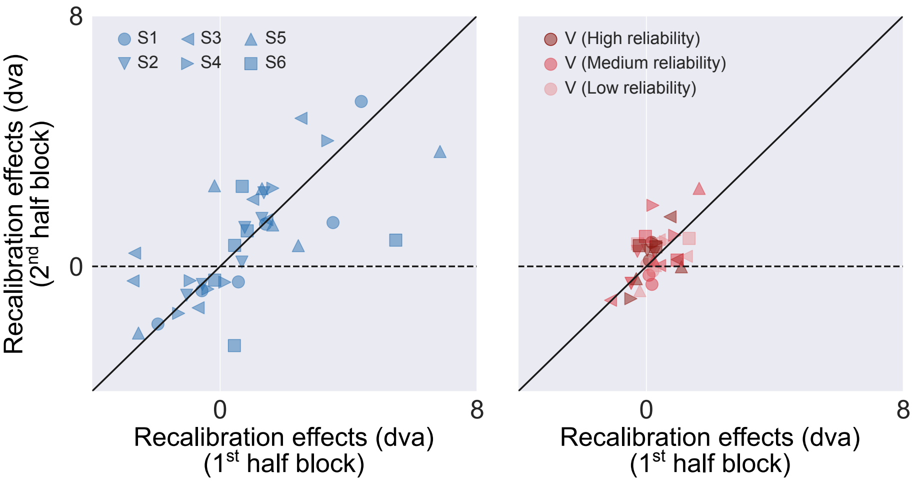

Figure S12. Recalibration effects computed separately for the first and the second half of the post-recalibration task. Blue: auditory; red: visual. Diagonal solid line indicates no change of the recalibration gain over the course of the task; horizontal dashed line indicates the recalibration gain completely wore off in the second half of the task.

S13. Qualitative predictions for audiovisual localization responses during the audiovisual recalibration phase

We made qualitative predictions for the audiovisual localization responses during the recalibration phase by simulating the amount of auditory measurement-shifts over the course of the recalibration phase for three different visual reliabilities. For simplicity, we assume there was only one audiovisual recalibration trial ($s_V = 0°$ and $s_A = -8°$), presented repeatedly during the recalibration phase. The auditory measurement-shift is updated on a trial-by-trial basis to compensate for cue discrepancy. At the end of the recalibration phase, the auditory recalibration effect rises with increasing recalibration update rate but is a non-monotonic function of visual reliability as a result of causal inference (**Fig. S13**).

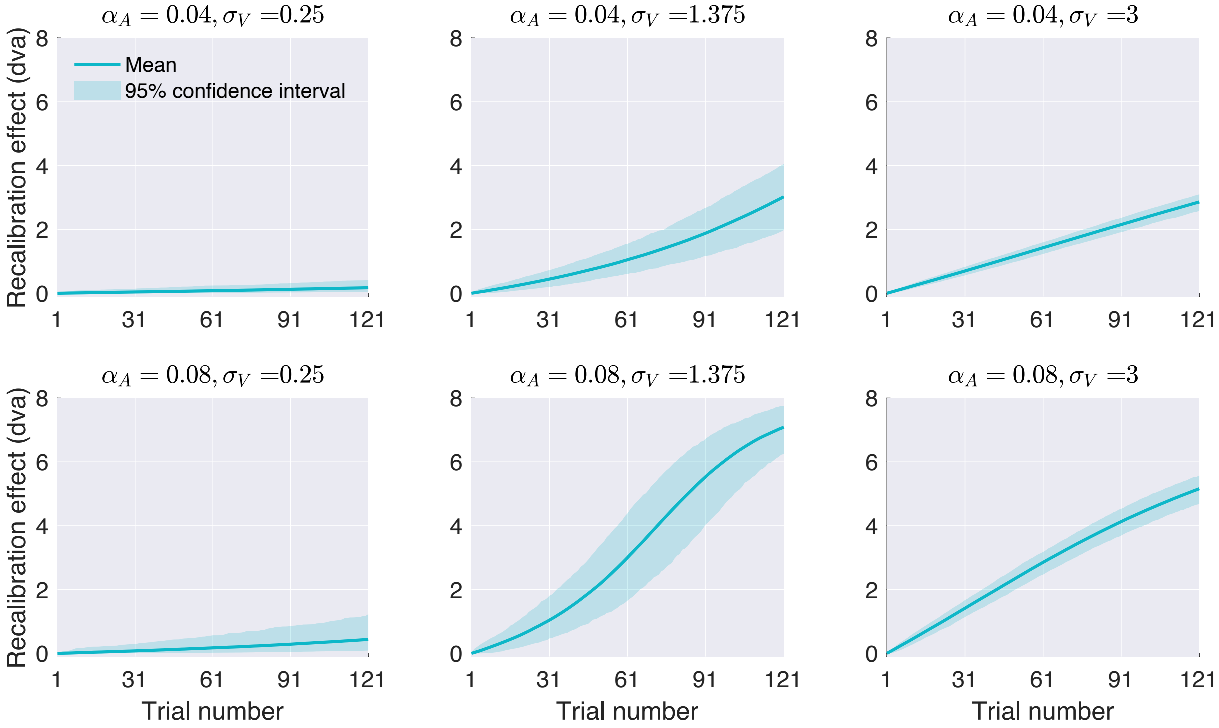

Figure S13. Simulated auditory recalibration effects during the audiovisual recalibration phase given a slow (top row) and a fast (bottom row) learning rate with high (left column), medium (middle column) and low (right column) visual reliability. Solid blue line: mean auditory recalibration effect; shaded blue area: 95% confidence intervals over 1,000 simulations.

S14. Issues Regarding the Estimation of the Reliability Under Bimodal Stimulus Presentation Given the Fixed-Ratio and the Reliability-Based Model

We assumed that the internal sensory noise differs between bimodal and unimodal stimulus presentation. We denoted $\sigma_{AV,A}$ and $\sigma_{AV,V}$ as the internal sensory noise when the auditory and visual stimulus were presented at the same time during the audiovisual recalibration task, and denoted σ'_A and σ'_V when the auditory or visual stimulus was presented unimodally. σ'_A and σ'_V were constrained by the data from the unimodal spatial-discrimination task. In contrast, $\sigma_{AV,A}$ and $\sigma_{AV,V}$ can be directly estimated based on the data from the audiovisual recalibration task, but those data were excluded from the model-fitting process due to sequential dependence of the measurement-shifts and the lack of a closed form solution for the models. However, these two parameters were fitted indirectly because they influence the width of the approximated probability distribution of measurement-shift updates and therefore the log likelihood of the model and candidate parameters (see **Eq. 48**). More specifically, the width of the approximated distribution of the measurement-shift updates is positively correlated with both the internal sensory noise $\sigma_{AV,A}$ and $\sigma_{AV,V}$, and the learning rate α (modality-specific for the fixed-ratio model and supra-modal for the reliability-based model, see **Eqs. 1-5**). As the internal sensory noise or the learning rate increases, the width of the approximated distribution of the measurement-shift updates increases. On the other hand, the mean of the approximated distribution, which indicates the predicted amount of recalibration, is directly determined by the learning rate only. As the learning rate increases, the amount of recalibration also increases.

In this section, we quantitatively show that there is a trade-off between $\sigma_{AV,A}$ and α_A, by computing the negative log likelihood of the fixed-ratio model and different combinations of $\sigma_{AV,A}$ and α_A while fixing the rest free parameters. Results showed that the negative log likelihood remains almost the same when either one parameter is relatively large (**Fig. S14A**), which supports our conjecture. The combination of these two parameters with the minimum negative log likelihood corresponds to a small α_A and an extremely large $\sigma_{AV,A}$, because having a small α_A captures the small amount of recalibration observed in the data, which

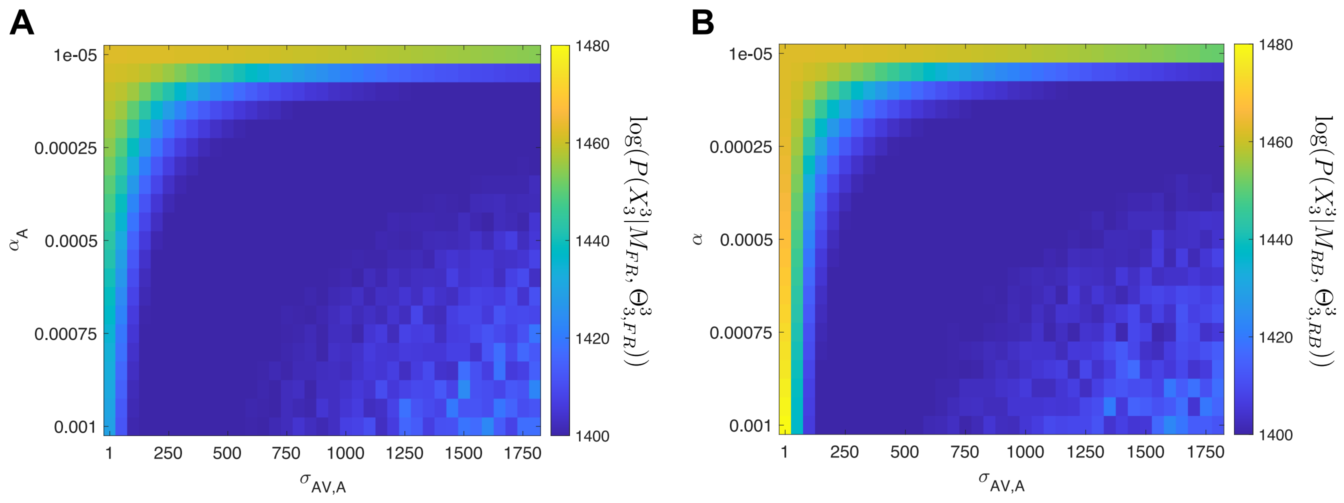

Figure S14. The negative log likelihood of the fixed-ratio model (**A**) or the reliability-based model (**B**) at different combinations of the internal sensory noise $\sigma_{AV,A}$ and a learning rate, given the localization responses of participant S1 from the post-recalibration task X_3^3.

consequently forces $\sigma_{AV,A}$ to be large. We also computed the negative log likelihood of the reliability-based model and different combinations of $\sigma_{AV,A}$ and the supra-modal learning rate α while fixing the rest of the free parameters, and found a trade-off between these two parameters as well (**Fig. S14B**). From these observations, the best-fit $\sigma_{AV,A}$ given either model is considerably high and it's impossible to have such a large internal sensory noise even if dividing attentional resources to two stimuli reduces stimulus reliability in the bimodal condition. Therefore, we set the upper boundary for $\sigma_{AV,A}$ and $\sigma_{AV,V}$ to be five times the mean σ'_A and σ'_V averaged across participants ($ub_{\sigma_{AV,A}}$=20, $ub_{\sigma_{AV,V_i}} = 5,\ 12,$ or $20,$ for the three visual-reliability conditions).

S15. Proportionality of Auditory and Visual Measurement-Shifts in the Reliability-Based and the Fixed-Ratio Models

For all three recalibration models, the updates to the measurement shifts accumulate over the 120 recalibration trials. For the reliability-based and fixed-ratio models, the updates to Δ_A are a fixed proportion of the updates to $\Delta_V(t)$ with a constant of proportionality that depends only on the specific visual-reliability condition. For example, in the reliability-based model, Δ_A is incremented by $\alpha w_i \left(m'_{V_i,l_V(t)} - m'_{A,l_A(t)}\right)$ (Eq. 11), while Δ_V is incremented by $\alpha(1-w_i)\left(m'_{A,l_A(t)} - m'_{V_i,l_V(t)}\right)$. At the end of the recalibration phase:

$$\frac{\Delta_A(121)}{\Delta_{V_i}(121)} = \frac{\sum_{t=1}^{120} \alpha w_i \left(m'_{V_i,l_V(t)} - m'_{A,l_A(t)}\right)}{\sum_{t=1}^{120} \alpha(1-w_i)\left(m'_{A,l_A(t)} - m'_{V_i,l_V(t)}\right)} = \frac{-w_i}{1-w_i}, \tag{S1}$$

where w_i depends on the visual-reliability condition i alone, and is thus constant across the block of 120 trials. The proof for the fixed-ratio model is nearly identical, with the fixed proportion being $\frac{\alpha_A}{\alpha_V}$. In contrast, for the causal inference model of recalibration, the visual and auditory updates depend on the probability of a common cause, which varies from trial to trial, resulting in the truly bivariate distribution of (Δ_A, Δ_V).

Reference

1. Waskom, M., et al., *Seaborn: statistical data visualization.* URL: https://seaborn. pydata. org/(visited on 2017-05-15), 2014.
2. Mateeff, S. and A. Gourevich, Peripheral vision and perceived visual direction. Biol Cybern, 1983. **49**(2): p. 111-8.
3. Adam, J.J., et al., Evidence for attentional processing in spatial localization. Psychological Research, 2008. **72**(4): p. 433-442.
4. Odegaard, B., D.R. Wozny, and L. Shams, Biases in Visual, Auditory, and Audiovisual Perception of Space. PLoS Comput Biol, 2015. **11**(12): p. e1004649.
5. Deveaux, R.D., Applied smoothing techniques for data analysis. 1999, Taylor & Francis.
6. Silverman, B.W., Density estimation for statistics and data analysis. 2018: Routledge.
7. Acerbi, L. and W.J. Ma. Practical Bayesian optimization for model fitting with Bayesian adaptive direct search. in Proceedings of the 31st International Conference on Neural Information Processing Systems. 2017.
8. Sato, Y., T. Toyoizumi, and K. Aihara, Bayesian inference explains perception of unity and ventriloquism aftereffect: identification of common sources of audiovisual stimuli. Neural Comput, 2007. **19**(12): p. 3335-55.
